# Utilizing Light to Control Glycopolymer–DC-SIGN Interactions via Molecular Motors

**DOI:** 10.1021/jacs.5c20077

**Published:** 2026-06-30

**Authors:** Caitlin L.A. Nutting, Adrien Combe, Gokhan Yilmaz, Richard Napier, Ben L. Feringa, C. Remzi Becer

**Affiliations:** † Department of Chemistry, 2707 University of Warwick, Coventry CV4 7AL, U.K.; ‡ Centre for System Chemistry, Stratingh Institute for Chemistry, 3647 University of Groningen, Nijenborgh 3, Groningen 9747AG, The Netherlands; § School of Life Sciences, 2707 University of Warwick, Coventry CV4 7AL, U.K.

## Abstract

Molecular motors, known for their ability to undergo controlled unidirectional rotation under external stimuli, have gained growing interest for their potential applications in biological systems and smart materials. Meanwhile, glycosylated macromolecules are utilized in lectin-binding, making them potential candidates to combat pathogenesis, cancer, and biofilms. However, glycopolymers are limited by their current inability to adapt in response to external stimuli, restricting their potential for future precision therapies. By integrating multivalent glycopolymeric structures with first-generation molecular rotary motors, it becomes possible to control carbohydrate–lectin interactions using intrinsic motor functions. Herein, we present photoadaptive glycopolymer-based first-generation molecular motors functionalized with immunologically important monosaccharides, β-d-glucopyranoside and β-d-mannopyranoside. The lectin-binding properties were subsequently investigated using surface plasmon resonance with DC-SIGN and Langerin, key players within the human innate immune system. Additionally, a competition assay with the envelope glycoprotein of HIV, namely gp120, was conducted to ascertain the inhibitory potential of these photoresponsive glycopolymer-based molecular motors. It was established that the light-controlled conformation of the molecular motor impacted both the overall binding affinity and half-maximal inhibitory concentration values. These findings create opportunities to control the binding affinity of glycopolymers and emphasize the potential of light-driven glycopolymers to modulate key interactions in cell-specific targeted delivery of therapeutics.

## Graphical abstract

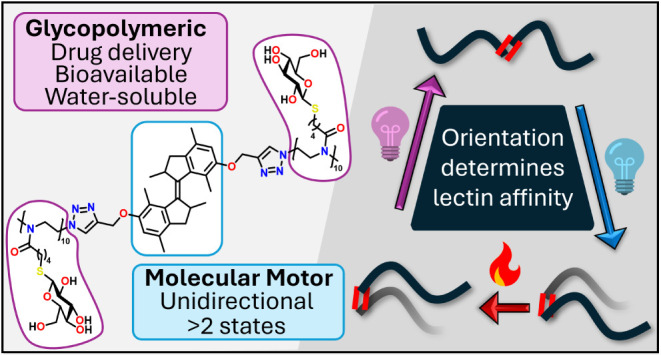

## Introduction

Carbohydrate-binding proteins, also known as lectins, play a key role in regulating and protecting cells.^1–3^ They are primarily located on the cell membranes and are essential for cell–cell communication and cellular regulation, serving as key components of the innate immune system and often acting as the first line of defense against glycan-rich invading pathogens.^4–6^ To effectively interact with and exploit lectins, multivalent carbohydrate-bearing macromolecular structures have been identified as promising candidates for cell-targeted recognition, offering enhanced interactions through the cluster glycosidic effect.^3,7,8^ Recent advances in synthetic design strategies to prepare and functionalize glycopolymers have enabled a broad range of applications, particularly in the biomedical and pharmaceutical fields, such as drug or nucleic acid therapeutic delivery systems, biosensors, drug conjugation, and bacteria/biofilm removal systems.^9–17^ However, one of the main challenges facing synthetic glycopolymers is their lack of selectivity and adaptivity toward specific lectins, in comparison to glycans and antibodies, which are significantly more challenging to synthesize at scale.^18,19^ As known from glycan design strategies, every tiny deviation in the structure can significantly affect their affinity for specific binding sites of lectins, due to highly precise hydrogen bonding motifs.^20^

Currently, the most advanced glycopolymers have been tailored to optimize their binding capabilities through modification of their stereochemistry,^21–23^ chain architecture,^19,24^ chain length,^11^ and flexibility.^25–27^ These features can be fine-tuned to the targeted lectin to induce the intended biological activity. Moreover, the stereochemistry of the polymeric backbone can also be manipulated *via* the orientation of the glycosyl residues, which can significantly influence the accessibility and presentation of the pendant sugar moieties.^22,28^ Furthermore, identifying the appropriate polymeric backbone and corresponding polymerization technique is crucial for fine-tuning properties such as flexibility and solubility, determining the material’s suitability for the desired application.

In this context, poly­(2-oxazoline)­s (POx) have emerged as a promising class of biomaterials that are recognized as an alternative to poly­(ethylene glycol) (PEG).^29–33^ POx has gained increasing attention due to its tunability, reduced cytotoxicity, and “stealth”-like properties, which are comparable to PEG and PEGylated drugs.^24,34–38^ These features make POx a highly attractive candidate when synthesizing glycopolymers for biomedical applications such as stabilization of lipid nanoparticles carrying nucleic acid therapeutics for gene delivery.^39^

External control of binding affinities, using photoadaptive units, would offer great opportunities for high-precision targeting. Currently, there is limited understanding of how the use of external stimuli can induce conformational changes of glycopolymers to modulate the position of carbohydrates and their ability to interact with lectins. Light has the advantage of being a noninvasive stimulus with high spatiotemporal control. Therefore, the insertion of a light-responsive building block into the glycopolymeric design has great benefits to lectin binding. Simple photoswitches, particularly azobenzenes and arylazopyrazole derivatives, have previously been applied in glycochemistry and specific lectin studies due to their high spatiotemporal control, reversible photo- and thermal reverse isomerization in aqueous media, as well as their ability to undergo multiple switching cycles.^40–43^ However, photoswitches are limited by the lack of control over their directional motion during photoisomerization and the thermal instability of the bent *Z*-isomer, which reverts to the more stable planar *E*-isomer.^44–46^

Conversely, first-generation light-driven molecular rotary motors are a favorable alternative to photoswitches with higher thermal stability of the *E* and *Z* isomers, greater control of motion due to their unidirectional rotation, and more conformational possibilities. The first-generation molecular motor core, characterized by a central overcrowded alkene core, has been shown to induce significant conformational differences through photoirradiation with the formation of four states: two stable and two metastable.^47–49^ The unidirectional rotation of molecular motors provides an enhanced control over the desired conformation.^50^ These properties have demonstrated utility in various applications ranging from supramolecular chemistry^51,52^ to material science.^53^

Herein, we demonstrate the ability to control the orientation of carbohydrate moieties utilizing a molecular motor, offering a unique way to manipulate the lectin-binding affinity through light stimulus. Toward this goal, a photoresponsive first-generation molecular motor with 20 and 22 repeat units, respectively of β-d-glucopyranoside- and β-d-mannopyranoside-containing poly­(2-oxazoline) (**P­(GlcOx)**_**20**_ **MM** and **P­(ManOx)**_**22**_ **MM**, Figure 1), was designed. Furthermore, their detailed lectin interactions have been studied using SPR with different human carbohydrate-binding proteins: Dendritic Cell-Specific Intercellular adhesion molecule-3-Grabbing Nonintegrin (DC-SIGN) and Langerin (Figure 1). Finally, the inhibition in solution assays were conducted to calculate the half-maximal inhibitory concentration (IC_50_) of photoadaptive glycopolymer-molecular motor conjugates.

**Figure 1:**
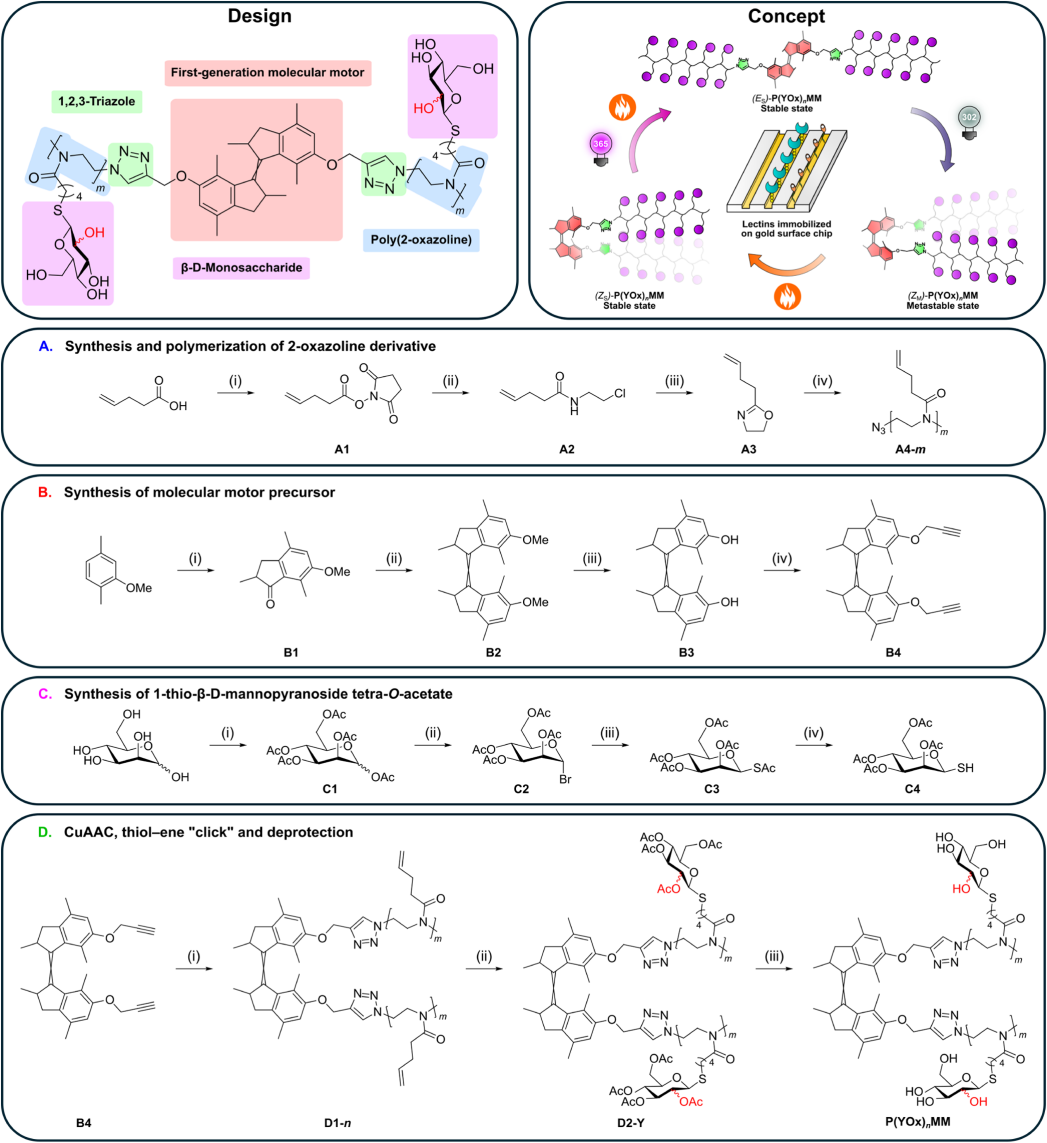
**(Design)** Macromolecular design of glucoside- and mannoside-containing poly­(2-oxazoline)-based first-generation molecular motors: **P­(GlcOx)_20_MM** and **P­(ManOx)_22_MM**, respectively. **(Concept)** Schematic illustration of the wavelength-controlled unidirectional rotation of molecular motors **P­(GlcOx)_20_MM** or **P­(ManOx)_22_MM** and their interactions with lectins immobilized on a gold-coated surface of a surface plasmon resonance (SPR) chip. (a) (i) NHS, EDAC, DCM, 0 °C, 24 h, 87%. (ii) 2-Chloroethylamine, Et_3_N, DCM, 0 °C, 24 h, 99%. (iii) KOH, MeOH, 50 °C, 24 h, 41%. (iv) MeOTs, NaN_3_, MeCN, 110 °C, 16 h, **A4–10**: 89%, **A4–11**: 60%, **A4–22**: 93%. (b) (i) Methacrylic acid, PPA, 100 °C, 4 h, 64%. (ii) TiCl_4_, Zn_(s)_, THF, 70 °C, 3 d, 72%. (iii) MeMgI, 140/160 °C, 3/16 h, *E*: 38%, *Z*: 50%. (iv) Propargyl bromide in toluene, Cs_2_CO_3_, MeCN, 60 °C, 16 h, *E*: 87%, *Z*: 82%. (c) (i) Ac_2_O, Pyridine, 0 °C, 16 h, 89%. (ii) HBr in AcOH, DCM, r.t., 16 h, 73%. (iii) AcSK, DMPU, r.t., 3 d, 79%. (iv) DTT, NaHCO_3_, DMA, r.t., 2 h, 43%. (d) (i) **A4-** *m*, CuSO_4_·5H_2_O, sodium L-*(+)*-ascorbate, DMF, r.t., 48 h, *E*-**D1–20**: 42%, *Z*-**D1–20**: 46%, *E*-**D1–22**: 38%, *Z*-**D1–22**: 43%. (ii) **C4** or 1-thio-β-d-glucopyranoside tetra-*O*-acetate, V601, MeCN, 70 °C, 48 h. (iii) MeONa, MeOH, r.t., 6 h, *E*-**P­(GlcOx)_20_MM**: 65%, *Z*-**P­(GlcOx)_20_MM**: 82%, *E*-**P­(ManOx)_22_MM**: 23%, *Z*-**P­(ManOx)_22_MM**: 40%, yields based from **D1-** *n*. Y = Glc or Man, *m* = 10, 11, or 22, *n* = 2 × *m* = 20 or 22.

## Results and Discussion

### Synthesis of Glycopolymer-Based Molecular Motors

The full synthetic pathways toward the synthesis of **P­(GlcOx)**_**20**_ **MM** and **P­(ManOx)**_**22**_ **MM** are outlined in Figure 1A–D. Initially, the oxazoline-derived monomer **A3** was prepared according to a procedure previously reported by Schlaad’s *et* *al*.^100^ (Figure 1A, Scheme S1). The different polymers **A4-** *m* (*m* = 10, 11, or 22) were synthesized using cationic ring-opening polymerization (CROP) *via* a one-pot method. The polymers were obtained using [M]/[I] ratios between 10 and 22 (Figures S33–S35), which subsequently reached full conversion, confirmed by the shift of proton signals on the 2-oxazoline ring with proton nuclear magnetic resonance (^1^H NMR) spectrometry (δ_H_ = 4.15 and 3.75 ppm) (Figures S36–S38). The resulting polymers were further analyzed *via* gel permeation chromatography (GPC) to confirm their molar mass and dispersity (Figures S55–S57), in addition to matrix-assisted laser desorption/ionization with time-of-flight (MALDI-ToF) mass spectrometry (Figures S72–S75) and Fourier-transform infrared (FT-IR) spectroscopy (Figure S66–S71).

After the successful synthesis of the poly­(2-oxazoline)-derived precursors **A4-** *m*, bis-hydroxy motor **B3** was prepared using a three-step synthetic route (Figure 1B, Scheme S2) with its *E* and *Z* isomers separated, following our previously established procedures.^52–54^ To enable polymer attachment, the bis-hydroxy motor **B3** was subjected to Williamson ether formation with propargyl bromide to afford the bis-propargyl motor **B4**, which introduced two alkyne moieties serving as anchoring points for subsequent polymer conjugation. Each isomer of **B4** was fully characterized *via* ^1^H NMR (Figures S20, S22), ^13^C NMR (Figures S21, S23), and FT-IR spectroscopies (Figures S63, S64).

Then, the mannopyranoside-derived precursor **C4** was successfully synthesized *via* a four-step synthetic pathway (Figure 1C, Scheme S3), following adapted procedures.^22,54^ Compound **C4** has been characterized by ^1^H NMR (Figure S31), ^13^C NMR (Figure S32), and FT-IR spectroscopy (Figure S65). In parallel, its corresponding glucopyranoside was purchased from Sigma-Aldrich.

Next, polymeric precursors **A4–10** and **A4–11** were then subsequently “clicked” onto both propargyl groups of motor **B4** using copper-catalyzed azide–alkyne cycloaddition (CuAAC) to afford molecular motor-derived **D1-** *n* (*n* = 20 or 22). The successful conjugation was then confirmed by the evident appearance of CH group observed at around 6.80 ppm shown in the ^1^H NMR spectra (Figures S38–S41) as well as by the increase in molar mass calculated from the shift in the GPC traces (Figures S55–S56 and Figures S59–S61). Polymer derivatives **D1-** *n* were then glycosylated *via* a thiol–ene “click” reaction, whereby 1-thio-β-d-glucoside or 1-thio-β-d-mannoside moieties were attached to the terminal alkenes. The completion of the click reaction was monitored by the disappearance of the alkene double bond peak at 5.78 ppm region in the ^1^H NMR spectra. Finally, after deacetylation and purification *via* dialysis, the desired molecular motor conjugates, **P­(GlcOx)**_**20**_ **MM** and **P­(ManOx)**_**22**_ **MM,** were obtained. The compounds were fully characterized using ^1^H and ^13^C NMR spectrometry (Figure 2A,B and Figures S43–S54), MALDI-ToF mass spectrometry (CuAAC) (Figure 2C), FT-IR spectroscopy (Figure 2D, Figures S58, S63), and GPC (Figure 2E).

**Figure 2:**
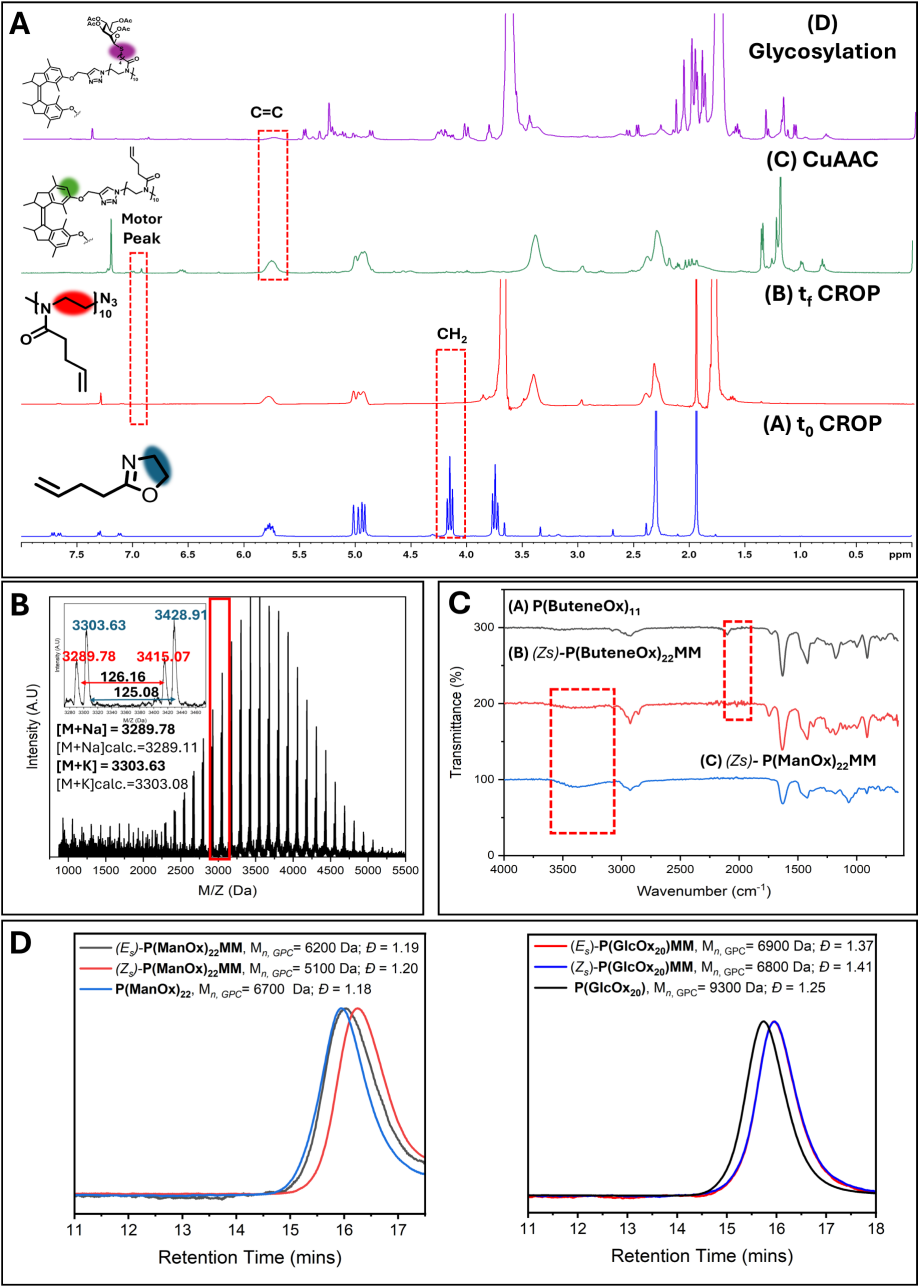
Characterization of the synthesized molecular motors. For clarity, only *(Z)* **-P­(ManOx)**_**22**_ **MM** is presented here. The full charcterization of the other analytes can be found within the Supporting Information within Figure S38-S74.(A) ^1^H NMR stacked spectra illustrating each step in the formation of the glycosylated molecular motors *(Z)* **-P­(ManOx)**_**22**_ **MM**. (B) The MALDI-ToF mass spectra of the clicked motor and polymer of *(Z)* **-P­(ManOx)**_**22**_ **MM**. (C) The stacked FT-IR spectrum showing the progression of the reaction toward the final glycopolymer *(Z)* **-P­(ManOx)**_**22**_ **MM**. (D) GPC traces of the final glycopolymers, where left traces reflect the final mannoside and right traces reflect the glucoside-containing glycopolymer-molecular motor conjugates.

All details of the complete characterization of the final compounds, in addition to the isolated intermediates, are presented in the Supporting Information, while the representative characterization data are shown in Figure 2.

### Determination of the Critical Aggregation Concentration (CAC) Values

The water solubilities of stable *E* and *Z* isomers of both **P­(GlcOx)**_**20**_ **MM** and **P­(ManOx)**_**22**_ **MM** along with their respective controls **P­(GlcOx)**_**22**_ and **P­(ManOx)**_**22**_ were evaluated in a HEPES-buffered saline (HBS) solution (HEPES 10 nM, NaCl, 150 mM, CaCl_2_ 5 mM, pH 7.40) using the Nile Red fluorescent assay (NRFA) method to determine their critical aggregation concentration (CAC). The CAC value is an essential parameter for SPR measurements, as aggregated macromolecules can artificially inflate the *R*_max_ value, potentially changing the interpretation of the absolute binding affinity. Although *R*_max_ is not directly linked to the overall binding parameters, it is important to mitigate this to ensure the accuracy of the analysis.

For the glucoside-containing molecular motor (**P­(GlcOx)**_**20**_ **MM**), the *Z* isomer exhibited a CAC of 47.3 μm (Figure S1a), which is twice as high as that of the *E* isomer (19.1 μm) (Figure S1b). Both values are significantly lower than the CAC of the corresponding control **P­(GlcOx)**_**22**_ (Figure S1c). Such a difference provides evidence that the incorporation of a hydrophobic molecular motor unit in the center of the POx greatly decreases the hydrophilicity of the overall structure. Similarly, the *Z* isomer of the mannoside-containing molecular motor (**P­(ManOx)**_**22**_ **MM**) had a CAC value of 33.0 μm (Figure S2b), while the *E* isomer exhibited a lower CAC value of 25.0 μm (Figure S2a). Both values were lower than those of the mannoside-containing control **P­(ManOx)**_**22**_, which was observed to be 314 μm (Figure S2c). As previously, the values indicate that the incorporation of a hydrophobic component decreased the hydrophilicity of the overall structure. All analytes appear to self-assemble into small spherical micelles of 5–10 nm diameter (Figure S3).

### Rotation of the Molecular Motor

The described first-generation molecular motor undergoes a 360° unidirectional rotation through four distinct steps, completing the full cycle of both **P­(YOx)**_***n***_ **MM** (Y = Glc or Man, *n* = 20 or 22, respectively), starting from the stable *Z*_S_ isomers (*(Z*_S_)-**P­(YOx)**_***n***_ **MM**, Figure 3).^47–49^ This cycle includes two photochemical and two thermal isomerization steps.

**Figure 3:**
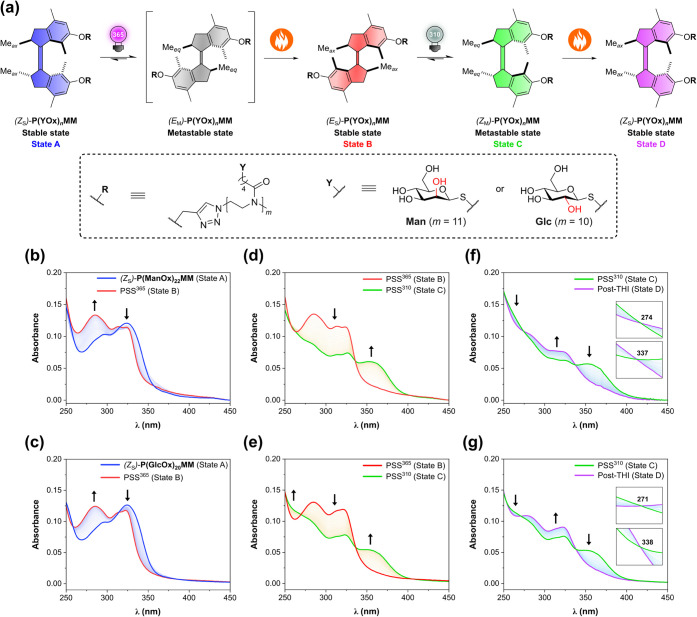
Rotational analysis of the synthesized molecular motors **P­(GlcOx)_20_MM** and **P­(ManOx)_22_MM**. (a) Scheme of the complete unidirectional rotary cycle of molecular motors **P­(YOx)*_n_* MM** starting from *(Z*_S_)-**P­(YOx)*_n_* MM**. *n* corresponds to the number of **YOx** repeat units, where *n* = 20 (for Y = Glc) or *n* = 22 (for Y = Man). All stereogenic centers are relative. (b,c) UV–Vis spectra of pure stable (b) *(Z*_S_)-**P­(GlcOx)_20_MM** and (c) *(Z*_S_)-**P­(ManOx)_22_MM** upon 365 nm light irradiation for 1 h converting to stable *(E*_S_)-**P­(GlcOx)_20_MM** and *(E*_S_)-**P­(ManOx)_22_MM**, respectively, as PSS^365^ mixtures (5 μM in HBS solution, below CAC, 20 °C). (d,e) UV–Vis spectra of PSS^365^ mixture from **(d) P­(GlcOx)_20_MM** and **(e) P­(ManOx)_22_MM** upon 310 nm light irradiation for 1 h converting to metastable *(Z*_M_)-**P­(GlcOx)_20_MM** and *(Z*_M_)-**P­(ManOx)_22_MM**, respectively, as PSS^310^ mixtures (5 μM in HBS solution, below CAC, 70 °C). (f,g) Thermal helix inversion (THI) process from (f) *(Z*_M_)-**P­(GlcOx)_20_MM** and (g) *(Z*_M_)-**P­(ManOx)_22_MM** to convert to *(Z*_S_)-**P­(GlcOx)_20_MM** and *(Z*_S_)-**P­(ManOx)_22_MM**, respectively, as post-THI mixtures (5 μΜin HBS, below CAC, 70 °C).

The cycle begins when the stable *Z*_S_ isomer is irradiated at a wavelength of 365 nm. The metastable isomer *(E*_M_)-**P­(YOx)**_***n***_ **MM**, obtained after irradiation of the stable isomer *(Z*_S_)-**P­(YOx)**_***n***_ **MM**, undergoes fast thermal helix inversion (THI) to afford the stable *(E*_S_)-**P­(YOx)**_***n***_ **MM** and has a very short half-life time to be observed and analyzed under ambient conditions, leading to its exclusion from further study.^47,48^ Experimentally, an HBS solution containing the stable *Z*_S_ isomer (State A) was irradiated with 365 nm light to produce a photostationary state (PSS^365^ mixture, State B) predominantly composed of the stable *E*_S_ isomer (Figure 3A, State A and B). In stable states, the methyl groups in α-position from the central olefin of the motor moiety are in pseudoaxial orientation, minimizing steric hindrance and stabilizing the conformation. Subsequent irradiation at 310 nm converted the solution to a second photostationary state (PSS^310^ mixture, State C) that contained a majority of the metastable isomer *(Z*_M_)-**P­(YOx)**_***n***_ **MM**. In the metastable states, the methyl group adopts a pseudoequatorial position, which increases steric hindrance and induces a distinct conformation. To facilitate the slow THI of the metastable *Z*_M_ isomer back to the initial stable *Z*_S_ state, the solution was heated at 70 °C, resulting in a final mixture predominantly containing the stable *Z*_S_ isomer (Post-THI mixture, State D). Because this step theoretically involves only one reaction (*Z*_M_ → *Z*_S_), clear isosbestic points are observed, indicating the absence of obvious degradation of the molecular motor during this thermal process. Overall, these glycosylated molecular motors successfully complete a full unidirectional rotation cycle in aqueous media. The ultraviolet–visible (UV–Vis) spectra of the isomers resulting from each step of the cycle are presented in Figure 3.

Additional investigations and characterizations of the rotation of the molecular motors, including Eyring analysis, are also provided in the ESI (Figures S4 and S5 and Table S1).

### Lectin Binding Evaluation and Analysis

SPR was used to evaluate the lectin-binding capabilities of both molecular motors **P­(GlcOx)**_**20**_ **MM** and **P­(ManOx)**_**22**_ **MM**, as well as the nonphotoresponsive motor-free glycopolymers **P­(GlcOx)**_**22**_ and **P­(ManOx)**_**22**_ as controls. Affinities were determined for two biologically relevant human lectin types (DC-SIGN and Langerin), selected due to their critical roles within the innate immune system as well as their preference for mannoside and glucoside for binding.^55^

The analyte was passed over the substrate using a calcium-containing HBS solution (HEPES 10 mM, NaCl 150 mM, CaCl_2_ 5 mM, pH 7.40) at 25 °C, and the different concentrations of the analyte (16.0 to 0.5 μM) were selected to be below their CAC values (Figures S1, S2). It should be noted that, to ensure accurate correlation between the isomer state and binding behavior, all experiments were carried out in the dark, and measurements were taken straight after irradiation to mitigate any relaxation.

The SPR kinetic data were evaluated using the Langmuir 1:1 binding model, which assumes a simple reversible interaction between a single receptor and a single ligand. We acknowledge that this does not properly account for the kinetics of polymeric binding, but with no kinetic model suited to this application, we present 1:1 kinetic data as a proxy for affinity in these complex Velcro-like interactions. As a result, we present derived kinetic values as observed values,^56^ and we caution readers not to overinterpret the *K*_d obs_ values but rather to consider the overall binding pattern, which highlights changes in binding kinetics dependent on the conformation of the molecular motor.

Across all experiments, both **P­(GlcOx)**_**20**_ **MM** and **P­(ManOx)**_**22**_ **MM,** including their different rotational states and conformations, were found to bind to both DC-SIGN (Figures 4, S7, S9) and Langerin (Figures S8, S10). The quantitative values are presented in Table 1. The data demonstrates that **P­(GlcOx)**_**20**_ **MM** and **P­(ManOx)**_**22**_ **MM** exhibit distinct patterns of association and dissociation between the stable and metastable states. These differences are particularly visible with the curvature of the sensorgrams during the dissociation phase, a reflection of the stability of the binding interaction. Notably, each sensorgram displayed characteristic fast association and slow dissociation, typical for multivalent carbohydrate interactions, whereby multiple glycan–lectin interactions act cooperatively to enhance the overall affinity (Figures S7–S10, Table 1).^57^

**Figure 4:**
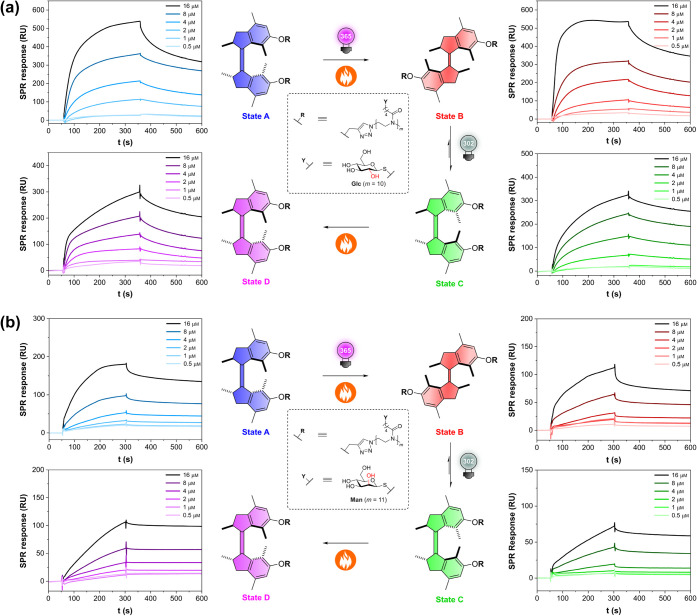
Illustration of some of the sensorgrams as the molecular machine is rotated. (a) Sensorgrams, measured using SPR, depicting the association and dissociation rates of the different cycle states of the molecular motor **P­(GlcOx)_20_MM** with DC-SIGN. (b) Sensorgrams, measured using SPR, depicting the association and dissociation rates of the different cycle steps of the molecular motor **P­(ManOx)_22_MM** with DC-SIGN.

**Table 1:** Kinetic Data of the Glucose- and Mannose-Containing Glycopolymers for Both DC-SIGN and Langerin^a^

Analyte	Carbohydrate	*R*_max_ (RU)	*k*_a obs_ (m^–1^ s^–1^)	*k*_d obs_ (s^–1^)	*K*_d obs_ (m)	Chi^2^
**DC-SIGN**						
*(Z)*-**P(GlcOx)**_**20**_ **MM** (State A)	Glucoside	526	525	1.47 × 10^–3^	**2.80 × 10** ^**–6**^	369
PSS^365^ mixture (State B)	Glucoside	496	519	1.44 × 10^–3^	**2.78 × 10** ^**–6**^	899
PSS^302^ mixture (State C)	Glucoside	358	440	6.57 × 10^–4^	**1.49 × 10** ^**–6**^	82.6
Post-THI mixture (State D)	Glucoside	320	406	9.76 × 10^–4^	**2.40 × 10** ^**–6**^	119
*(Z)*-**P(ManOx)**_**22**_ **MM** (State A)	Mannoside	161	651	1.94 × 10^–3^	**2.98 × 10** ^**–6**^	122
PSS^365^ mixture (State B)	Mannoside	130	578	1.31 × 10^–3^	**2.52 × 10** ^**–6**^	66.7
PSS^302^ mixture (State C)	Mannoside	91	494	4.76 × 10^–4^	**9.64× 10** ^**–7**^	8.65
Post-THI mixture (State D)	Mannoside	149	371	5.25 × 10^–4^	**1.42 × 10** ^**–6**^	38.1
**Langerin**						
*(Z)*-**P(GlcOx)**_**20**_ **MM** (State A)	Glucoside	245	738	2.98 × 10^–3^	**4.04 × 10** ^**–6**^	79.1
PSS^365^ mixture (State B)	Glucoside	229	1430	3.57 × 10^–3^	**2.49 × 10** ^**–6**^	133
PSS^302^ mixture (State C)	Glucoside	81.9	791	1.26 × 10^–4^	**1.59 × 10** ^**–7**^	35.2
Post-THI mixture (State D)	Glucoside	150	1090	1.09 × 10^–3^	**1.00 × 10** ^**–6**^	71.9
*(Z)*-**P(ManOx)**_**22**_ **MM** (State A)	Mannoside	195	611	5.88 × 10^–3^	**9.62 × 10** ^**–6**^	159
PSS^365^ mixture (State B)	Mannoside	261	356	5.76 × 10^–3^	**1.62 × 10** ^**–5**^	133
PSS^302^ mixture (State C)	Mannoside	59	956	3.07 × 10^–3^	**2.76 × 10** ^**–6**^	21.2
Post-THI mixture (State D)	Mannoside	44.1	1320	2.94 × 10^–3^	**2.23 × 10** ^**–6**^	21.3

a The chi^2^ value reflects the statistical goodness of fit for the modelled (Langmuir 1:1) data to the raw data.

Overall, the SPR results confirm that the *Z*_S_ isomer state shows enhanced interactions with both lectin types reflected primarily in their smaller observed *k*_d obs_ values (slower dissociation rates), resulting in a stronger binding affinity. Initially, we hypothesized that the *E*_S_ isomer of both glycopolymers would display the highest affinity due to reduced steric hindrance among the two glycopolymeric chains and therefore improved accessibility of the glycosyl residues. However, the experimental data does not support this hypothesis. This was shown during the rotation that the *E*_S_-enriched state (State B) demonstrated approximately 2-fold higher observed *K*_d obs_ values (lower affinities) compared to the *Z*_S_ state (States C and D) for binding to DC-SIGN and Langerin for glycoside-containing polymers. This difference was more pronounced for the mannoside-containing polymers binding with Langerin , where a 3–5-fold difference was observed. Interestingly, the experiment revealed that the lectin affinity was dependent on the motors’ geometry, demonstrating the importance of the position and availability of the pendant glucosides. Generally, the best binding affinity was shown in PSS^302^ mixtures (State C), closely followed by post-THI mixtures during the rotation (State D, Table 1). It is interesting to note that, in some cases, the glucose-containing compounds demonstrate an enhanced binding affinity. Glucoside and mannoside are both able to bind to C-type lectins due to the positioning of their C-3 and C-4 hydroxy groups, which coordinate with calcium. When comparing state by state for each sugar, mannoside-containing compounds have always shown higher association rate constants. Interestingly, it is the dissociation rate constants which demonstrate that the glucoside-containing compounds have enhanced binding complex stability. Some potential reasons for the enhanced binding may be due to reduced steric hindrance at the C-2 position on glucose.^58^

### Langerin Binding Affinity

Langerin, a pattern recognition lectin,^59,60^ binds to all conformations of both **P­(GlcOx)**_**20**_ **MM** and **P­(ManOx)**_**22**_ **MM** (Figures S8–S10). The binding affinity toward Langerin changes throughout the rotary cycle for both carbohydrates (Figure 5, Table 1).

**Figure 5:**
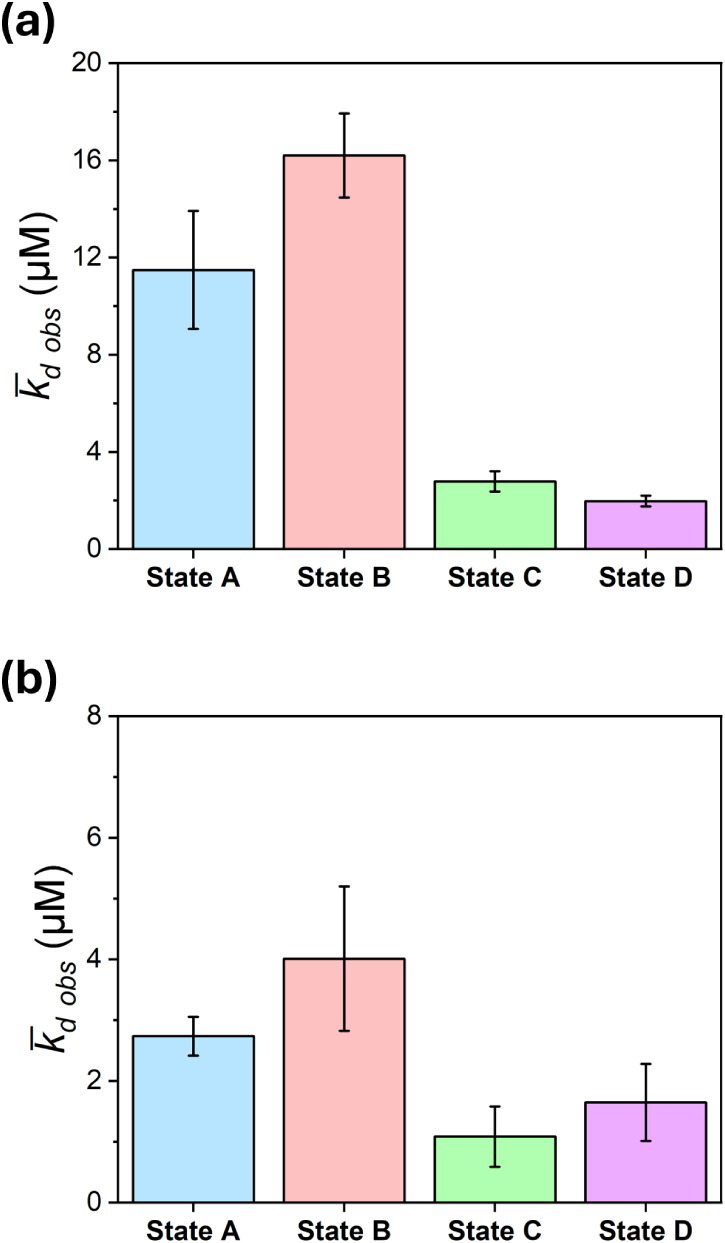
Statistical evaluation of the average *K*_d_ values of the mannoside-containing glycopolymers toward both lectin types where *N* = 3 and *p* = 0.05. (a) Demonstrates the binding and statistical evaluation of the binding toward Langerin. (b) Demonstrates the binding and statistical evaluation of the binding toward DC-SIGN. The results highlight the key differences between States B and C.

For the glucoside-containing molecular motors, both *E*_S_-enriched analytes (State B) exhibited somewhat higher association rates and 2-fold faster dissociation rates, suggesting a less stable binding interaction. In common with binding to DC-SIGN, the State C demonstrated the most stable binding to Langerin, followed by State D. These changes in binding strength are consistent with the suggestion that tiny changes in orientation in the metastable *Z*_M_ isomer exhibit enhanced interactivity. This difference between States C and D (before and after THI) offers a highly favorable structural environment for the interaction between glycosylated molecular motors and Langerin, improving the stability of the multivalent interaction, slowing dissociation, and increasing the overall affinity.

For the mannoside molecular motor, the *E*_S_-enriched mixture (State B) demonstrates a 2-fold faster dissociation rate, as well as a faster association rate compared to the *Z*_S_-enriched mixtures (States A and D). As a result, State B demonstrates the poorest affinity to Langerin, with a 5-fold difference exceeding the affinity difference observed for the glucoside-based molecular motor.

### DC-SIGN Binding Affinity

Dendritic cell-specific intercellular adhesion molecule-3-grabbing nonintegrin (DC-SIGN), is a C-type lectin responsible for cell–cell adhesion and internalization of viral structures,^5,61–63^ shows binding to all four states of both **P­(GlcOx)**_**20**_ **MM** and **P­(ManOx)**_**22**_ **MM** (Figure 4A and B, respectively). For both sugar types, the binding affinity changed throughout the cycle. This is reflected in both the curvature and magnitudes of the sensorgrams and binding kinetics (Figure 5, Table 1).

In the case of the glucoside-based molecular motor, similar to Langerin, *E*_S_-enriched analytes (pure *E*_S_ isomer and State B) demonstrated a 2-fold poorer affinity toward DC-SIGN compared to *Z*_S_- or *Z*_M_-enriched mixtures (States C and D, Table 1). Interestingly, States C and D exhibited enhanced affinity due primarily to reduced dissociation constants. Again, this suggests that subtle differences in the orientation of the sugars confer differences in the stability of binding.

For the mannoside-based molecular motor, the State C configuration demonstrated the highest affinity (*K*_d obs_), followed by the State D. This trend mirrors the glucoside-based molecular motor binding results and highlights that lectin affinity and stable binding may not be solely due to the absolute geometry.

### Competition Assay between Glycopolymer, DC-SIGN, and gp120

DC-SIGN is a target for many glycan-rich viruses such as human immunodeficiency virus (HIV),^62,64^ hepatitis C virus (HCV),^65,66^ and the Ebola virus.^67,68^ It has been suggested that glycopolymers could inhibit invading viruses such as HIV by preventing the interaction between gp120, the glycan-rich capsid protein, and cell surface-bound DC-SIGN.^69^ In this study, we describe the competition assay conducted using SPR to evaluate the potency of the synthesized glycopolymers in blocking the binding between DC-SIGN and gp120.

Glycoprotein gp120 was attached at a reduced surface density (*R*_max_ = 150 RU) to the CM5 chip *via* EDC coupling. Both **P­(GlcOx)**_**20**_ **MM** and **P­(ManOx)**_**22**_ **MM** and their corresponding *E*_S_ and *Z*_S_ states were evaluated (Figure 5). An initial kinetics was carried out between gp120 and DC-SIGN to obtain the best concentration of DC-SIGN to use for the competition assay (Figure S15). *R*_max_ values at each different concentration were used to calculate the half-maximal inhibitory concentration (IC_50_) values (Figures S16–S19).

Both sugar types demonstrated similar trends in potency with the *E*_S_ isomers (Figure 6, left-hand panels, and Table 2) giving the lowest IC_50_ values and the *Z*_S_ isomer higher values (Table 2). This indicates that far higher concentrations of *Z*_S_ polymer are needed to prevent DC-SIGN from binding to gp120; hence, the *E*_S_ isomers were better competitors and better potential therapeutics than the *Z*_S_ isomers.

**Figure 6:**
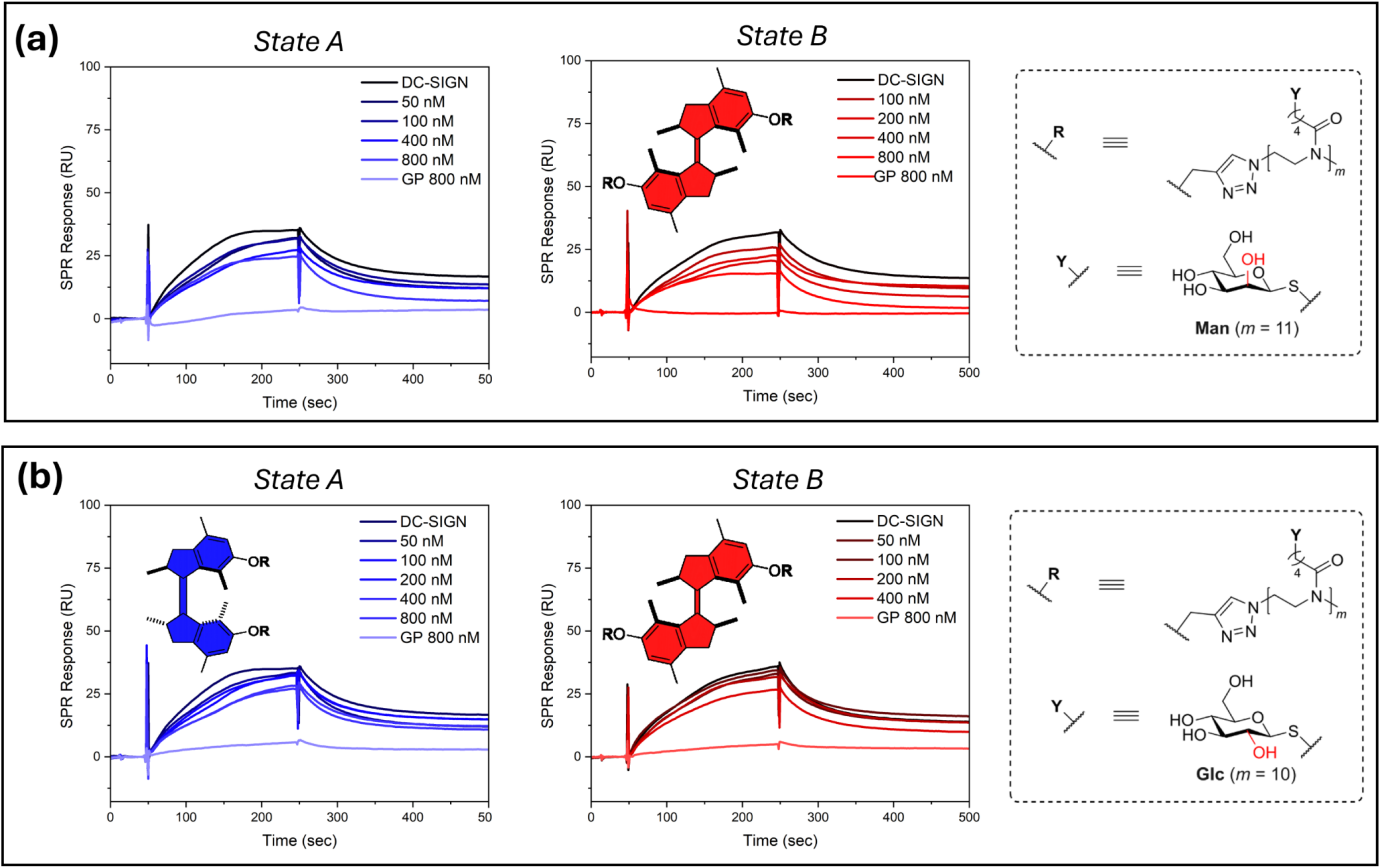
An example of the inhibition assay SPR sensorgrams, whereby the synthesized glycopolymers were competing with gp120 for DC-SIGN. As the polymer concentrations increased, the binding reduced between DC-SIGN and gp120. (a) The *Z*_S_ states (State A) and *E*_S_ (State B) of **P­(ManOx)_22_MM** sensorgrams demonstrating the reduction in the DC-SIGN response as polymer concentrations, the polymer was run by itself to demonstrate no binding. (b) The *E*_S_ and *Z*_S_ states of **P­(GlcOx)_20_MM** sensorgrams demonstrating the reduction in the DC-SIGN response as polymer concentrations, the polymer was run by itself to demonstrate no binding.

**Table 2:** Calculated IC_50_ Values Taken from the Relative *R*_max_ Change (%) Values Plotted vs Concentration (nM) for the Glucose and Mannose Containing Glycopolymers Binding to DC-SIGN in Solution to Compete with DC-SIGN Binding to gp120 on the Chip (Graphs Shown in Figures S16–S19)­^a^

Analyte	IC_50_ (nm)	*R* ^*2*^
**Glucoside**		
*(Z)*-**P(GlcOx)**_**20**_ **MM** (State A)	1620	0.87
PSS^365^ mixture (State B)	787	0.98
PSS^302^ mixture (State C)	942	0.93
Post-THI mixture (State D)	1740	0.62
**Mannoside**		
*(Z)*-**P(ManOx)**_**22**_ **MM** (State A)	1530	0.85
PSS^365^ mixture (State B)	521	0.69
PSS^302^ mixture (State C)	749	0.77
Post-THI mixture (State D)	612	0.45

a *R* ^2^ is the statistical fit for the plots in Figures S18 and S19.

This result contrasts with the SPR data which showed the *E* isomer during the rotation exhibited weaker affinities for DC-SIGN. Hence, it was anticipated that the E isomers would not bind more readily to DC-SIGN and would not be more effective at blocking the binding to gp120 on the chip. However, this is not the case. In this light, it is helpful to consider the different nature of the two assays. The SPR kinetic experiments record the rapid dynamics of binding between polymer in solution and DC-SIGN on the chip. The IC_50_ experiment is an end point titration of DC-SIGN following equilibration with the polymer in solution, thereby assessing the availability of DC-SIGN after polymer interaction and, in turn, the inhibitory concentration of the polymer. Nevertheless, it is not possible to fully explain the different behaviors recorded in the two assay systems. It is interesting to reflect that the competition assay more closely mimics the mixed environment of polymer action in a complex biological system. The distinct stable diastereomers behave differently, and the data are all consistent with our hypothesis that light-induced changes in orientation and, therefore, the steric hindrance influences their potency.

## Conclusion

Herein, we present the first reported photoadaptive glycopolymer-based molecular motors with conformation-selective lectin interaction behavior. A full macromolecular analysis has been presented along with documentation demonstrating the conformational changes of molecular motors carrying two glycopolymeric chains. Moreover, a detailed SPR analysis was carried out to evaluate the influence of all molecular motors’ conformations on DC-SIGN and Langerin lectin binding affinity. Among the different steps of the cycle, the metastable *Z*_M_-containing PSS^302^ mixture (State C) mostly exhibited the strongest affinity and most stable binding for DC-SIGN and Langerin. It is likely that the differences in activity between the *E*_S_ and *Z*_S_ conformations can be attributed to steric hindrance and the differences in the presentation of the sugar moieties in each case.

The *E*_S_ isomer is the most potent at inhibiting lectin binding to gp120, showing the lowest IC_50_ values for both the glucoside- and mannoside-containing molecular motors. The changes in the potency of each isomeric state highlight that the motor conformation alters the interaction efficiencies, presumably by changing the local availability of sugar moieties, illustrating that there is a future for fine-tuning biomolecular interactions with molecular motors.

Overall, the data show that the ability of motors to undergo precise conformational changes is a crucial part of optimizing their binding efficiency and potency. The work shows the potential of conformational control in photoadaptive molecular rotary motors for biomolecular adhesion. These results offer new opportunities for noninvasive, high-precision control in sensor development, biological recognition, and improving the effective delivery of therapeutics *via* targeting and light-activating functions.

## Supplementary Material



## Notes

Materials and methods, synthesis and characterization, Nile Red fluorescence assay, cryogenic transmission electron microscopy, dynamic light scattering, photoirradiation, surface plasmon resonance, and analytical data (PDF)

## Abbreviations

DC-SIGN: dendritic cell-specific intercellular adhesion molecule-3-grabbing nonintegrin
HIV: human immunodeficiency virus
gp120: glycoprotein 120 (HIV envelope glycoprotein)
IC_50_: half-maximal inhibitory concentration
Pox: poly­(2-oxazoline)
PEG: poly­(ethylene glycol)
CROP: cationic ring-opening polymerization
CuAAC: copper-catalyzed azide–alkyne cycloaddition
GPC: gel permeation chromatography
^1^H NMR: proton nuclear magnetic resonance spectroscopy
^13^C NMR: carbon-13 nuclear magnetic resonance spectroscopy
FT-IR: Fourier transform infrared spectroscopy
MALDI-ToF: matrix-assisted laser desorption/ionization time-of-flight mass spectrometry
SPR: surface plasmon resonance
HBS: HEPES-buffered saline
NRFA: Nile Red fluorescence assay
CAC: critical aggregation concentration
PSS: photostationary state
Man: mannoside
Glc: glucoside
THI: thermal helix inversion
MM: molecular motor
*k*_d obs_: observed dissociation rate constant
*k*_a obs_: observed association rate constant
*K*_d obs_: observed equilibrium dissociation constant
*R*_Max_: maximum binding response
