# Supplementary material for: Utilizing Light to Control Glycopolymer–DC-SIGN Interactions via Molecular Motors
Source: J Am Chem Soc. 2026 Jun 30;148(27):28075–86. doi: 10.1021/jacs.5c20077 (PMC13383628; doi:10.1021/jacs.5c20077)
Supplement: Supplementary file 1 [file ja5c20077_si_001.pdf]

ELECTRONIC SUPPORTING INFORMATION

Utilizing Light to Control Glycopolymer–DC-SIGN Interactions *via* Molecular Motors

Caitlin L. A. Nutting,[a] Adrien Combe,[b] Gokhan Yilmaz,[a] Richard Napier,[c] Ben L. Feringa*[b] and C. Remzi Becer*[a]

[a] C. L. A. Nutting, Dr. G. Yilmaz, Prof. Dr. C. R. Becer, Department of Chemistry, University of Warwick, CV4 7AL, Coventry, United Kingdom

[b] Dr. A. Combe, Prof. Dr. B. L. Feringa, Centre for System Chemistry, Stratingh Institute for Chemistry, University of Groningen, Nijenborgh 3, 9747AG Groningen, The Netherlands

[c] Prof. Dr. R. Napier, School of Life Sciences, University of Warwick, CV4 7AL, Coventry, United Kingdom

* Corresponding authors

Email: b.l.feringa@rug.nl and Remzi.Becer@warwick.ac.uk

Table of contents

1. Synthesis and characterization 3

1.1. Materials and methods 3

1.2. Synthesis of poly(2-oxazoline)-derived precursors 4

1.3. Synthesis of molecular motor-based precursors 7

1.4. Synthesis of β-D-mannopyranoside-derived precursor 11

1.5. Synthesis of different glycopolymers 14

2. Nile Red fluorescence assay (NRFA) 21

3. Cryogenic transmission electron microscopy (Cryo-TEM) 22

4. Photoirradiation 23

4.1. General 23

4.2. Additional investigations on the rotation of the molecular motors 23

4.3. Eyring analysis 25

5. Surface Plasmon Resonance (SPR) 26

5.1. Lectin binding study 26

5.2. Competition assay 26

5.3. Mass transport limitation 27

5.4. Sensorgrams from lectin binding study 28

5.5. Sensorgrams from competition assay 32

6. Analytical data 34

6.1. Proton and carbon nuclear magnetic resonance spectroscopy (^{1}H NMR and ^{13}C NMR) 34

6.2. Gel permeation chromatography (GPC) analysis 52

6.3. Fourier-transform infrared (FT-IR) spectra 56

6.4. Matrix assisted laser deabsorption ionization - time of flight (MALDI-ToF) 61

7. References 63

1. Synthesis and characterization

1.1. Materials and methods

Chemicals: All commercially available reagents were purchased from *Sigma-Aldrich*, *Fischer*, *Fujifilm*, *ABCR* or *Merck*, and were used as received unless otherwise stated. Methyl *para*-toluenesulfonate was distilled and then stored under nitrogen. The human lectins DC-SIGN (~75 kDa) and Langerin (58.3 kDa) were purchased from *Sino Biological*. Glycoprotein gp120 (55 kDa) was purchased from *Sigma-Aldrich*.

Flash chromatography: Flash chromatography was carried out using *Merck* silica gel 60 (230–400 mesh ASTM).

Proton and carbon nuclear magnetic resonance (^{1}H and ^{13}C NMR) spectrometry: NMR spectra were recorded at 25 °C on Bruker Avance 600 (^{1}H: 600 MHz, ^{13}C: 151 MHz) at University of Groningen, and on Bruker Avance III AV 400MHz (^{1}H: 400 MHz, ^{13}C: 101 MHz) at University of Warwick. Before use, the deuterated solvent $CDCl_3$ was treated with anhydrous potassium carbonate and magnesium sulfate. Chemical shifts (δ) are expressed relative to the resonances of the residual non-deuterated solvent for ^{1}H ($CDCl_3$: δ = 7.26 ppm, DMSO-d_6: δ = 2.50 ppm, D_2O: δ = 4.79 ppm) and ^{13}C ($CDCl_3$: δ = 77.16 ppm, CD_3OD 1 vol.% in D_2O: δ = 49.50 ppm). Absolute values of the coupling constants are given in Hertz (Hz). Multiplicities are abbreviated as singlet (s), doublet (d), doublet of doublets (dd), doublet of doublets of doublets (ddd), triplet (t), doublet of triplets (dt), quadruplet (q), pentuplet (p), multiplet (m), broad singlet (br s), broad doublet (br d) and broad multiplet (br m). All ^{13}C NMR signals are considered as single sharp peaks, unless otherwise stated.

Fourier-transform infrared (FT–IR) spectroscopy: FT-IR spectra was carried out with a Spectrum Two FT-IR Spectrometer (PerkinElmer). Signal types are indicated as strong (s), medium (m), weak (w), broad medium (br m) and broad weak (br w).

High-resolution mass spectrometry (HRMS): HRMS measurements were performed on a LTQ Orbitrap XL spectrometer with electrospray ionization (ESI).

Gel permeation chromatography (GPC): GPC measurements in tetrahydrofuran (THF) were performed using HPLC grade THF containing 2% triethylamine and 0.1% butylated hydroxytoluene (BHT) as eluent on Agilent Technologies 1260 Infinity. The instrument was equipped with a refractive index (RI) and a 308 nm UV detector, a PLgel 5 mm guard column, and a PLgel 5 mm mixed D column (300 × 7.5 mm). Samples were run at 1 mL.min^{-1} at 40 °C. Poly(methyl methacrylate) (PMMA) standards (Agilent PMMA calibration kits M-M-10 and M-L-10) were used for the calibration. Samples ran in THF were made up to 1 mg.mL^{-1}, and then before injection (100 mL), the samples were filtered through a PTFE membrane with a 0.2 mL pore size. Experimental molar mass (M_n), weight-average molar mass (M_w), and polydispersity (*Đ*) values of synthesized polymers were determined by conventional calibration using Agilent GPC/SEC software. GPC measurements in *N*,*N*-dimethylformamide (DMF) were carried out on an Agilent 1260 Infinity II instrument with two Polargel M columns and a Polargel M guard column. The mobile phase was DMF stabilized with 0.1% w/V lithium bromide with a flow rate of 1 mL.min^{-1} at 50 °C and equipped with the following detectors: differential refractive index (DRI), viscometer (VS), light scattering (LS), and variable wavelength detector (MWD). Agilent PMMA Easivials were used to create a third order calibration curve. The calibrants cover a 535–538 g.mol^{-1} range. All glycopolymer

samples were made up to 1 mg.mL^{-1} using DMF and were passed through 0.2 mm nylon filters prior to GPC measurements. All samples were then analyzed using Agilent GPC/SEC software.

Matrix assisted laser desorption ionization – time of flight (MALDI–ToF) mass spectrometry: MALDI–ToF mass spectra were performed on a Bruker Daltonics Autoflex spectrometer equipped with a nitrogen laser at 337 nm with positive ion detection. Polymer samples were prepared as follows: solutions in THF of *trans*-2-[3-(4-tert-butylphenyl)-2-methyl-2-propenylidene]malononitrile (DCTB, ≥98%) as matrix (20 mg.mL^{-1}), sodium trifluoroacetate (NaTFA) as cationization agent (10 mg.mL^{-1}), and polymer sample (5 mg.mL^{-1}) were mixed in a ratio of 5:2:5 and then spotted onto the target (0.5 mL). Spectra were recorded in reflective mode, and the mass spectrometer was calibrated with a PMMA standard up to 3 kDa.

1.2. Synthesis of poly(2-oxazoline)-derived precursors

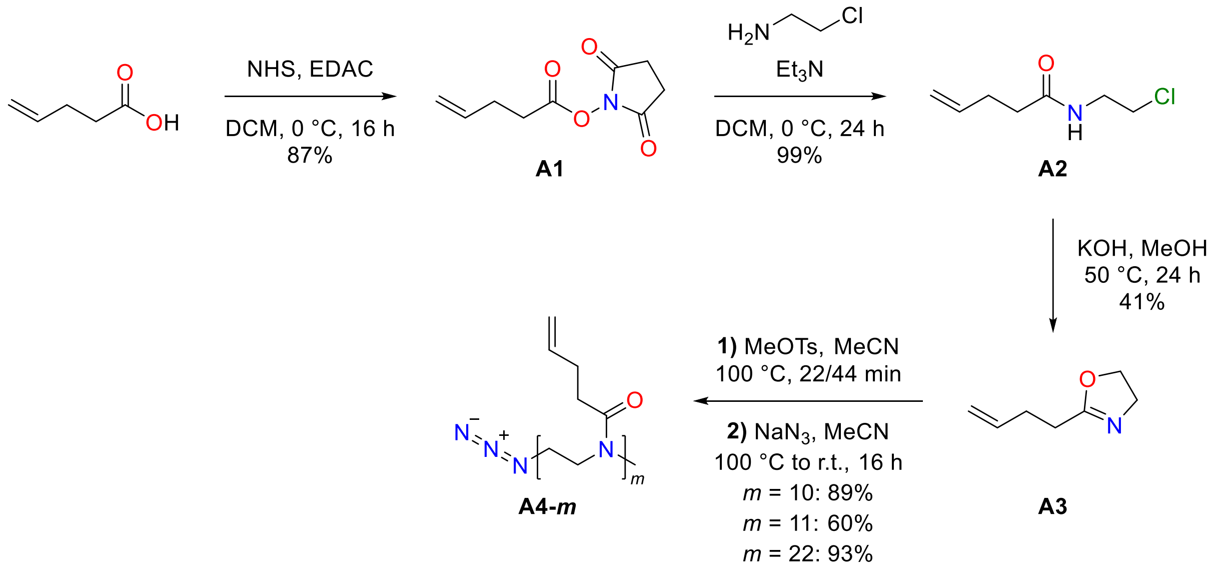

Scheme S1. Schematic illustration of the synthetic route of the precursors **A4-*m*** (*m* = 10, 11 or 22).

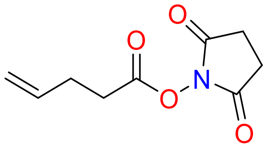

2,5-Dioxopyrrolidin-1-yl pent-4-enoate (**A1**)

4-pentenoic acid (38.5 g, 385 mmol, 1.0 eq.) and *N*-hydroxysuccinimide (70.8 g, 615 mmol, 1.6 eq.) were dissolved in dichloromethane (200 mL, 2 M), and the solution cooled to 0 °C. Then, ethyl-dimethylaminopropyl carbodiimide (EDAC) (88.5 g, 462 mmol, 1.2 eq.) was slowly added, and the mixture was stirred at 0 °C overnight. The resulting organic solution was washed with water (6 × 200 mL) and brine (2 × 200 mL), dried with anhydrous magnesium sulfate, and concentrated under reduced pressure to yield **A1** as a yellow oil (65.8 g, 334 mmol, 87%). ^{1}H NMR (400 MHz, $CDCl_3$): δ = 5.84–5.71 (m, 1H), 5.05 (d, *J* = 17.1 Hz, 1H), 4.99 (d, *J* = 10.3 Hz, 1H), 2.73 (s, 4H), 2.63 (t, *J* = 7.4 Hz, 2H), 2.39 (q, *J* = 7.2 Hz, 2H) ppm. Assignments correspond to the literature.[1]

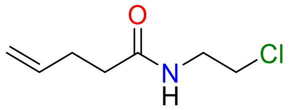

<u>*N*-(2-Chloroethyl)pent-4-enamide (**A2**)</u>

A1 (46.4 g, 235 mmol, 1.0 eq.) and 2-chloroethylamine (27.1 g, 235 mmol, 1.0 eq.) were dissolved in anhydrous dichloromethane (150 mL, 1.5 M), and the resulting solution was cooled down to 0 °C. Then, triethylamine (82 mL, 589 mmol, 2.5 eq.) was added dropwise, and the mixture was stirred at 0 °C overnight. The organic solution was washed with water (6 × 200 mL) and brine (200 mL), dried with anhydrous magnesium sulfate, and concentrated under reduced pressure to yield amide **A2** as a yellow oil (37.6 g, 233 mmol, 99%). ^{1}H NMR (400 MHz, $CDCl_3$): δ = 5.68–5.53 (m, 1H), 4.90–4.81 (m, 1H), 4.81–4.75 (m, 1H), 4.28 (br s, 1H), 4.05 (dt, *J* = 9.5, 0.4 Hz, 2H), 3.59 (dt, *J* = 9.7, 0.5 Hz, 2H), 2.17–2.15 (m, 4H) ppm. Assignments correspond to the literature.[1]

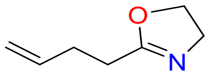

<u>2-(3-Butenyl)-2-oxazoline (**A3**)</u>

A2 (29.3 g, 181 mmol, 1.0 eq.) was dissolved in methanol (100 mL, 1.75 M), and then in a separate flask potassium hydroxide (10.2 g, 181 mmol, 1.3 eq.) was dissolved methanol (135 mL, 1.25 M). This solution was added dropwise to **A2**, and the resulting mixture was heated to 50 °C and stirred for 24h. The reactional solution was cooled down to room temperature, before being filtered to remove the salt precipitate. The resulting filtrate was concentrated under reduced pressure to afford the crude product as a yellow oil. The compound was purified by two distillations. The first distillation was carried out under vacuum, and the distillate was obtained at 30 °C. Subsequently, this distillate was stirred with calcium hydride for 24 h. The product was subjected to distillation a second time under vacuum, and the distillate collected into a dried nitrogen sparged Schlenk flask to yield **A3** as a colorless oil (12.0 g, 74.2 mmol, 41%). ^{1}H NMR (400 MHz, $CDCl_3$): δ = 6.02–5.65 (m, 1H), 5.07 (d, *J* = 17.5 Hz, 1H), 5.04 (d, *J* = 10.5 Hz, 1H), 4.23 (t, *J* = 9.5 Hz, 2H), 3.82 (t, *J* = 9.5 Hz, 2H), 2.46–2.31 (m, 4H) ppm; ^{13}C NMR (101 MHz, $CDCl_3$): δ = 167.7, 136.8, 115.3, 67.1, 54.3, 29.8, 27.3 ppm. Assignments correspond to the literature.[1]

<u>General procedure A:</u> Cationic ring-opening polymerization of oxazoline derivative.

Under a nitrogen atmosphere, in a dried microwave vial containing a stirring bar, oxazoline **A3** (10 eq.), anhydrous acetonitrile (4 M) and methyl *para*-toluenesulfonate (1.0 eq.) were combined and heated at 110 °C under vigorous stirring. After a pre-determined amount of time, the cap was removed before adding sodium azide (1.5 eq.) as a solid. The vial was resealed and left to cool down to room temperature and stirred overnight. Then, the resulting mixture was poured into cold diethyl ether, centrifuged and concentrated under reduced pressure, to obtain the polymers **A4-*m***.

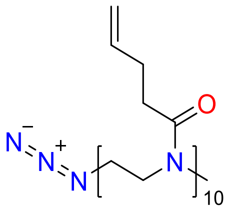

<u>α-Methyl-ω-azido-terminated poly(2-(3-butenyl)-2-oxazoline), DP = 10 (**A4-10**)</u>

The polymer **A4-10** was synthesized according to **general procedure A**, with precisely 22 min of stirring before addition of sodium azide, to afford the precursor as a white solid (0.891 g, 0.681 mmol, 89%). ^{1}H NMR (400 MHz, $CDCl_3$): δ = 5.90–5.72 (br m, 10H), 5.11–4.92 (br m, 20H), 3.60–3.31 (br m, 40H),

3.05–3.00 (br m, 3H), 2.52–2.25 (br m, 40H) ppm; IR (FT–IR): ν = 3080 (w, C–H_{alkene}), 2982, 2923 (m, C–H_{alkane}), 2104 (m, N=N_{azide}), 1630 (s, C=O_{amide}) cm^{-1}; GPC (THF): $t_{ret.}$ = 9.26 min, M_n = 1600 Da, M_w = 1900 Da, $Đ$ = 1.24.

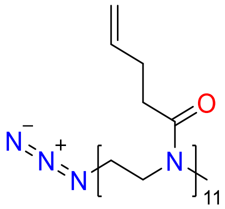

α-Methyl-ω-azido-terminated poly(2-(3-butenyl)-2-oxazoline), DP = 11 (**A4-11**)

The polymer **A4-11** was synthesized according to **general procedure A**, with precisely 22 min of stirring before addition of sodium azide, to afford the precursor as a white solid (0.660 g, 0.460 mmol, 44%). ^{1}H NMR (400 MHz, $CDCl_3$): δ = 5.90–5.72 (br m, 11H), 5.11–4.92 (br m, 22H), 3.60–3.31 (br m, 44H), 3.05–3.00 (br m, 3H), 2.52–2.25 (br m, 44H) ppm; IR (FT–IR): ν = 3079 (w, C–H_{alkene}), 2982, 2923 (m, C–H_{alkane}), 2104 (m, N=N_{azide}), 1633 (s, C=O_{amide}) cm^{-1}; GPC (THF): $t_{ret.}$ = 9.08 min, M_n = 2000 Da, M_w = 2500 Da, $Đ$ = 1.24; MALDI–ToF (ESI pos.): calculated from $C_{78}H_{125}N_{14}O_{11}$ ($[M + H]^+$): 1433.96, found 1434.08.

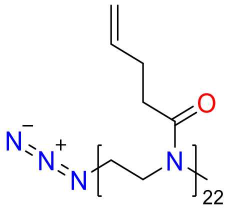

α-Methyl-ω-azido-terminated poly(2-(3-buteneyl)-2-oxazoline), DP = 22 (**A4-22**)

The polymer **A4-22** was synthesized according to **general procedure A**, with precisely 44 min of stirring before addition of sodium azide, to afford the precursor as a white solid (0.737 g, 0.262 mmol, 89%). ^{1}H NMR (400 MHz, $CDCl_3$): δ = 5.90–5.72 (br m, 22H), 5.11–4.92 (br m, 44H), 3.60–3.31 (br m, 88H), 3.05–3.00 (br m, 3H), 2.52–2.25 (br m, 88H) ppm; IR (FT–IR): ν = 3079 (w, C–H_{alkene}), 2982, 2923 (m, C–H_{alkane}), 2104 (m, N=N_{azide}), 1633 (s, C=O_{amide}) cm^{-1}; GPC (THF): $t_{ret.}$ = 8.74 min, M_n = 3100 Da, M_w = 4000 Da, $Đ$ = 1.28; MALDI–TOF (ESI pos.): calculated from $C_{155}H_{246}N_{25}O_{22}$ ($[M + H]^+$): 2809.89, found 2810.11.

1.3. Synthesis of molecular motor-based precursors

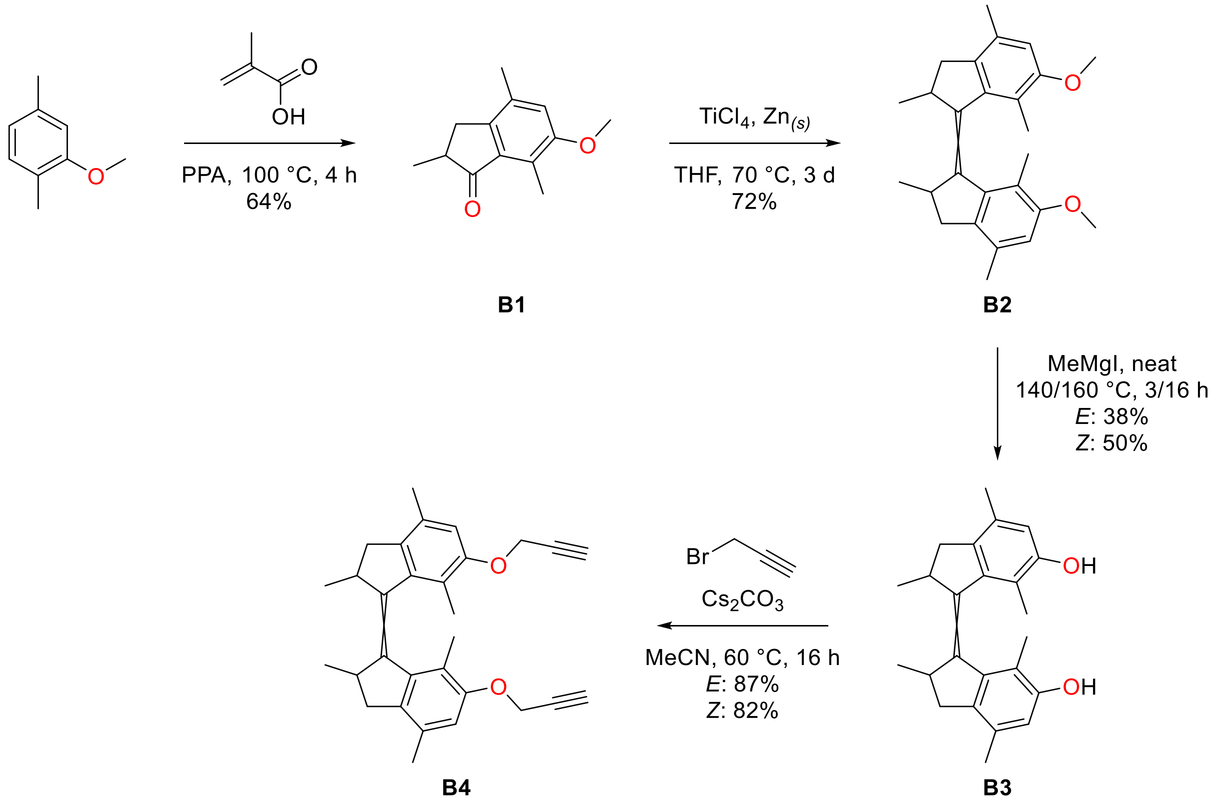

Scheme S2. Schematic illustration of the synthetic route of the precursor **B4**.

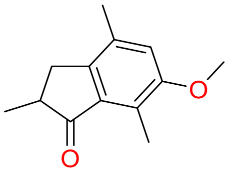

6-Methoxy-2,4,7-trimethyl-2,3-dihydro-1*H*-inden-1-one (**B1**)

2,4-dimethylanisole (25.0 mL, 175 mmol, 1.0 eq.), methacrylic acid (37.1 mL, 438 mmol, 2.5 eq.) and polyphosphoric acid (105% H_3PO_4 basis, 200 mL, 1 M) were combined, and the resulting mixture was agitated by mechanical stirring at 100 °C during 4 h. Then, the solution was cooled down to room temperature before adding ice (100 g) and mechanically stirred overnight. Dichloromethane (250 mL) was then added before filtering the polymeric impurities under vacuum. The resulting product was washed with dichloromethane (2 x 200 mL) which was then washed with a saturated aqueous solution of potassium carbonate (3 x 100 mL) and brine (100 mL). The organic layers were collected, dried with anhydrous magnesium sulfate, and concentrated under reduced pressure to afford the crude product as a brown solid. The compound was then recrystallized from *n*-heptane to yield **B1** as off-white crystals (22.9 g, 112 mmol, 64%). ^{1}H NMR (600 MHz, $CDCl_3$): δ = 6.92 (s, 1H), 3.84 (s, 3H), 3.17 (dd, *J* = 8.0, 16.7 Hz, 1H), 2.67 (p, *J* = 8.2 Hz, 1H), 2.50 (s, 3H), 2.47 (dd, *J* = 4.1, 16.6 Hz, 1H), 2.30 (s, 3H), 1.29 (d, *J* = 7.4 Hz, 3H) ppm. Assignments correspond to the literature.[2]

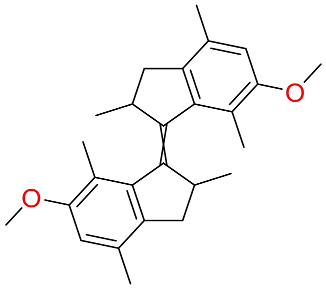

6,6'-Dimethoxy-2,2',4,4',7,7'-hexamethyl-2,2',3,3'-tetrahydro-1,1'-biindenylidene (**B2**)

Under a nitrogen atmosphere, zinc powder (25.6 g, 392 mmol, 8.0 eq.) was placed in anhydrous tetrahydrofuran (50 mL, 1 M) before cooling the mixture to 0 °C. Then, titanium tetrachloride (21.5 mL, 196 mmol, 4.0 eq.) was added dropwise and the resulting mixture was stirred at 70 °C for 2 h. The reaction mixture was cooled down again at 0 °C and a solution of **B1** (4 M in THF, 10.0 g, 48.9 mmol, 1.0 eq.) was added. The solution was heated again at 70 °C and stirred for 3 d. The mixture was cooled down to room temperature followed by filtration *via* celite plug. The crude product was washed with dichloromethane (100 mL) and then filtered. To the filtrate, a saturated aqueous solution of sodium bicarbonate (100 mL) was added and the mixture then washed with an aqueous solution of sodium carbonate (2 × 100 mL). From the aqueous layer, the compound was extracted again with dichloromethane (2 x 100 mL). The combined organic phase was washed with brine (100 mL), dried with anhydrous magnesium sulfate, and concentrated under reduced pressure to afford the crude product as a brown solid. The compound was subjected to flash chromatography (silica, *n*-pentane:ethyl acetate 19:1 V:V) to afford a pure mixture of diastereomers as a white solid (6.64 g, 17.6 mmol, 72%) with a *E*:*Z* ratio of 58:42. The molecular motor *(E)*-**B2** was recrystallized from ethanol to yield white crystals, and the filtrate was concentrated under reduced pressure to obtain an off-white solid as *Z*-enriched mixture of diastereomers (*E*:*Z* ratio of 12:88). ^{1}H NMR (600 MHz, $CDCl_3$): [*(E)*-**B2**] δ = 6.56 (s, 2H), 3.86 (s, 6H), 2.90 (p, *J* = 8.1 Hz, 2H), 2.60 (dd, *J* = 5.7, 14.1 Hz, 2H), 2.30 (s, 6H), 2.20 (s, 6H), 2.16 (d, *J* = 14.1 Hz, 2H), 1.09 (d, *J* = 6.5 Hz, 6H) ppm, [*(Z)*-**B2**] δ = 6.53 (s, 2H), 3.78 (s, 6H), 3.33 (p, *J* = 8.2 Hz, 2H), 3.05 (dd, *J* = 6.1, 14.6 Hz, 2H), 2.38 (d, *J* = 14.2 Hz, 2H), 2.26 (s, 6H), 1.37 (s, 6H), 1.07 (d, *J* = 6.8 Hz, 6H) ppm. Assignments correspond to the literature.[3]

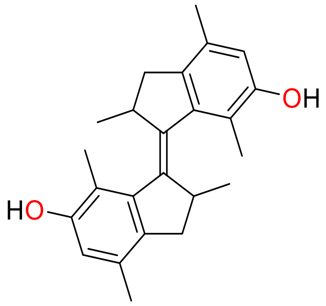

(E)-2,2',4,4',7,7'-Hexamethyl-2,2',3,3'-tetrahydro-[1,1'-biindenylidene]-6,6'-diol (*(E)*-**B3**)

Under a nitrogen atmosphere, in a dried reaction vial containing diastereomerically pure molecular motor *(E)*-**B3** (300 mg, 0.796 mmol, 1.0 eq.) and a stirring bar, a solution of methylmagnesium iodide (3.0 M in diethyl ether, 2.66 mL, 7.96 mmol, 10 eq.) was added and then heated to 100 °C and stirred for 2 h to remove the diethyl ether. Once the solvent was removed the flask was resealed and the resulting residue was heated to 140 °C for 3 h. The reaction mixture was cooled to room temperature, and ice (30 g) was carefully poured inside the vial. The resulting residue was diluted with a saturated aqueous solution of ammonium chloride (30 mL), and the product was extracted with ethyl acetate (3 x 30 mL). The combined organic layers were washed with brine (1 × 30 mL), dried with anhydrous

sodium sulfate, and concentrated under reduced pressure to obtain the crude product as a beige solid. The compound was purified by flash chromatography (silica, *n*-pentane:ethyl acetate 9:1 V:V) to afford the bis-phenol molecular motor *(E)*-**B3** as an off-white solid (106 mg, 0.302 mmol, 38%). ^{1}H NMR (600 MHz, DMSO-d_6): δ = 8.94 (s, 2H), 6.50 (s, 2H), 2.75 (p, *J* = 6.2 Hz, 2H), 2.43 (dd, *J* = 5.7, 14.1 Hz, 2H), 2.15 (s, 6H), 2.11 (d, *J* = 14.1 Hz, 2H), 2.05 (s, 6H), 1.02 (d, *J* = 6.3 Hz, 6H) ppm. Assignments correspond to literature.[4]

*Notes: If you heat above 140 °C, (E)-**B3** will isomerize to the more thermodynamically stable (Z)-**B3**. This compound in solution is sensitive to ambient air and light exposure. In the solid state, the compound is inert but should be stored in the dark. Therefore, it is advised to use it immediately in the next step after isolation.*

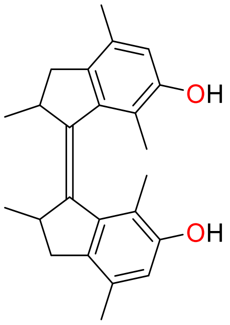

(Z)-2,2',4,4',7,7'-Hexamethyl-2,2',3,3'-tetrahydro-[1,1'-biindenylidene]-6,6'-diol (*(Z)*-**B3**)

Under a nitrogen atmosphere, in a dried reaction vial containing a *Z*-enriched mixture of diastereomers of molecular motor **B3** (300 mg, 0.796 mmol, 1.0 eq.) and a stirring bar, a solution of methylmagnesium iodide (3.0 M in diethylether, 2.66 mL, 7.96 mmol, 10 eq.) and heated to 100 °C for 2 h to remove the diethyl ether. Once the solvent was removed, the vial was sealed and the mixture heated 160 °C overnight. The reaction mixture was cooled to room temperature and ice (30 g) was carefully poured inside the vial. The resulting residue was diluted with a saturated aqueous solution of ammonium chloride (30 mL), and the product was extracted with ethyl acetate (3 x 30 mL). The combined organic layers were washed with brine (30 mL), dried with anhydrous sodium sulfate, and concentrated under reduced pressure to obtain the crude product as a beige solid. The compound was purified by flash chromatography (silica, *n*-pentane:ethyl acetate 17:3 V:V) to afford the bis-phenol molecular motor *(E)*-**B3** as an off-white solid (138 mg, 0.398 mmol, 50%). ^{1}H NMR (600 MHz, DMSO-d_6): δ = 8.77 (s, 2H), 6.47 (s, 2H), 3.25 (p, *J* = 6.7 Hz, 2H), 2.93 (dd, *J* = 6.2, 14.4 Hz, 2H), 2.31 (d, *J* = 14.4 Hz, 2H), 2.12 (s, 6H), 1.25 (s, 6H), 1.00 (d, *J* = 6.6 Hz, 6H) ppm. Assignments correspond to literature.[4]

Notes: This compound in solution is sensitive to ambient air and light exposure. In the solid state, the compound is inert but should be stored in the dark. Therefore, it is advised to use it immediately in the next step after isolation.

General procedure B: Williamson aryl-alkyl etherification of molecular motor derivatives.

Under a nitrogen atmosphere, in a dried pressure vial equipped with a stirring bar, diastereomerically pure bis-phenol motor **B3** (1.0 eq.) and anhydrous cesium carbonate (6.0 eq.) were placed in anhydrous acetonitrile (0.1 M). Then, a solution of propargyl bromide (80 wt.% in toluene, 3.0 eq.) was added to the heterogeneous mixture before heating to 60 °C and stirred overnight. The resulting solution was cooled to room temperature, and the solvent removed under reduced pressure. To the residue, a saturated aqueous solution of sodium bicarbonate was added, and the product was extracted with ethyl acetate (x 3). The combined organic phase was washed with brine, dried with

anhydrous magnesium sulfate, and concentrated under reduced pressure to obtain the crude product. Then, the compound was purified by flash chromatography (silica, *n*-pentane:ethyl acetate 19:1 V:V).

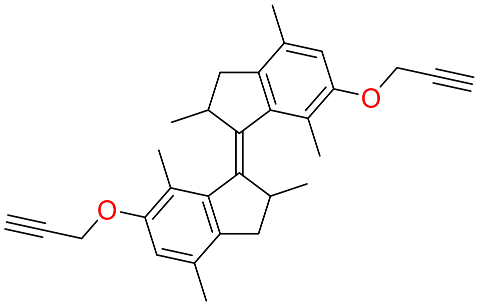

(E)-2,2',4,4',7,7'-Hexamethyl-6,6'-bis(prop-2-yn-1-yloxy)-2,2',3,3'-tetrahydro-1,1'-biindenylidene (*(E)*-**B4**)

The bis-propargyl molecular motor *(E)*-**B4** was synthesized from molecular motor *(E)*-**B3**, following the **general procedure B**, yielded as a white solid (87%). ^{1}H NMR (600 MHz, $CDCl_3$): δ = 6.67 (s, 2H), 4.73 (d, *J* = 2.4 Hz, 4H), 2.89 (p, *J* = 6.3 Hz, 2H), 2.60 (dd, *J* = 5.7, 14.2 Hz, 2H), 2.54 (t, *J* = 2.5 Hz, 2H), 2.33 (s, 6H), 2.21 (s, 6H), 2.17 (d, *J* = 14.2 Hz, 2H), 1.10 (d, *J* = 6.5 Hz, 6H) ppm; ^{13}C NMR (151 MHz, $CDCl_3$): δ = 155.4, 143.0, 142.0, 135.5, 131.7, 121.3, 112.0, 79.8, 75.3, 56.9, 42.5, 38.7, 19.5, 19.0, 16.5 ppm; IR (FT–IR): ν = 3309, 3290, 3271 (m, C–H_{alkyne}), 2119 (w, C≡C_{alkyne}), 1599 (m, C=$C_{aromatic}$) cm^{-1}; HRMS (ESI pos.): calculated for $C_{30}H_{33}O_2$ ([M + H]$^+$): 425.2475, found 425.2470.

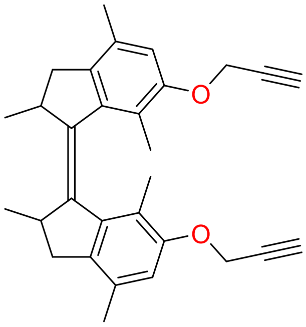

(Z)-2,2',4,4',7,7'-Hexamethyl-6,6'-bis(prop-2-yn-1-yloxy)-2,2',3,3'-tetrahydro-1,1'-biindenylidene (*(Z)*-**B4**)

The bis-propargyl molecular motor *(Z)*-**B4** was synthesized from molecular motor *(Z)*-**B3**, following the **general procedure B**, yielded as a white solid (82%). ^{1}H NMR (600 MHz, $CDCl_3$): δ = 6.65 (s, 2H), 4.69–4.60 (m, 4H), 3.33 (p, *J* = 6.7 Hz, 2H), 3.04 (dd, *J* = 6.3, 14.5 Hz, 2H), 2.50 (t, *J* = 2.4 Hz, 2H), 2.38 (d, *J* = 14.5 Hz, 2H), 2.26 (s, 6H), 1.41 (s, 6H), 1.07 (d, *J* = 6.8 Hz, 6H) ppm; ^{13}C NMR (151 MHz, $CDCl_3$): δ = 154.9, 142.5, 141.1, 137.5, 130.7, 123.2, 112.9, 79.7, 75.1, 57.1, 42.0, 38.3, 20.6, 19.0, 14.5 ppm; IR (FT–IR): ν = 3285 (m, C–H_{alkyne}), 2115 (w, C≡$C_{terminal\ alkyne}$), 1602 (m, C=$C_{aromatic}$) cm^{-1}; HRMS (ESI pos.): calculated for $C_{30}H_{33}O_2$ ([M + H]$^+$): 425.2475, found 425.2467.

1.4. Synthesis of β-D-mannopyranoside-derived precursor

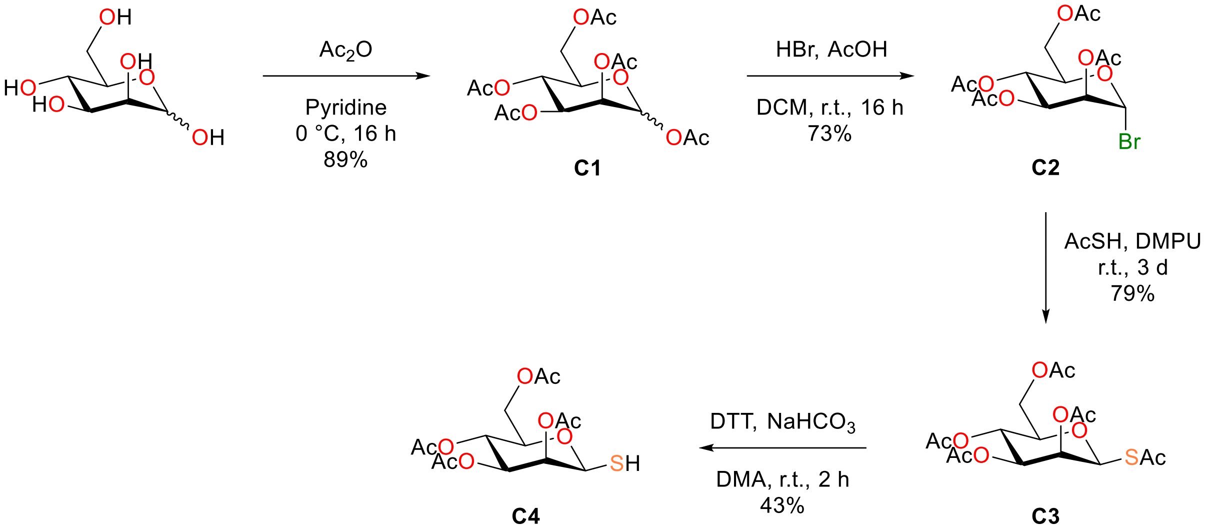

Scheme S3. Schematic illustration of the synthetic route of the precursor **C4**.

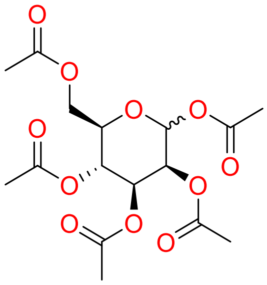

β-D-Mannopyranoside penta-*O*-acetate (**C1**)

D-*(+)*-mannopyranose (10.0 g, 56.0 mmol, 1.0 eq.) and pyridine (35.0 mL, 370 mmol, 7.0 eq.) were combined and placed at 0 °C. Then, acetic anhydride (27.0 mL, 330 mmol, 6.0 eq.) was added dropwise, and the mixture was stirred overnight. The resulting solution was diluted with ethyl acetate (100 mL) and then washed with an aqueous solution of hydrochloric acid (0.1 M, 3 × 100 mL), an aqueous solution of copper(II) sulfate (1 M, 3 × 100 mL) and brine (100 mL). The organic layers were dried with anhydrous magnesium sulfate, filtered and concentrated under reduced pressure to yield mannopyranoside **C1** as a colorless oil (19.2 g, 49.8 mmol, 89%) with a α:β ratio of 77:23. ^{1}H NMR (400 MHz, $CDCl_3$): [α-**C1**] δ = 6.08 (d, *J* = 1.8 Hz, 1H), 5.37–5.31 (m, 2H), 5.26 (t, *J* = 2.2 Hz, 1H), 4.30–4.23 (dd, *J* = 4.8, 11.6 Hz, 1H), 4.13–4.07 (m, 2H), 2.17 (s, 3H), 2.16 (s, 3H), 2.09 (s, 3H), 2.05 (s, 3H), 2.00 (s, 3H) ppm; [β-**C1**] δ = 5.85 (s, 1H), 5.49–5.46 (m, 1H), 5.32–5.26 (m, 1H) 5.15–5.10 (m, 1H), 4.34–4.29 (m, 1H), 4.16–4.12 (m, 1H), 3.84–3.76 (m, 1H), 2.21 (s, 3H), 2.20 (s, 3H), 2.10 (s, 3H), 2.03 (s, 3H), 2.00 (s, 3H) ppm. Assignments correspond to literature.[5,6]

<u>Note:</u> The α:β ratio has been determined by integration of the anomeric proton in the ^{1}H NMR spectrum.

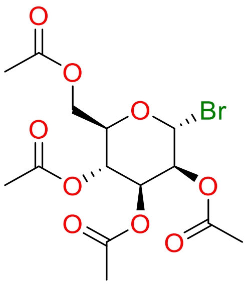

<u>1-Bromo-α-D-mannopyranoside tetra-*O*-acetate (**C2**)</u>

C1 (19.2 g, 49.2 mmol, 1.0 eq.) was dissolved in dichloromethane (100 mL, 0.5 M) and then, a solution of hydrobromic acid (33 wt.% in acetic acid, 111 mL, 589 mmol, 12 eq.) was added dropwise, and the mixture was stirred at room temperature overnight. The resulting organic solution was washed with a saturated aqueous solution of sodium bicarbonate (4 × 200 mL) and brine (100 mL), dried with anhydrous magnesium sulfate, and concentrated under reduced pressure to yield anomerically pure mannopyranoside **C2** as a yellow syrup (14.8 g, 36.0 mmol, 73%). ^{1}H NMR (400 MHz, $CDCl_3$): δ = 6.28 (s, 1H), 5.70 (dd, *J* = 3.1, 10.0 Hz, 1H), 5.43 (dd, *J* = 1.6, 3.6 Hz, 1H), 5.35 (p, *J* = 10.2 Hz, 1H), 5.30–5.22 (m, 1H), 4.32 (dd, *J* = 4.7, 12.4 Hz, 1H), 4.27–4.18 (m, 1H), 4.12 (dt, *J* = 3.5, 15.4 Hz, 1H), 2.16 (s, 3H), 2.09 (s, 3H), 2.06 (s, 3H), 1.99 (s, 3H) ppm. Assignments correspond to literature.[6]

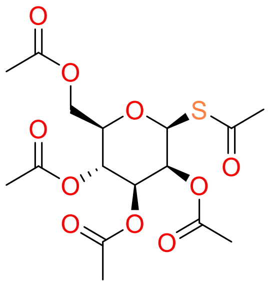

<u>1-Acetylthio-β-D-mannopyranoside tetra-*O*-acetate (**C3**)</u>

C2 (14.8 g, 36.0 mmol, 1.0 eq.) was dissolved in *N,N'*-dimethylpropyleneurea (67 mL, 0.5 M), and stirred at room temperature for 30 min. Then, potassium thioacetate (5.47 g, 39.6 mmol, 1.1 eq.) was added, and the mixture was stirred at room temperature for 3 d. The reacting mixture was then diluted in ethyl acetate (400 mL) and water (400 mL) which was then extracted with ethyl acetate (3 × 200 mL). The combined organic layers were washed with water (2 × 200 mL) and brine (100 mL), dried with anhydrous magnesium sulfate and concentrated under reduced pressure to afford the crude product as a red syrup. The residue was dissolved in the minimum amount of a mixture of dichloromethane and methanol (1:1 V:V) before being precipitated into cold petroleum ether (1.2 L). The compound was filtered and washed with cold petroleum ether to yield mannopyranoside **C3** as a light orange solid (11.2 g, 28.7 mmol, 79%). ^{1}H NMR (400 MHz, $CDCl_3$): δ = 5.48 (s, 1H), 5.47 (s, 1H), 5.25 (t, *J* = 9.8 Hz, 1H), 5.15–5.11 (m, 1H), 4.25 (dd, *J* = 5.3, 12.6 Hz, 1H), 4.11 (dd, *J* = 2.0, 12.3 Hz, 1H), 3.85–3.77 (m, 1H), 2.35 (s, 3H), 2.19 (s, 3H), 2.07 (s, 3H), 2.01 (s, 3H), 1.97 (s, 3H) ppm. Assignments correspond to literature.[5]

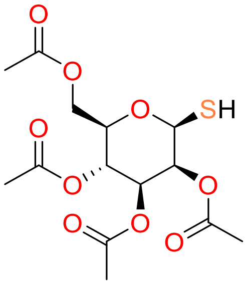

1-Thio-β-D-mannopyranoside tetra-*O*-acetate (**C4**)

C3 (1.00 g, 2.56 mmol, 1.0 eq.) was dissolved in anhydrous *N,N*-dimethylacetamide (15 mL, 0.15 M). Then, dithiothreitol (0.593 g, 3.84 mmol, 1.5 eq.) and anhydrous sodium bicarbonate (21.5 mg, 0.256 mmol, 0.1 eq.) were added, and the reaction mixture was stirred at room temperature for 2 h. The resulting mixture was poured into water (200 mL) and the product was extracted with toluene (3 × 100 mL). The combined organic layers were dried with anhydrous magnesium sulfate, and concentrated under reduced pressure. The residue was diluted again in toluene (100 mL) and concentrated under vacuum to fully remove the reaction solvent residue, affording the mannopyranoside **C4** as an off-white solid (0.388 g, 1.11 mmol, 43%). ^{1}H NMR (400 MHz, $CDCl_3$): δ = 5.44 (d, *J* = 3.4 Hz, 1H), 5.22 (t, *J* = 10.0 Hz, 1H), 5.08 (dd, *J* = 3.3, 10.2 Hz, 1H), 4.88 (d, *J* = 9.4 Hz, 1H), 4.24 (dd, *J* = 5.6, 12.5 Hz, 1H), 4.17–4.09 (br m, 1H), 3.71 (dt, *J* = 3.5, 9.6 Hz, 1H), 2.54 (d, *J* = 9.6 Hz, 1H), 2.23 (s, 3H), 2.09 (s, 3H), 2.04 (s, 3H), 2.00 (s, 3H) ppm; ^{13}C NMR (101 MHz, $CDCl_3$): δ = 170.8, 170.2, 170.2, 169.7, 129.2, 128.4, 125.4, 77.1, 76.5, 72.1, 71.7, 65.3, 62.8, 20.9, 20.8, 20.7, 20.7 ppm; IR (FT–IR): ν = 2923, 2852 (m, C–H$_{alkane}$), 2651 (br m, S–H$_{thiol}$), 1727 (s, C=O$_{acetate}$) cm^{-1}. Assignments correspond to literature.[7]

1.5. Synthesis of different glycopolymers

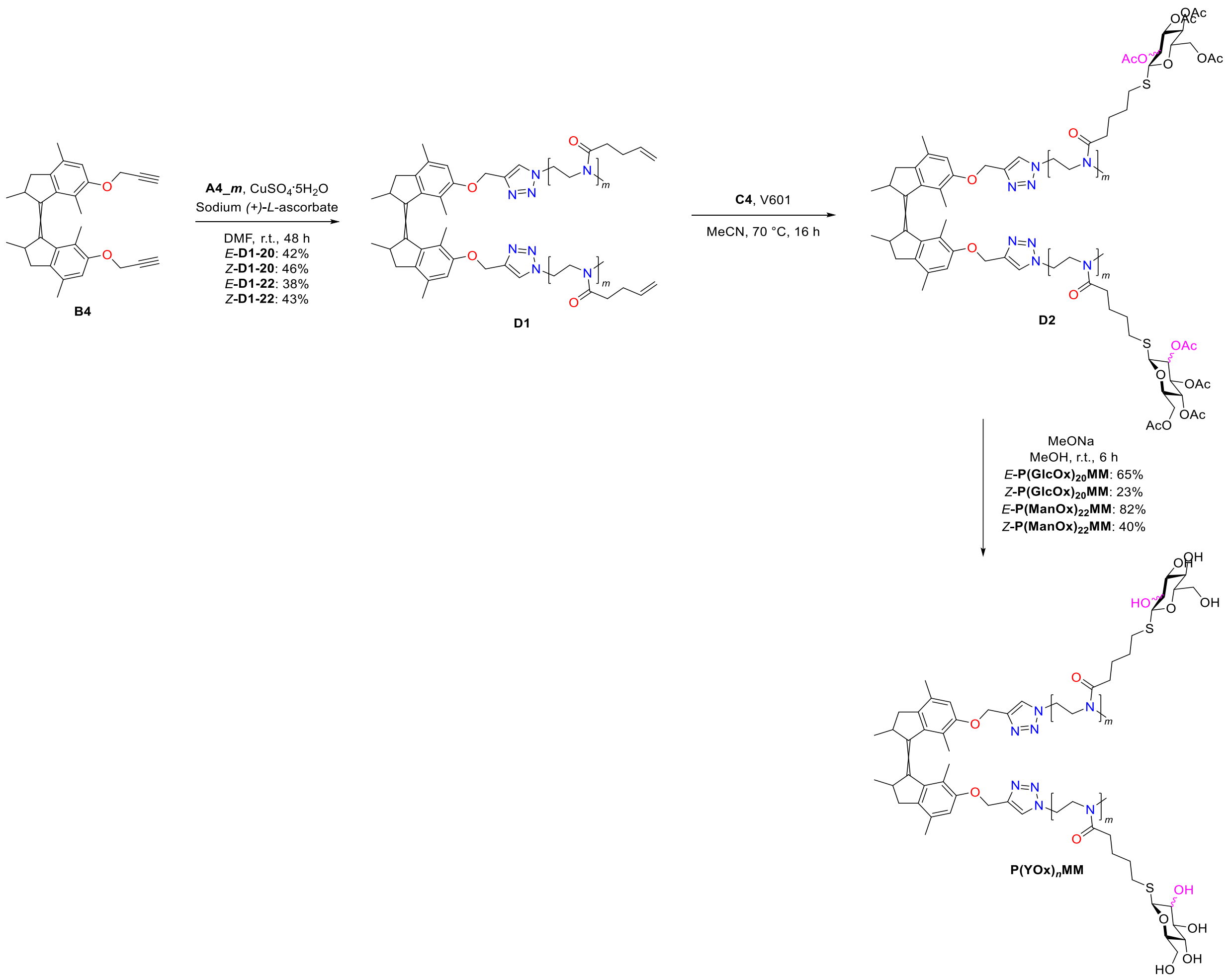

Scheme S4. Schematic illustration of the synthetic route of the glycopolymers-based molecular motors **P(GlcOx)$_{20}$MM** and **P(ManOx)$_{22}$MM**.

<u>General procedure C:</u> Copper-catalyzed azide-alkyne cycloaddition.

Under a nitrogen atmosphere, in a pressure vial covered with tin foil equipped with a stirring bar, diastereomerically pure bis-propargyl motor **B4** (1.0 eq.) and polymer **A4-*m*** (where *m* = 10,11 2.2 eq.) were dissolved in *N,N*-dimethylformamide (0.02 M). Then, an aqueous solution of copper(II) sulfate (0.0025 M, 0.25 eq.) and sodium L-*(+)*-ascorbate (0.005 M, 0.5 eq.) were added to the solution. The resulting mixture was stirred at room temperature for 48 h and then subsequently passed through a neutral alumina plug to afford the polymer-based molecular motor **D1-*n*** (where *n* = 20, 22).

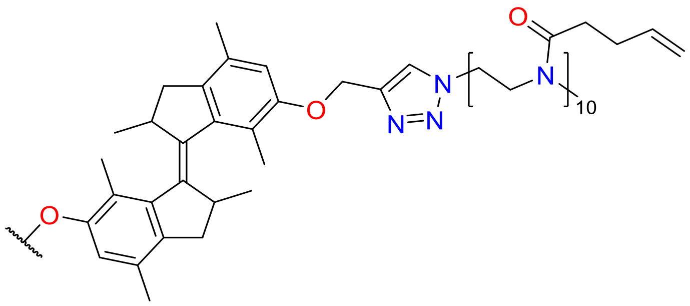

α-Methyl-ω-azido-terminated bis(poly(2-(3-buteneyl)-2-oxazoline))-substituted molecular *(E)*-motor, DP = 10 (*(E)*-**D1-20**)

The molecular motor *(E)*-**D1-20** was synthesized from molecular motor *(E)*-**B4** and polymer **A4-10**, following the **General procedure C**, yielded as a beige solid (42%). ^{1}H NMR (400 MHz, $CDCl_3$): δ = 6.59 (s, 2H), 5.74 (br s, 20H), 5.05–4.82 (br m, 40H), 4.28 (t, *J* = 7.0 Hz, 2H), 3.50–3.40 (br m, 80H), 2.49–2.15 (br m, 80H) ppm, 1.01 (d, *J* = 7.0 Hz, 6H) ppm; GPC (THF): $t_{ret.}$ = 8.76 min, M_n = 3000 Da, M_w = 3600 Da, $Đ$ = 1.31.

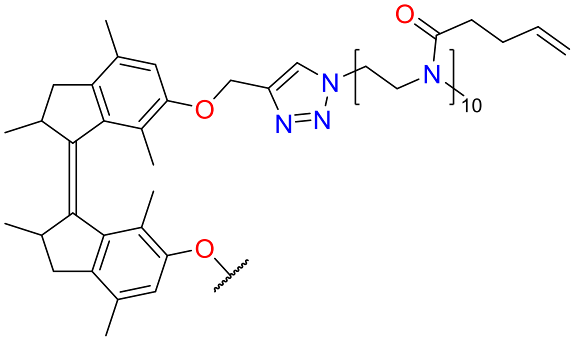

α-Methyl-ω-azido-terminated bis(poly(2-(3-buteneyl)-2-oxazoline))-substituted molecular *(Z)*-motor, DP = 10 (*(Z)*-**D1-20**)

The molecular motor *(Z)*-**D1-20** was synthesized from molecular motor *(Z)*-**B4** and polymer **A4-10**, following the **General procedure C**, yielded as a beige solid (46%). ^{1}H NMR (400 MHz, $CDCl_3$): δ = 6.65 (s, 2H), 5.82 (br s, 20H), 5.18–4.84 (br m, 40H), 2.54–2.14 (br s, 80H), 2.46–2.12 (br m, 80H), 1.08 (br s, 6H) ppm; GPC (THF): $t_{ret.}$ = 8.73 min, M_n = 3400 Da, M_w = 4100 Da, $Đ$ = 1.19.

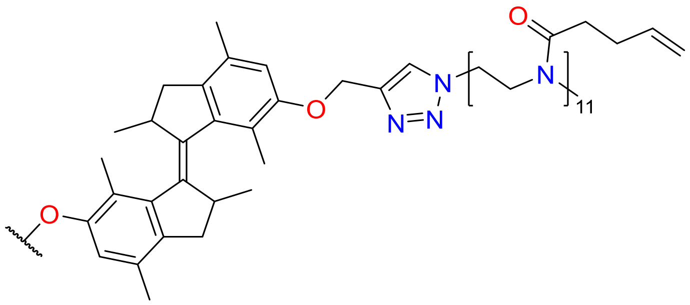

α-Methyl-ω-azido-terminated bis(poly(2-(3-buteneyl)-2-oxazoline))-substituted molecular *(E)*-motor, DP = 11 (*(E)*-**D1-22**)

The molecular motor *(E)*-**D1-22** was synthesized from molecular motor *(E)*-**B4** and polymer **A4-11**, following the **General procedure C**, yielded as a beige solid (38%). ^{1}H NMR (400 MHz, $CDCl_3$): δ = 6.59 (s, 2H) 5.74 (br s, 22H), 5.05–4.82 (br m, 44H), 4.28 (t, J = 7.0 Hz, 2H), 3.50–3.40 (br m, 88H), 2.49–2.15 (br m, 88H) ppm, 1.01 (d, J = 7.0 Hz, 6H) ppm; GPC (THF): $t_{ret.}$ = 8.76 min, M_n = 3500 Da, M_w = 4300 Da, $Đ$ = 1.22; MALDI–TOF (ESI pos.): calculated from $C_{186}H_{281}N_{28}O_{24}$ ([M + H]$^+$): 3291.16, found 3289.80.

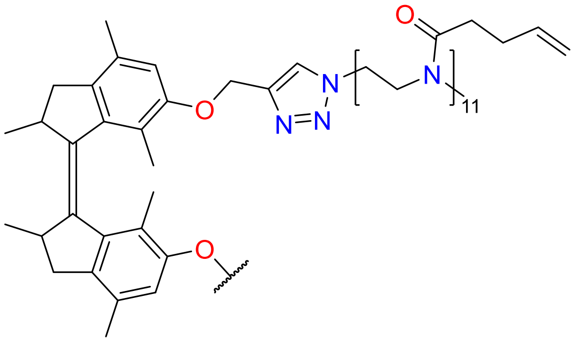

α-Methyl-ω-azido-terminated bis(poly(2-(3-buteneyl)-2-oxazoline))-substituted molecular *(Z)*-motor, DP = 11 (*(Z)*-**D1-22**)

The molecular motor *(Z)*-**D1-22** was synthesized from molecular motor *(Z)*-**B4** and polymer **A4-11**, following the **General procedure C**, yielded as a beige solid (43%). ^{1}H NMR (400 MHz, $CDCl_3$): δ = 6.59 (s, 2H), 5.74 (br s, 22H), 5.05–4.82 (br m, 44H), 4.28 (t, J = 7.0 Hz, 2H), 3.50–3.40 (br m, 88H), 2.49–2.15 (br m, 88H), 1.01 (d, J = 7.0 Hz, 6H) ppm; GPC (THF): $t_{ret.}$ = 8.74 min, M_n = 3580 Da, M_w = 4100 Da, *Đ* = 1.15; MALDI–TOF (ESI pos.): calculated from $C_{186}H_{281}N_{28}O_{24}$ ($[M + H]^+$): 3291.16, found 3289.78.

General procedure D: Thiol–ene coupling reaction.

Under a nitrogen atmosphere, in a pressure vial covered with tin foil and equipped with a stirring egg, poly(2-oxazoline)-derived molecular motor **D1-*n*** (*n* = 20 or 22) or polymer **A4-22** (1.0 eq.) was combined with 1-thio-β-D-pyranoside derivative (1.2 eq. per terminal alkene on the polymer backbone) and dimethyl 2,2'-azobis(2-methylpropionate) (V601) (0.25 eq. per terminal alkene on the polymeric backbone) in anhydrous tetrahydrofuran (0.02 M). The resulting mixture was degassed with nitrogen, before being heated at 70 °C and stirred overnight. The solution was then dried under reduced pressure to afford molecular motor **D2-Y** or **P(Ac$_4$YOx)$_{22}$** (Y = Glc or Man). The product was used in the next step without further purification.

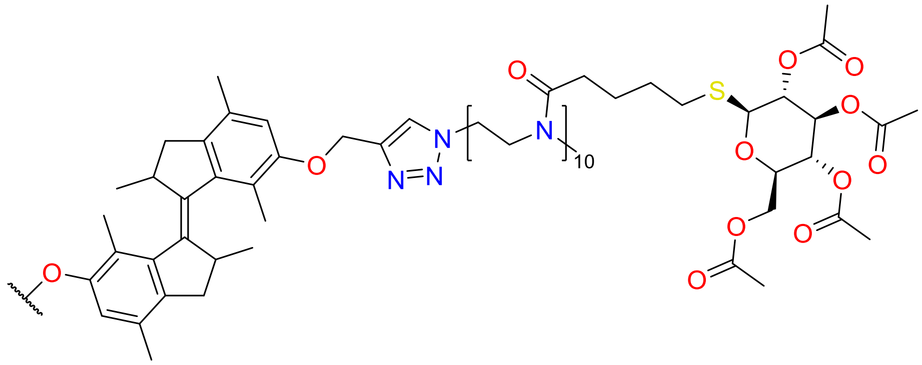

α-Methyl-terminated Bis(1-poly(2-(4-(*S*-per-*O*-acetyl-β-D-glucopyranosyl)butyl)-2-oxazoline)$_{10}$-1,2,3-triazol-4-yl)methoxy)-substituted molecular *(E)*-motor (*(E)*-**D2-Glc**)

The molecular motor *(E)*-**D2-Glc** was synthesized from molecular motor *(E)*-**D1-20** and 1-thio-β-D-glucopyranoside tetra-*O*-acetate, following the **General procedure D**, yielded as a cream solid. GPC (THF): $t_{ret.}$ = 8.36 min, M_n = 5200 Da, M_w = 6400 Da, *Đ* = 1.24.

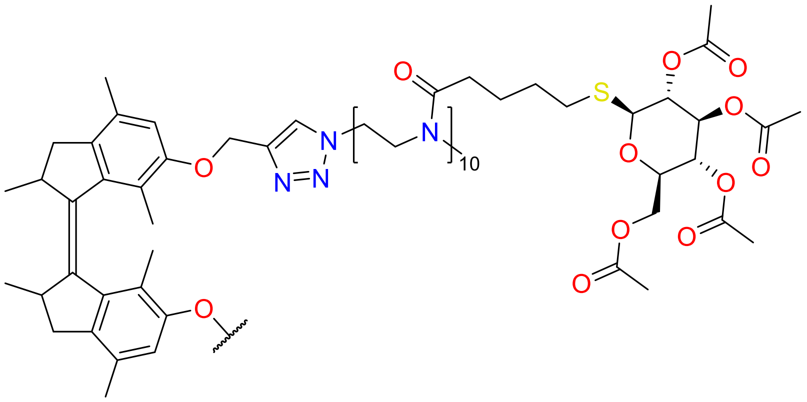

α-Methyl-terminated Bis(1-poly(2-(4-(*S*-per-*O*-acetyl-β-D-glucopyranosyl)butyl)-2-oxazoline)$_{10}$-1,2,3-triazol-4-yl)methoxy)-substituted molecular *(Z)*-motor (*(Z)*-**D2-Glc**)

The molecular motor *(Z)*-**D2-Glc** was synthesized from molecular motor *(Z)*-**D1-20** and 1-thio-β-D-glucopyranoside tetra-*O*-acetate, following the **General procedure D**, yielded as a cream solid. GPC (THF): $t_{ret.}$ = 8.33 min, M_n = 6400 Da, M_w = 7400 Da, $Đ$ = 1.16.

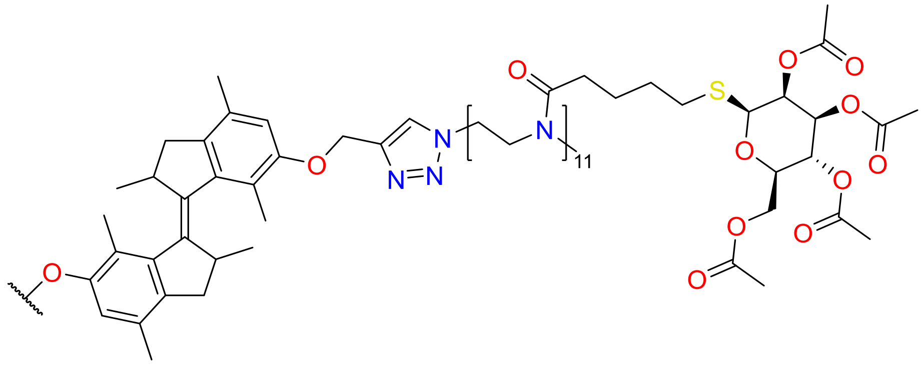

α-Methyl-terminated Bis(1-poly(2-(4-(*S*-per-*O*-acetyl-β-D-mannopyranosyl)butyl)-2-oxazoline)$_{11}$-1,2,3-triazol-4-yl)methoxy)-substituted molecular *(E)*-motor (*(E)*-**D2-Man**)

The molecular motor *(E)*-**D2-Man** was synthesized from molecular motor *(Z)*-**D1-22** and mannopyranoside **C4**, following the **General procedure D**, yielded as a cream solid. GPC (THF): $t_{ret.}$ = 8.28 min, M_n = 6300 Da, M_w = 8100 Da, $Đ$ = 1.29.

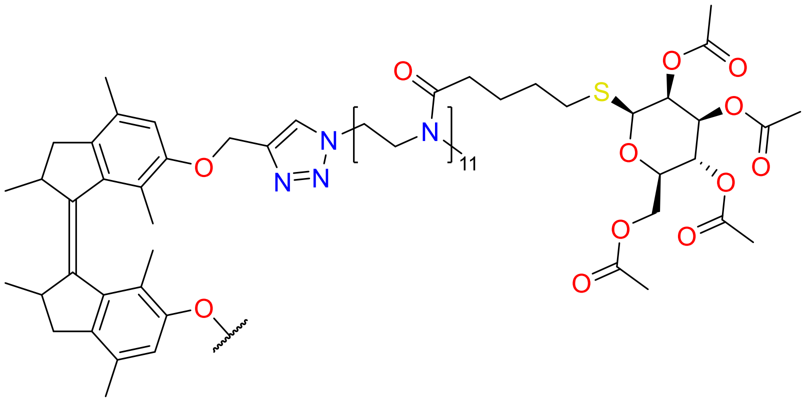

α-Methyl-terminated Bis(1-poly(2-(4-(*S*-per-*O*-acetyl-β-D-mannopyranosyl)butyl)-2-oxazoline)$_{11}$-1,2,3-triazol-4-yl)methoxy)-substituted molecular *(Z)*-motor (*(Z)*-**D2-Man**)

The molecular motor *(Z)*-**D2-Man** was synthesized from molecular motor *(Z)*-**D1-22** and mannopyranoside **C4**, following the **General procedure D**, yielded as a cream solid. GPC (THF): $t_{ret.}$ = 8.54 min, M_n = 4800 Da, M_w = 5700 Da, $Đ$ = 1.18.

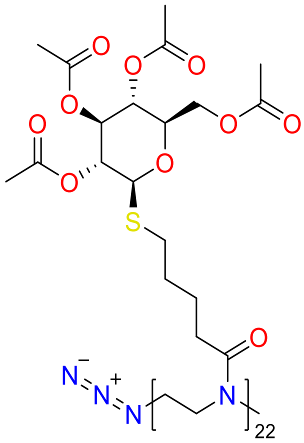

α-Methyl-ω-azido-terminated poly(2-(4-(*S*-per-*O*-acetyl-β-D-glucopyranosyl)butyl)-2-oxazoline), DP = 22

P(Ac$_4$GlcOx)$_{22}$

The glycopolymer **P(Ac$_4$GlcOx)$_{22}$** was synthesized from polymer **A4-22** and 1-thio-β-D-glucopyranoside tetra-*O*-acetate, following the **General procedure D**, yielded as a cream solid. GPC (THF): $t_{ret.}$ = 8.25 min, M_n = 7800 Da, M_w = 9100 Da, *Đ* = 1.16.

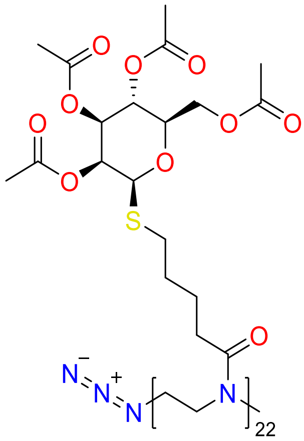

α-Methyl-ω-azido-terminated poly(2-(4-(*S*-per-*O*-acetyl-β-D-mannopyranosyl)butyl)-2-oxazoline), DP = 22

P(Ac$_4$ManOx)$_{22}$

The glycopolymer **P(Ac$_4$ManOx)$_{22}$** was synthesized from polymer **A4-22** and mannopyranoside **C4**, following the **General procedure D**, yielded as a cream solid. GPC (THF): $t_{ret.}$ = 8.64 min, M_n = 4600 Da, M_w = 5700 Da, *Đ* = 1.23.

General procedure E: Deacetylation of carbohydrate-derived moieties.

The acetylated glycopolymer derivatives **D2-Y** or **P(Ac$_4$YOx)$_{22}$** (Y = Glc or Man) (1.0 eq.) were dissolved in methanol (0.003 M). Then, a solution of sodium methoxide (2.0 M in methanol, 1.5 eq. per acetyl) was added dropwise, and the solution was stirred at room temperature for 6 h. The solvent was removed under reduced pressure, and the crude product was dissolved in water. Subsequently, the mixture was directly transferred to a dialysis tube (MWCO: 0.5 kDa) to be dialyzed against water for 3 d and was subsequently freeze-dried to yield the final compound **P(YOx)$_n$MM** or **P(YOx)$_{22}$** (Y = Glc or Man, *n* = 20 or 22, respectively).

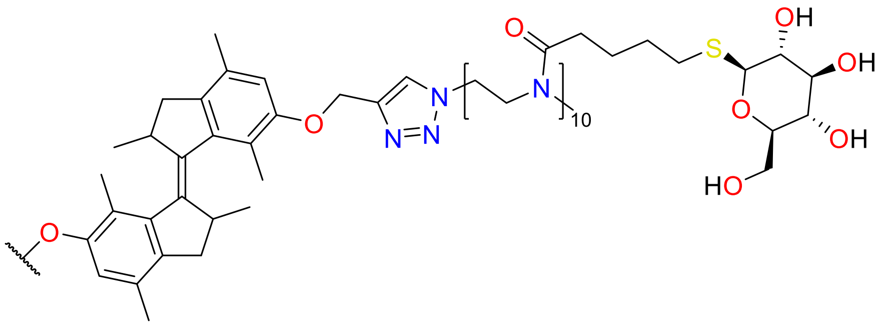

α-Methyl-terminated Bis(1-poly(2-(4-(*S*-β-D-glucopyranosyl)butyl)-2-oxazoline)$_{20}$-1,2,3-triazol-4-yl)methoxy)-substituted molecular *(E)*-motor (*(E)*-**P(GlcOx)$_{20}$MM**)

The molecular motor *(E)*-**P(GlcOx)$_{20}$MM** was synthesized from molecular motor *(E)*-**D2-Glc,** following the **General procedure E**, yielded as an off-white solid (65%). ^{1}H NMR (600 MHz, D_2O:CD_3OD 99:1 V:V): δ = 8.81 (br s, 2H), 6.78 (br s, 2H), 5.85 (br m, 6H), 5.06 (br m, 40H), 4.52 (br s, 20H), 3.95–3.21(br m, 240 H), 3.07 (br m, 4H), 2.94 (br s, 2H), 2.86–2.71(br m, 12H), 2.54–2.20 (br m, 40H), 1.66 (br s, 120H) ppm; ^{13}C NMR (151 MHz, D_2O:CD_3OD 99:1 V:V): δ = 138.6, 176.5, 117.2, 87.0, 86.9, 81.4, 78.8, 75.3, 73.9, 72.5, 71.2, 71.0, 62.5, 62.1, 33.5, 33.1, 31.1, 30.5, 30.2, 25.7, 25.4 ppm; IR (FT–IR): ν = 3345 (br m, O–H$_{glucoside}$), 2934, 2855 (br w, C–H$_{alkane}$), 1620 (br s, C=O$_{amide}$) cm^{-1}; GPC (DMF): $t_{ret.}$ = 15.78 min, M_n = 6900 Da, M_w = 11500 Da, $Đ$ = 1.37.

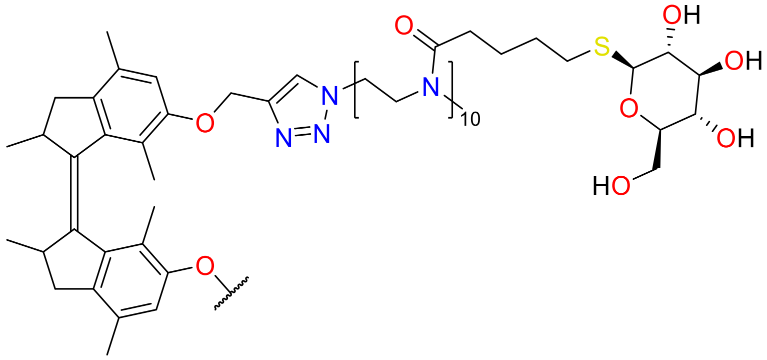

α-Methyl-terminated Bis(1-poly(2-(4-(*S*-β-D-glucopyranosyl)butyl)-2-oxazoline)$_{20}$-1,2,3-triazol-4-yl)methoxy)-substituted molecular *(Z)*-motor (*(Z)*-**P(GlcOx)$_{20}$MM**)

The molecular motor *(Z)*-**P(GlcOx)$_{20}$MM** was synthesized from molecular motor *(Z)*-**D2-Glc,** following the **General procedure E**, yielded as a beige solid (82%). ^{1}H NMR (600 MHz, D_2O:CD_3OD 99:1 V:V): δ = 8.11 (br s, 2H), 6.78 (br s, 2H), 5.85 (br m, 6H), 5.06 (br m, 40H), 4.52 (br s, 20H), 3.95–3.21 (br m, 240H), 3.07 (br m, 4H), 2.94 (br s, 2H), 2.86–2.71(br m, 12H), 2.54–2.20 (br m, 40H), 1.66 (br s, 120H) ppm; ^{13}C NMR (151 MHz, D_2O:CD_3OD 99:1 V:V): δ = 176.5, 138.6, 117.2, 87.0, 86.9, 81.4, 78.8, 75.3, 73.9, 72.5 71.2, 71.0, 62.5, 62.1, 33.5, 33.1, 31.1, 30.5, 30.2, 25.7, 35.4 ppm; IR (FT–IR): ν = 3360 (br m, O–H$_{glucoside}$), 2927, 2863 (br w, C–H$_{alkane}$), 1621 (br s, C=O$_{amide}$) cm^{-1}; GPC (DMF): $t_{ret.}$ = 15.80 min, M_n = 6800 Da, M_w = 9600 Da, $Đ$ = 1.41.

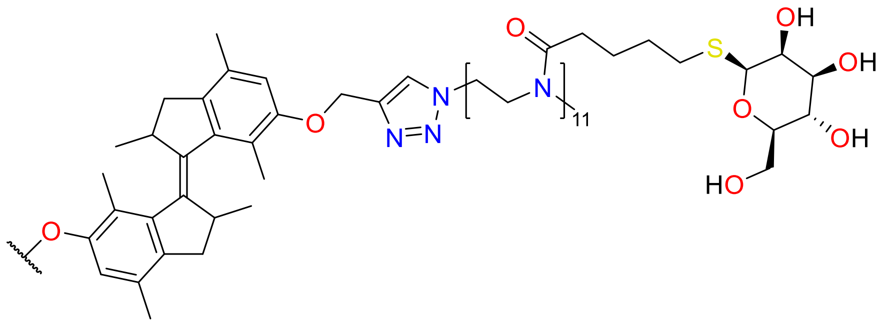

α-Methyl-terminated Bis(1-poly(2-(4-(*S*-β-D-mannopyranosyl)butyl)-2-oxazoline)$_{22}$-1,2,3-triazol-4-yl)methoxy)-substituted molecular *(E)*-motor (*(E)*-**P(ManOx)$_{22}$MM**)

The molecular motor *(E)*-**P(ManOx)$_{20}$MM** was synthesized from molecular motor *(E)*-**D2-Man**, following the **General procedure E**, yielded as a beige solid (23%). ^{1}H NMR (600 MHz, D_2O:CD_3OD 9:1 V:V): δ = 8.08 (br s, 2H), 6.74 (br s, 2H), 5.81 (br m, 6H), 5.00 (br m, 44H), 4.52 (br s, 22H), 3.96–3.26 (br m, 264 H), 3.08 (br m, 4H), 2.99 (br s, 2H), 2.79–2.70 (br m, 12H), 2.47–2.08 (br m, 44H), 1.60 (br s, 132H) ppm; ^{13}C NMR (151 MHz, D_2O:CD_3OD 9:1 V:V): δ = 177.2, 138.7, 117.2, 100.3, 86.4, 86.0, 82.7, 81.9, 75.6, 74.8, 73.9, 73.5, 72.8, 70.0, 70.0, 68.6, 68.2, 62.5, 40.3, 32.6, 31.5, 31.1, 30.4, 21.6, 17.7 ppm; IR (FT–IR): ν = 3381 (br m, O–H$_{mannoside}$), 2925, 2862 (br w, C–H$_{alkane}$), 1623 (br s, C=O$_{amide}$) cm^{-1}; GPC (DMF): t$_{ret.}$ = 15.93 min, M$_n$ = 6200 Da, M$_w$ = 7400 Da, Đ = 1.19.

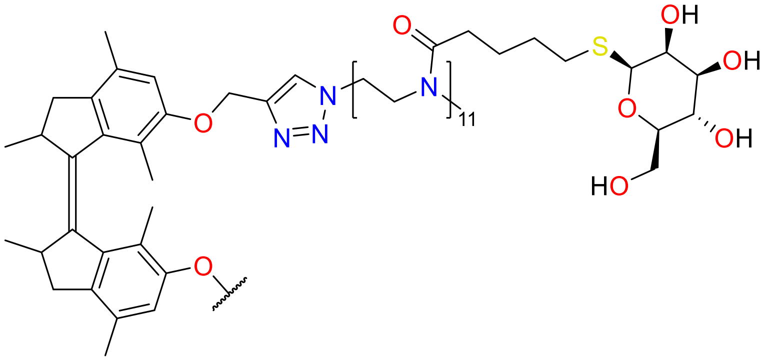

α-Methyl-terminated Bis(1-poly(2-(4-(*S*-β-D-mannopyranosyl)butyl)-2-oxazoline)$_{22}$-(1,2,3-triazol-4-yl)methoxy)-substituted molecular *(Z)*-motor (*(Z)*-**P(ManOx)$_{22}$MM**)

The molecular motor *(Z)*-**P(ManOx)$_{22}$MM** was synthesized from molecular motor *(Z)*-**D2-Man,** following the **General procedure E**, yielded as a beige solid (40%). ^{1}H NMR (600 MHz, D_2O:CD_3OD 9:1 V:V): δ = 8.02 (br s, 2H), 6.62 (br s, 2H), 5.82 (br m, 6H), 5.02 (br m, 44H), 3.02–3.30 (br m, 264H), 3.10 (br m, 4H), 3.07–2.98 (br m, 12H), 2.90 (br s, 2H), 2.86–2.71 (br m, 12H), 2.44–2.10 (br m, 44H), 1.60 (br s, 132H) ppm; ^{13}C NMR (151 MHz, D_2O:CD_3OD 99:1 V:V): δ = 176.5, 138.6, 117.2, 87.0, 86.9, 81.4, 78.8, 75.3, 73.9, 72.5 71.2, 71.0, 62.5, 62.1, 33.5, 33.1, 31.1, 30.5, 30.2, 25.7, 35.4 ppm; IR (FT–IR): ν = 3380 (br m, O–H$_{mannoside}$), 2925, 2860 (br w, C–H$_{alkane}$), 1621 (br s, C=O$_{amide}$) cm^{-1}; GPC (DMF): t$_{ret.}$ = 16.15 min, M$_n$ = 5100 Da, M$_w$ = 6100 Da, Đ = 1.20.

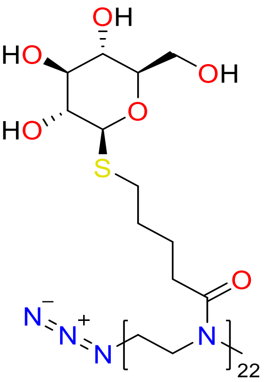

<u>α-Methyl-ω-azido-terminated poly(2-(4-(*S*-β-D-glucopyranosyl)butyl)-2-oxazoline), DP = 22 **P(GlcOx)$_{22}$**</u>

The glycopolymer **P(GlcOx)$_{22}$** was synthesized from polymer **P(Ac$_4$GlcOx)$_{22}$**, following the **General procedure E**, yielded as a white fluffy solid (54%). ^{1}H NMR (600 MHz, D_2O:CD_3OD 99:1 V:V): δ = 4.53 (d, *J* = 10.0 Hz, 22H), 3.90 (d, *J* =12.3 Hz, 22H), 3.71 (dd, *J* = 12.5, 5.5 Hz, 22H), 3.66–3.38 (m, 154 H), 3.32 (t, *J* = 9.5 Hz, 22H), 2.86–2.70 (m, 44H), 2.47–2.24 (m, 44H), 1.67 (br d, 88H) ppm; ^{13}C NMR (151 MHz, D_2O:CD_3OD 99:1 V:V): δ = 177.4, 86.9, 81.4, 78.8, 73.9, 71.1, 69.3, 62.5, 33.5, 33.4, 31.1, 30.6, 25.7, 25.4 ppm; IR (FT-IR): ν = 3312 (br m, O–$H_{glucoside}$), 2929, 2864 (br w, C–H_{alkane}), 2166 (w, N=N_{azide}), 1609 (br s, C=O_{amide}) cm^{-1}; GPC (DMF): $t_{ret.}$ = 15.57 min, M_n = 9300 Da, M_w = 11600 Da, *Đ* = 1.24.

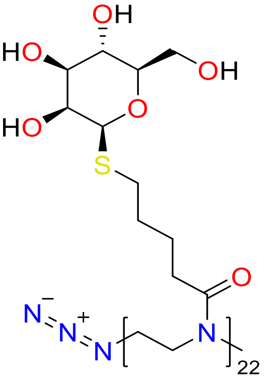

<u>α-Methyl-ω-azido-terminated poly(2-(4-(*S*-β-D-mannopyranosyl)butyl)-2-oxazoline), DP = 22 **P(ManOx)$_{22}$**</u>

The glycopolymer **P(ManOx)$_{22}$** was synthesized from polymer **P(Ac$_4$ManOx)$_{22}$**, following the **General procedure E**, yielded as a white fluffy solid (68%). ^{1}H NMR (400 MHz, D_2O:CD_3OD 9:1 V:V): δ = 5.26 (s, 22H), 4.53 (d, *J* = 10.0 Hz, 22H), 3.98 (d, *J* =12.3 Hz, 22H), 3.84 (dd, *J* = 12.5, 5.5 Hz, 22H), 3.79–3.40 (m, 154 H), 3.35 (br t, *J* = 9.5 Hz, 22H), 2.79–2.58 (m, 44H), 2.50–2.18 (m, 44H), 1.63 (br s, 88H) ppm; ^{13}C NMR (101 MHz, D_2O:CD_3OD 9:1 V:V): δ = 177.0, 138.6, 117.2, 86.4, 86.0, 82.8, 75.3, 74.8, 74.3, 73.8, 73.7, 73.5, 73.1, 72.8, 72.5, 68.6, 68.2, 68.1, 62.7, 62.5, 40.3, 33.5, 33.2, 32.2, 31.9, 30.6, 30.5, 30.1, 25.7, 25.5 ppm; IR (FT-IR): ν = 3319 (br m, O–$H_{glucoside}$), 2925, 2865 (br w, C–H_{alkane}), 2165 (w, N=N_{azide}), 1612 (br s, C=O_{amide}) cm^{-1}; GPC (DMF): $t_{ret.}$ = 15.85 min, M_n = 6700 Da, M_w = 8000 Da, *Đ* = 1.18.

2. Nile Red fluorescence assay (NRFA)

The water-solubility and potential self-assembly properties of the analytes **P(GlcOx)$_{20}$MM**, **P(ManOx)$_{22}$MM**, **P(GlcOx)$_{22}$** and **P(ManOx)$_{22}$** were analyzed by incorporation of the hydrophobic solvatochromic probe Nile Red (9-diethylamino-5-benzo[*α*]phenoxazinone), which shows a blueshift of the emission wavelength when it is encapsulated in hydrophobic environments. Freshly prepared

Nile Red ethanol solution (250 μM) was diluted into analyte sample solutions in HEPES-buffered saline (HBS) solution (HEPES 10 mM, NaCl 150 mM, $CaCl_2$ 5 mM, pH 7.40) to a final concentration of 250 nM. Each sample contains 0.1 vol.% of ethanol, limiting the effect of organic solvent on the assemblies. Subsequently, the mixture solutions containing analyte and Nile Red were excited at 550 nm, and the fluorescence spectra with a wavelength range of 575–725 nm were recorded by using a JASCO FP6200 fluorometer. The blueshifts were calculated by subtracting the maximum emission wavelength of Nile Red in the same HBS solution from the maximum emission wavelength of the sample. Each sample was ran once. Then, the different critical aggregation concentrations (CAC) are determined by plotting the obtained blueshifts values against the different concentrations using a *Logistic* function on a logarithmic scale of the concentrations of the analyte. The sigmoidal fitting has been performed using the software *Origin 2018*.

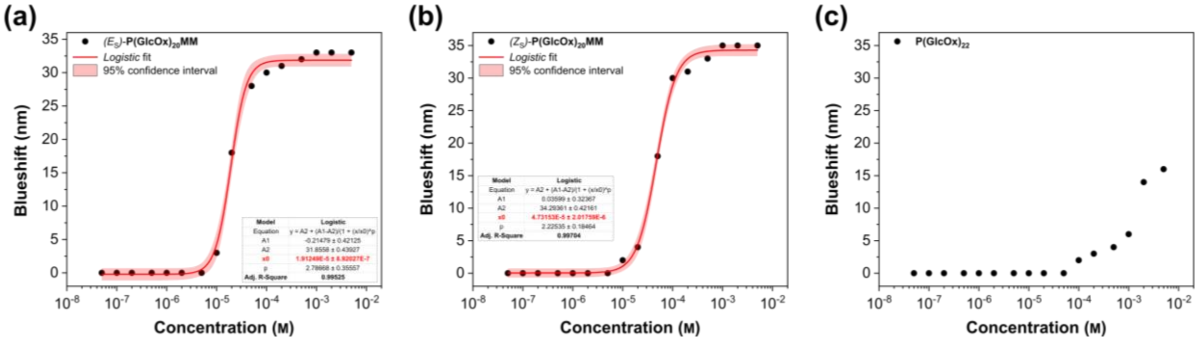

Figure S1. Nile Red Fluorescence Assay of **(a)** *(E_S)*-**P(GlcOx)$_{20}$MM**, **(b)** *(Z_S)*-**P(GlcOx)$_{20}$MM** and **(c) P(GlcOx)$_{22}$** in HBS solution (HEPES 10 mM, NaCl 150 mM, $CaCl_2$ 5 mM, pH 7.40), to determine their critical aggregation concentration (CAC).

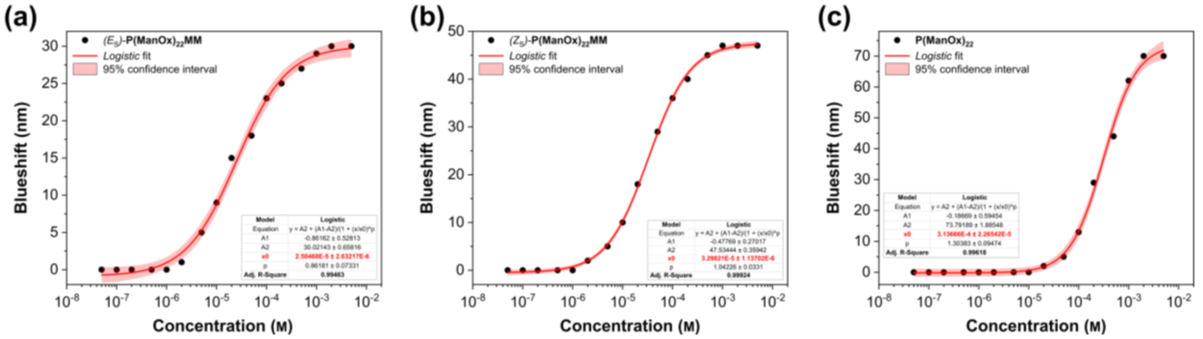

Figure S2. Nile Red Fluorescence Assay of **(a)** *(E_S)*-**P(ManOx)$_{22}$MM**, **(b)** *(Z_S)*-**P(ManOx)$_{22}$MM** and **(c) P(ManOx)$_{22}$** in HBS solution (HEPES 10 mM, NaCl 150 mM, $CaCl_2$ 5 mM, pH 7.40), to determine their critical aggregation concentration (CAC).

3. Cryogenic transmission electron microscopy (Cryo-TEM)

To observe the self-assembly structures by cryo-TEM, 2.5 μL of **P(GlcOx)$_{20}$MM** (10 mM), **P(ManOx)$_{22}$MM** (10 mM), **P(GlcOx)$_{22}$** (20 mM) and **P(ManOx)$_{22}$** (20 mM) solution in HBS solution (HEPES 10 mM, NaCl 150 mM, $CaCl_2$ 5 mM, pH 7.40) was placed on a glow-discharged holy carbon-coated grid. After blotting, the corresponding grid was rapidly frozen in liquid ethane and kept in liquid nitrogen until measurement. The grids were observed with a Gatan model 626 cryo-stage in a Tecnai T20 cryo-electron microscope operating at 200 keV. Cryo-TEM images were recorded under low-dose conditions on a slow-scan CCD camera.

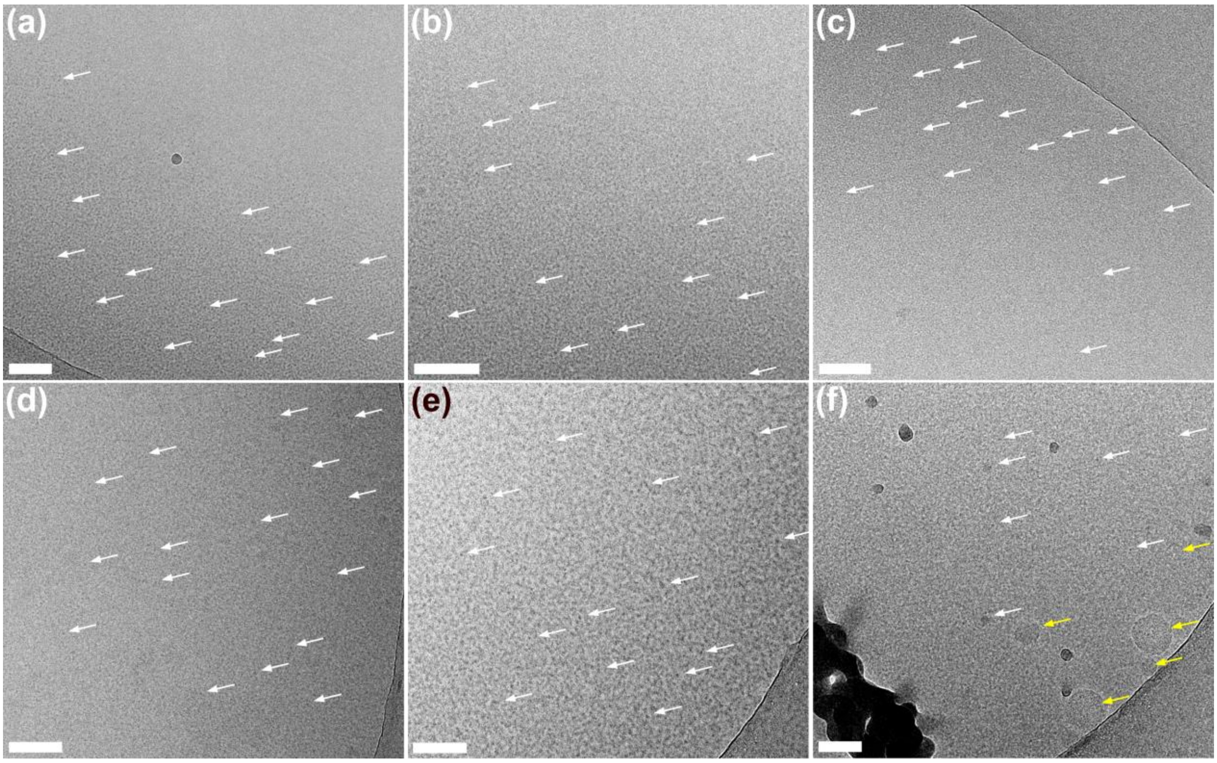

Figure S3. Cryo-TEM measurements of **(a)** *(E_S)*-**P(GlcOx)$_{20}$MM**, **(b)** *(Z_S)*-**P(GlcOx)$_{20}$MM**, **(c) P(GlcOx)$_{22}$**, **(d)** *(E_S)*-**P(ManOx)$_{22}$MM**, **(e)** *(Z_S)*-**P(ManOx)$_{22}$MM** and **(f) P(ManOx)$_{22}$**. Samples concentration is 10 mM for molecular motors, and 20 mM for control glycopolymers. Scale bars at 100 nm. Micelles are indicated by white arrows. Droplets are indicated by yellow arrows. Not all objects were indicated.

4. Photoirradiation

4.1. General

Ultraviolet-visible (UV-Vis) spectra were recorded on a Hewlett-Packard HP 8543 spectrometer in a quartz cuvette with 1 cm path length. Irradiations for UV-Vis measurements were carried out using two distinct LED light sources with a maximum emission of light at 365 nm (Thorlabs, M365FP1, 1.2 A, 9.8 mW) and 310 nm (Thorlabs, M310L1, 0.6 A, 38.5 mW). The thermal helix inversion (THI) studies were performed according to the previous work from our group.[9,10] Fittings have been done using the software *Origin 2018*, with the *DecExp1* function for time-dependent absorbance changes, and *Linear* function for Eyring analysis.

4.2. Additional investigations on the rotation of the molecular motors

The conversion from stable *(E_S)*-**P(YOx)$_n$MM** (Y = Glc or Man, n = 2 x m = 20 or 22, respectively) to the metastable *(Z_M)*-**P(YOx)$_n$MM** (Figure S4a) was carried out by irradiating a solution of *(E_S)*-**P(YOx)$_n$MM** at 310 nm until the photostationary state (PSS^{310} mixture) was reached (**Figure S4b, c**). During this process, two isosbestic points were observed at 267 and 335 nm for both motors, suggesting that the photochemical reaction does not occur obvious degradation of the molecular motor core. Interestingly, when the PSS^{310} mixture was irradiated again at 365 nm to revert to the stable *(E_S)*-**P(YOx)$_n$MM**, a different species formed (**Figure S4d, e**). While two clear isosbestic points were noted at 266/267 and 330 nm, these values differ slightly from those observed in its reverse

photoisomerization. Additionally, the UV-Vis spectrum of the newly formed PSS^{365} mixture showed subtle shape differences compared to the original *(E_S)*-**P(YOx)$_n$MM**, possibly indicating photodegradation.

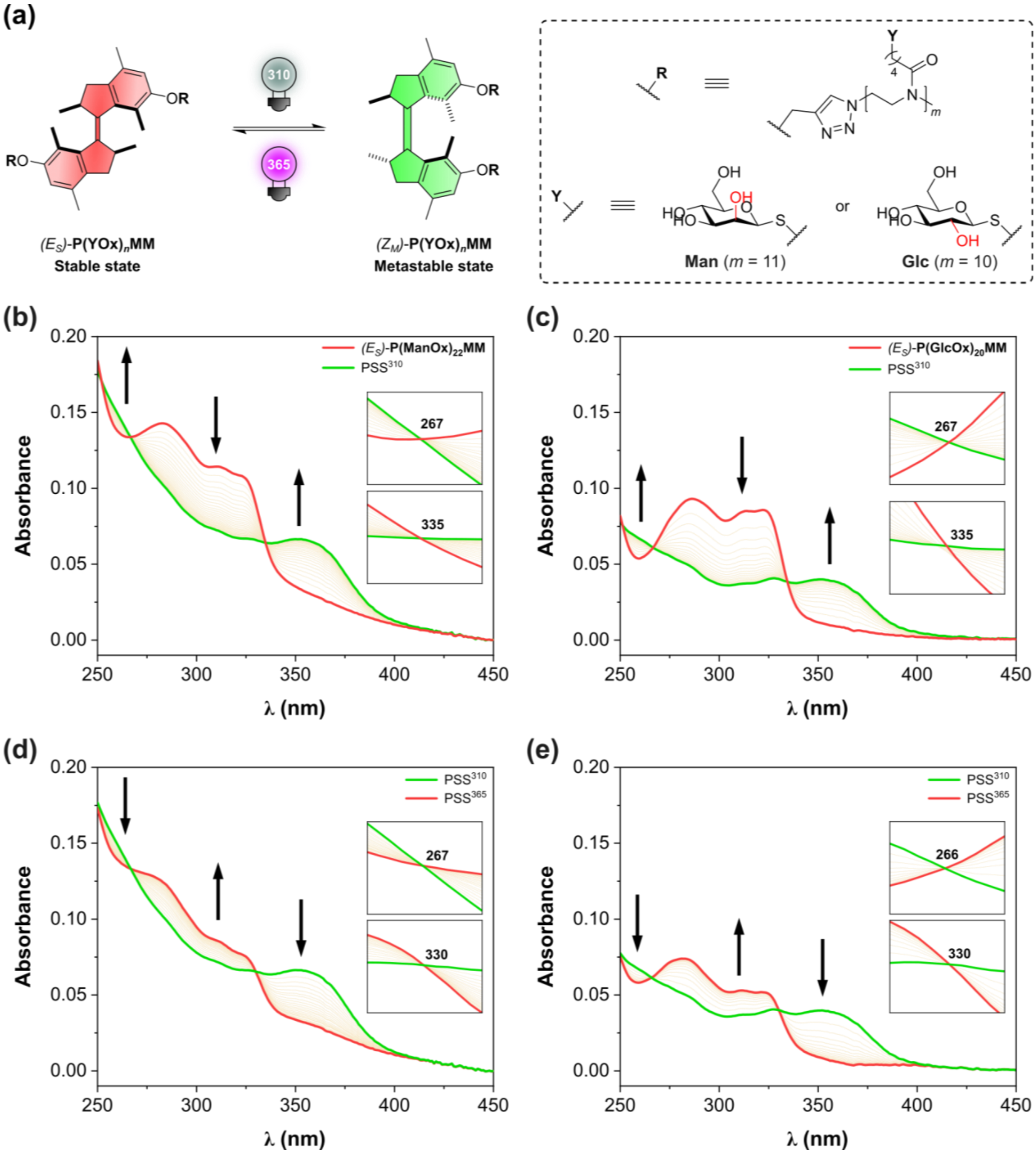

Figure S4. **(a)** Scheme of the photochemical isomerization from stable *(E_S)*-**P(YOx)$_n$MM** to metastable *(Z_M)*-**P(YOx)$_n$MM**. *n* corresponds to the number of **YOx** where *n* = 22 (for **Y** = **Man**) or *n* = 20 (for **Y** = **Glc**). All chiral centers are relative. **(b,c)** UV-Vis spectra of pure stable **(b)** *(E_S)*-**P(ManOx)$_{22}$MM** and **(c)** *(E_S)*-**P(GlcOx)$_{20}$MM** upon 310 nm light irradiation for 1 h converting to metastable *(Z_M)*-**P(ManOx)$_{22}$MM** and *(Z_M)*-**P(GlcOx)$_{20}$MM**, respectively, as PSS^{310} mixture (5 µM in HBS, below CAC, 20 °C). **(d,e)** UV-Vis spectra of previously produced PSS^{310} mixture of **(d) P(ManOx)$_{22}$MM** and **(e) P(GlcOx)$_{20}$MM** upon 365 nm light irradiation for 3 min converting to PSS^{365} mixture (5 µM in HBS, below CAC, 20 °C).

4.3. Eyring analysis

The stability of the metastable *(Z*$_M$*)*-**P(YOx)$_n$MM** (Y = Glc or Man, *n* = 2 x *m* = 20 or 22, respectively) in HBS solution (HEPES 10 mM, NaCl 150 mM, $CaCl_2$ 5 mM, pH 7.40) was investigated using Eyring analysis.[9,10] By this method, the activation parameters of the metastable *(Z*$_M$*)*-**P(YOx)$_n$MM** to stable *(Z*$_S$*)*-**P(YOx)$_n$MM** conversion (**Figure S5**) were obtained with the standard Gibbs energy activation ($\Delta^{\ddagger}G°$), and the different half-lives at 20 and 37 °C (**Figure S5, Table S1**).

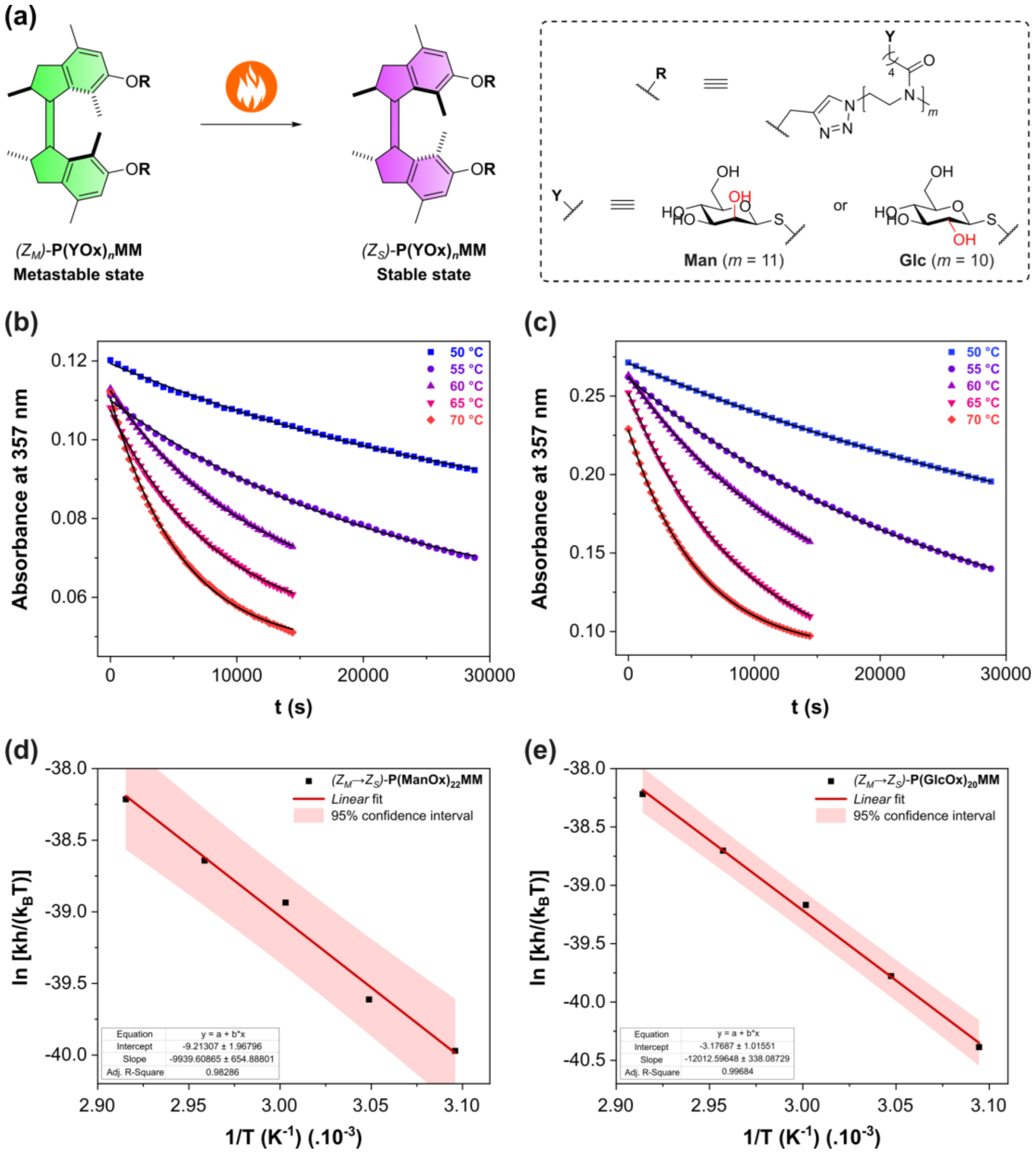

Figure S5. **(a)** Scheme of the thermal helix inversion (THI) process, converting the metastable *(Z*$_M$*)*-**P(YOx)$_n$MM** to *(Z*$_S$*)*-**P(YOx)$_n$MM**. *n* corresponds to the number of **YOx** where *n* = 22 (for **Y** = **Man**) or *n* = 20 (for **Y** = **Glc**). All chiral centers are relative. **(b,c)** Time-dependent absorbance at 357 nm during the THI process of **(b)** *(Z*$_M$*)*-**P(ManOx)$_{22}$MM** and **(c) P(GlcOx)$_{20}$MM** in HBS solution (HEPES 10 mM, NaCl 150 mM, $CaCl_2$ 5 mM, pH 7.40) at different temperatures (15 μM for **Y** = **Man** and 30 μM for **Y** = **Glc**, below CAC of *(Z*$_S$*)*-**P(YOx)$_n$MM**). **(d,e)** Eyring analysis of the THI process of metastable **(d)** *(Z*$_M$*)*-**P(ManOx)$_{22}$MM** and **(e)** *(Z*$_M$*)*-**P(GlcOx)$_{20}$MM** in HBS solution.

Table S1. Activation parameters and half-lives of metastable *(Z*$_M$*)*-**P(YOx)*n*MM** (Y = Glc or Man, *n* = 20 or 22, respectively) in HBS solution (HEPES 10 mM, NaCl 150 mM, $CaCl_2$ 5 mM, pH 7.40) from PSS[310] mixture during its thermal helix inversion (THI) process.

Z_M isomer	$\Delta^‡H°$ (kJ.mol^{-1})	$\Delta^‡S°$ (J.mol^{-1}.K^{-1})	$\Delta^‡G°_{293\,K}$ (kJ.mol^{-1})	$\Delta^‡G°_{310\,K}$ (kJ.mol^{-1})	$t_{1/2}$[a] (h)	$t_{1/2}$[b] (h)
**P(GlcOx)$_{20}$MM**	99.9	-26.4	107.6 ± 1.1	108.1 ± 0.7	476.7	47.4
**P(ManOx)$_{22}$MM**	83.2	-74.9	105.2 ± 2.1	106.5 ± 1.3	175.8	25.4

$t_{1/2}$ values calculated for [a] T = 293 K (20 °C), and [b] T = 310 K (37 °C).

5. Surface Plasmon Resonance (SPR)

5.1. Lectin binding study

Surface plasmon resonance (SPR) was used for interaction analysis for all lectins. The extent of interaction between the glycopolymers and lectins was analyzed on a BIAcore T200 system (Cytiva Life Sciences). The lectins (5 μg.mL^{-1}) were immobilized *via* a standard amino coupling protocol onto a CM5 sensor chip that was activated by flowing a 1:1 mixture of 0.1 M of *N*-hydroxysuccinimide and 0.05 M of *N*-ethyl-*N*′(dimethylaminopropyl)carbodiimide over the chip for 5 min at 25 °C at a flow rate of 10 μL.min^{-1} after the system equilibration with filtered HBS solution (HEPES 10 mM, NaCl 150 mM, $CaCl_2$ 5 mM, pH 7.40). Subsequently, channels 1 (blank), 2, 3 and 4 were blocked by flowing a solution of ethanolamine (1 M, pH 8.50) for 10 min at 10 μL.min^{-1} to block the remaining reactive groups on the channels. Sample solutions were prepared at varying concentrations (16.0–0.5 μM, below CAC) in HBS solution using a flow rate of 30 μL.min^{-1} to calculate the binding kinetics. Sensorgrams for each glycopolymer concentration were recorded with a 350 s injection of polymer solution (on period), followed by 200 s of buffer alone (off period). Regeneration of the sensor chip surfaces was performed using a different HBS solution (10 mM HEPES, 150 mM NaCl, 10 mM EDTA, 0.01% Tween20 surfactant, pH 7.40). Prior to evaluation, each complete sensorgram underwent a double-blank subtraction: one using the reference channel (channel 1) and one with a blank sample of just buffer and no analyte. Kinetic data was evaluated using a single set of sites (1:1 Langmuir binding model) in the BIA evaluation 3.1 software. The most concentrated sample of the *Z* stable state (State A) which was Irradiated before SPR measurements were carried out using handheld lamp with 365 nm for 1 h (State B) (UVP, UVLMS-38EL, 0.16 A, 8 W) and 302 nm for 2 hours (State C) (UVP, UVLMS-38EL, 0.16 A, 8 W) and then was heated for 18 h at 70 °C (State D). An example of the kinetic fits is shown in **Figure S13**, and the fitted data is included in **Table 1**.

5.2. Competition assay

SPR was used for interaction analysis for all competition assays. The extent of interaction between the glycopolymers and lectins was analyzed on a BIAcore T200 system (Cytiva Life Sciences). gp120 (5 μg.mL^{-1}) was immobilized *via* a standard amino coupling protocol onto a CM5 sensor chip that was activated by flowing a 1:1 mixture of 0.1 M *N*-hydroxysuccinimide and 0.05 M *N*-ethyl-*N*′(dimethylaminopropyl)carbodiimide over the chip for 5 min at 25 °C at a flow rate of 10 μL.min^{-1} after the system equilibration with filtered HBS solution (HEPES 10 mM, NaCl 150 mM, $CaCl_2$ 5 mM, pH 8.0). Subsequently, channels 1 (blank) and 2 were blocked by flowing a solution of ethanolamine (1 M, pH 8.50) for 10 min at 10 μL.min^{-1} to block the remaining reactive groups on the channels. Sample

solutions were prepared with a constant DC-SIGN concentration (40 nM) mixed with varying concentrations (800–50 nM) of polymer in HBS solution at 30 uL per minute to calculate the binding kinetics. Sensorgrams for each glycopolymer concentration were recorded with a 250 s injection of polymer solution (on period), followed by 300 s of buffer alone (off period). Regeneration of the sensor chip surfaces was performed using glycine (10 mM glycine, pH 2.5). Prior to evaluation, each complete sensorgram underwent a double-blank subtraction: one using the reference channel (channel 1) and one with a blank sample of just buffer and no analyte Kinetic data was evaluated using a single set of sites (1:1 Langmuir binding model) in the BIA evaluation 3.1 software. The most concentrated sample of the *Z* stable state (State A) which was irradiated before SPR measurements were carried out using handheld lamp with 365 nm for 1 h (State B) (UVP, UVLMS-38EL, 0.16 A, 8 W) and 302 nm for 2 hours (State C) (UVP, UVLMS-38EL, 0.16 A, 8 W) and then was heated for 18 h at 70 °C (State D).

5.3. Mass transport limitation

A mass transport limitation experiment was conducted to ensure the correct flow rate was selected. The rate of binding plateaus at 30 μL.min^{-1} indicates that the kinetic sensorgrams and results are not limited by mass transport.

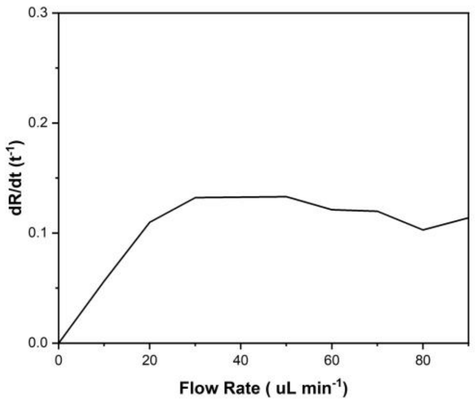

Figure S6. Mass transport limitation evaluation.

5.4. Sensorgrams from lectin binding study

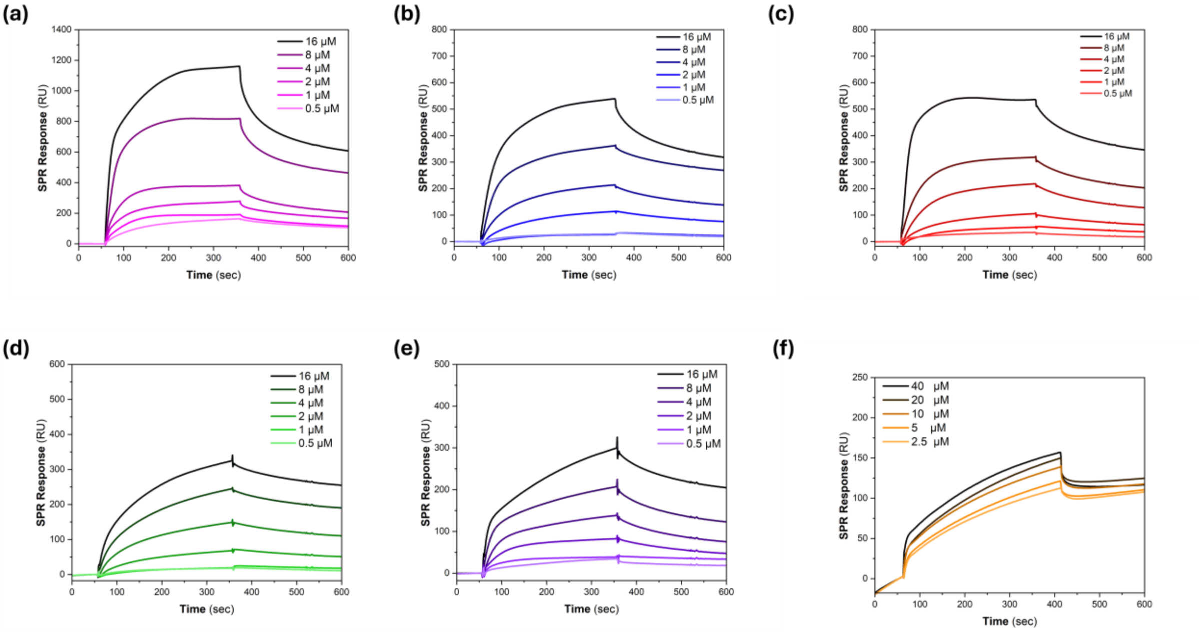

***Figure S7*. Sensorgrams depicting the association and dissociation states with DC-SIGN** of **(a)** *(E)*-**P(GlcOx)$_{20}$MM**, **(b)** *(Z)*-**P(GlcOx)$_{20}$MM** (State A), **(c)** PSS^{365} (State B), **(d)** PSS^{302} (State C), **(e)** after THI process from PSS^{302} (State D), and **(f)** the control glycopolymer **P(GlcOx)$_{22}$**.

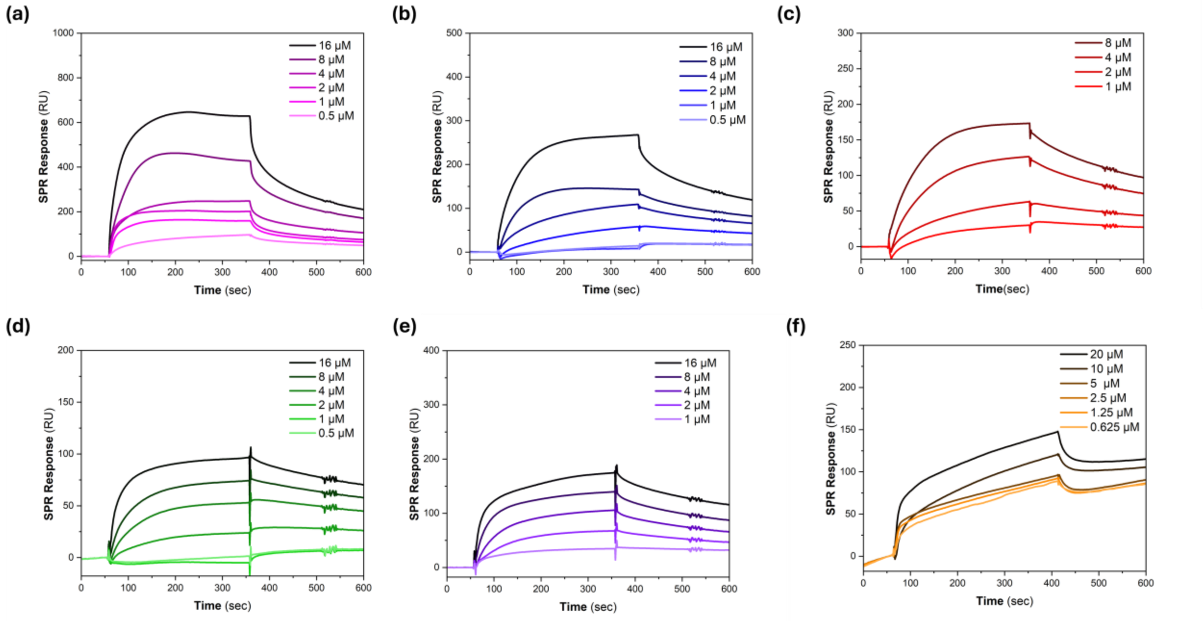

***Figure S8*. Sensorgrams depicting the association and dissociation states with Langerin** of **(a)** *(E)*-**P(GlcOx)$_{20}$MM**, **(b)** *(Z)*-**P(GlcOx)$_{20}$MM** (State A), **(c)** PSS^{365} (State B), **(d)** PSS^{302} (State C), **(e)** after THI process from PSS^{302} (State D), and **(f)** the control glycopolymer **P(GlcOx)$_{22}$**.

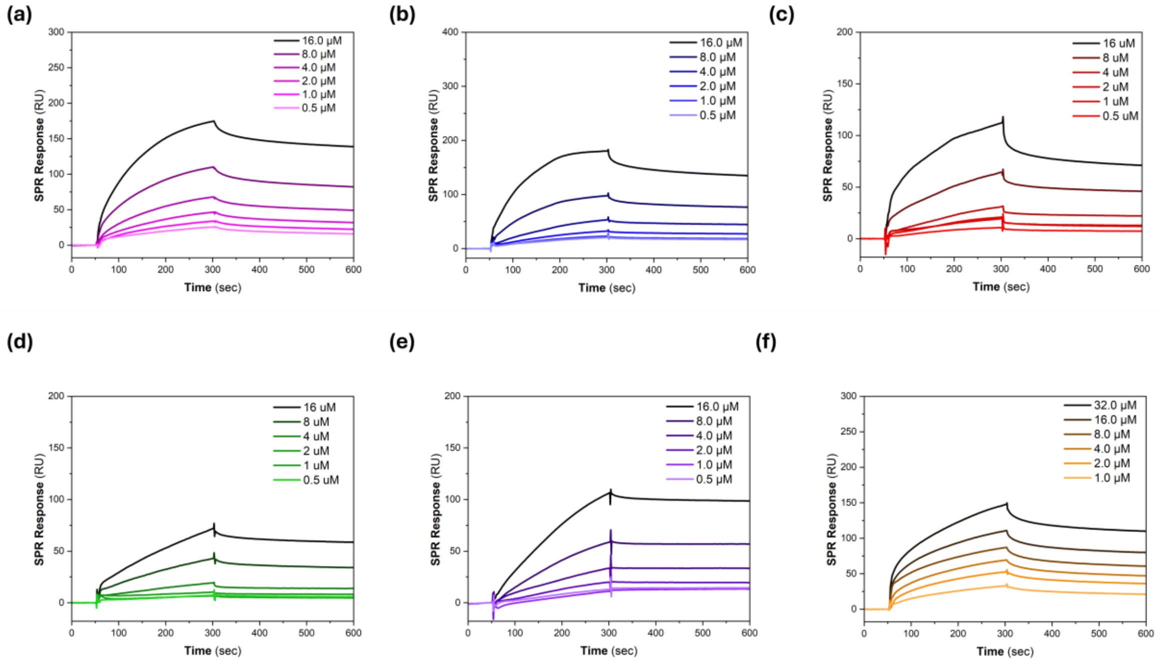

Figure S9. **Sensorgrams depicting the association and dissociation states with DC-SIGN** of **(a)** *(E)*-**P(ManOx)$_{22}$MM**, **(b)** *(Z)*-**P(ManOx)$_{22}$MM** (State A), **(c)** PSS^{365} (State B), **(d)** PSS^{302} (State C), **(e)** after THI process from PSS^{302} (State D), and **(f)** the control glycopolymer **P(ManOx)$_{22}$**.

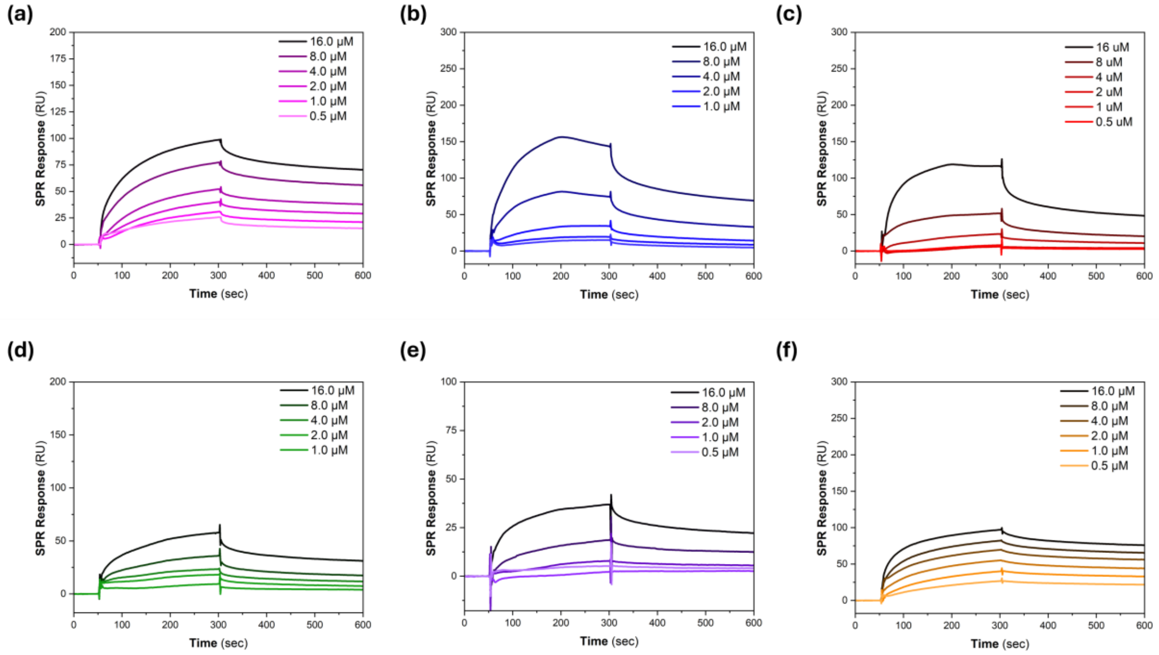

Figure S10. **Sensorgrams depicting the association and dissociation states with Langerin** of **(a)** *(E)*-**P(ManOx)$_{22}$MM**, **(b)** *(Z)*-**P(ManOx)$_{22}$MM** (State A), **(c)** PSS^{365} (State B), **(d)** PSS^{302} (State C), **(e)** after THI process from PSS^{302} (State D), and **(f)** the control glycopolymer **P(ManOx)$_{22}$**.

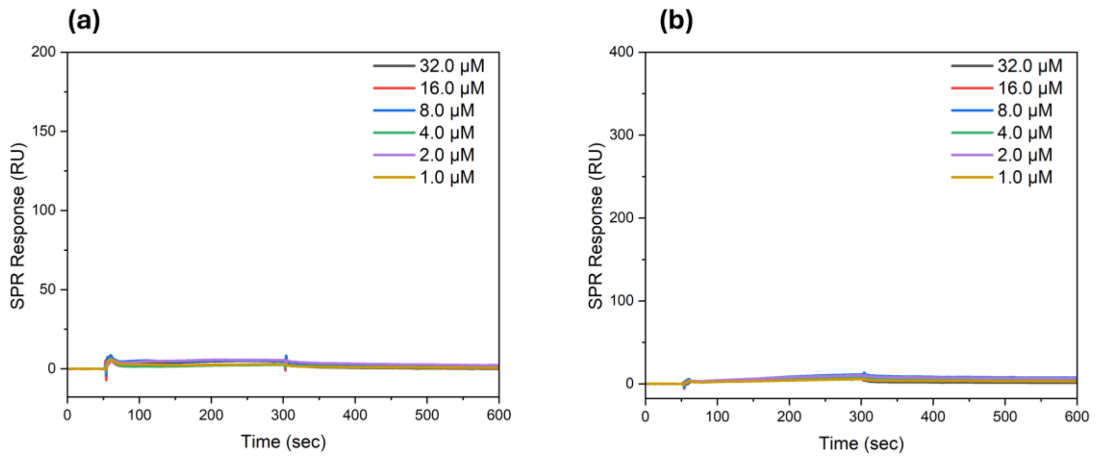

Figure S11. The negative control (**PEtOx$_{25}$**) against Langerin and DC-SIGN. **(a)** The negative control flown with DC-SIGN. **(b)** The negative control flown over Langerin.

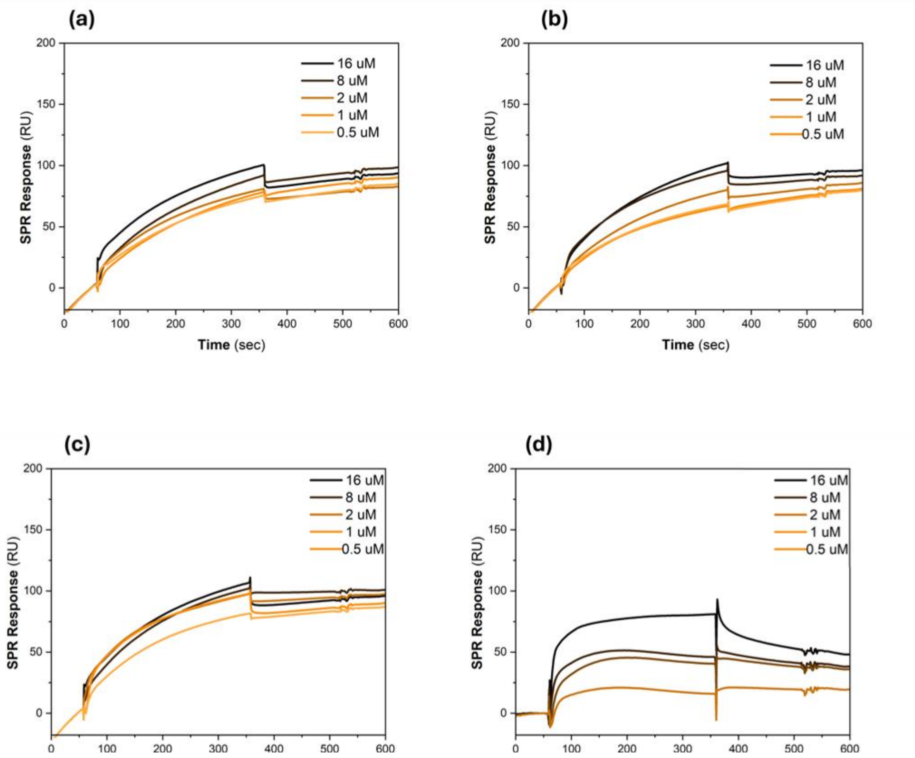

Figure S12. Sensorgrams of the control polymer **(GlcOx)$_{22}$** undergoing the same rotation steps as the motors to demonstrate minimal effects. Whereby **(a)** is **(GlcOx)$_{22}$** with no uv or thermal interaction **(b)** is after being irradiated with 365 nm light **(c)** Is after being irradiated with 306 nm light and **(d)** is after being heated at 70 ˚C overnight. Although **(d)** looks different to the rest this is attributed to the baseline correction, without it looks the same.

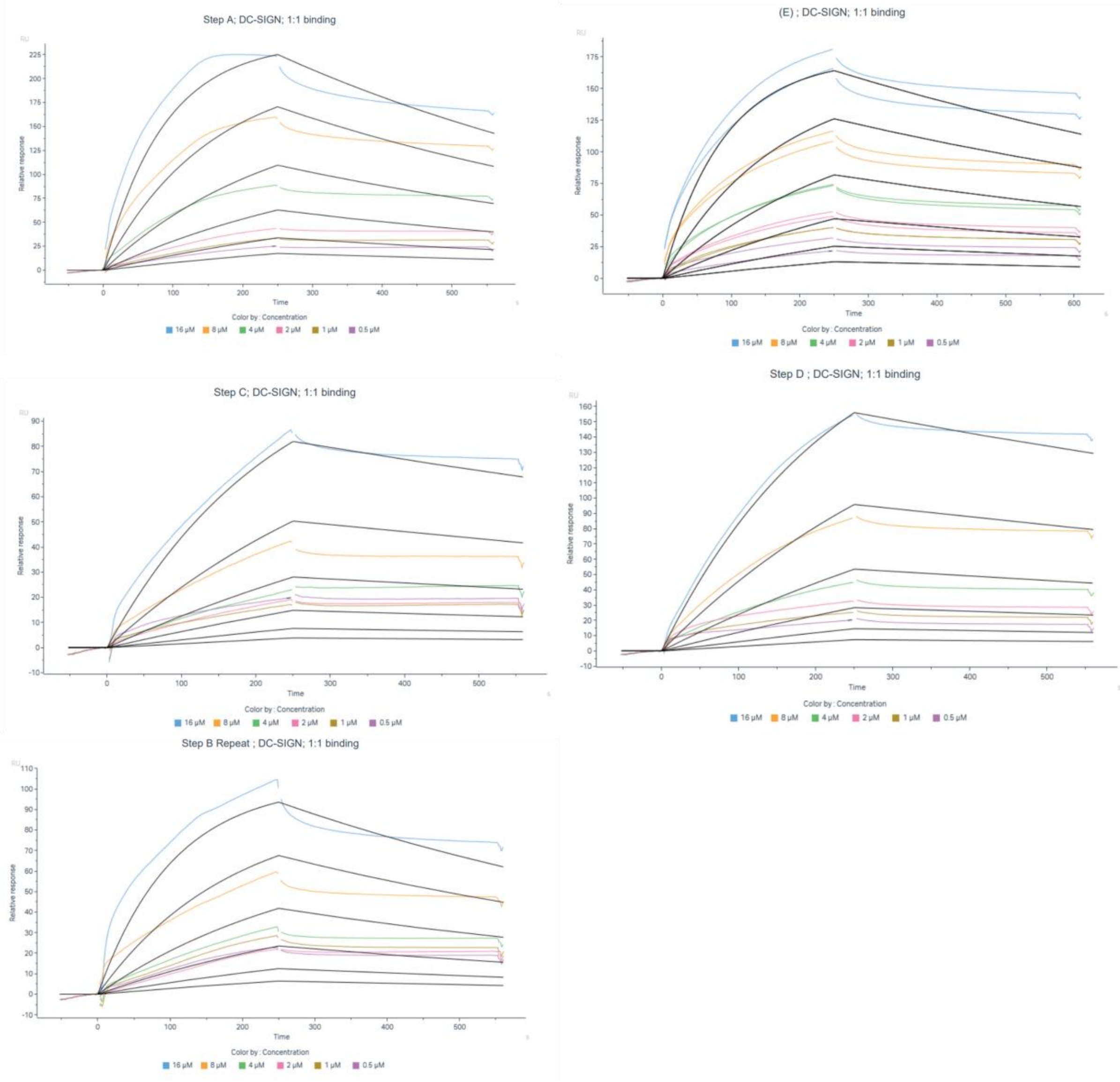

Figure S13. Example sensorgram plots with their associated Langmuir 1:1 fits of the mannose glycopolymers against DC-SIGN. See 5.4 for interpretations of the kinetic fits.

5.5. Sensorgrams from competition assay

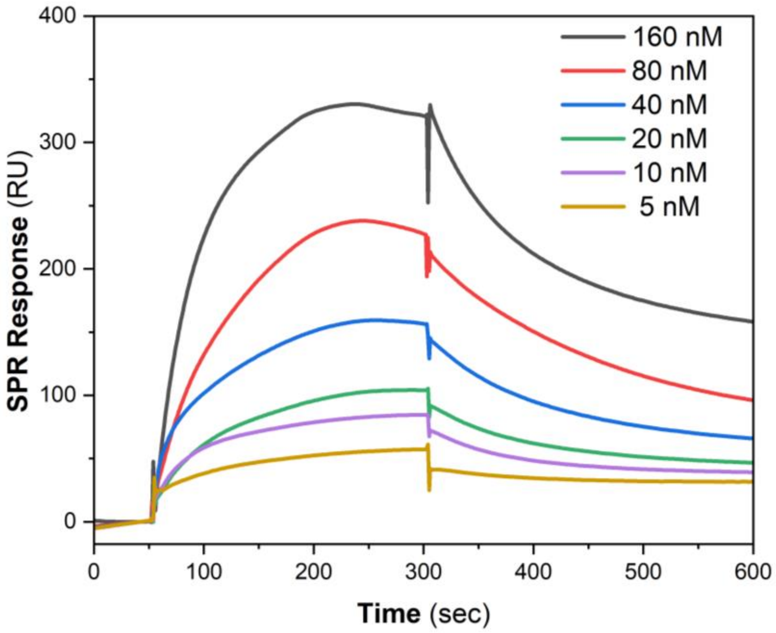

Figure S14. Sensorgrams depicting the association and dissociation rates of DC-SIGN (concentrations indicated) binding with gp120 in the absence of polymer.

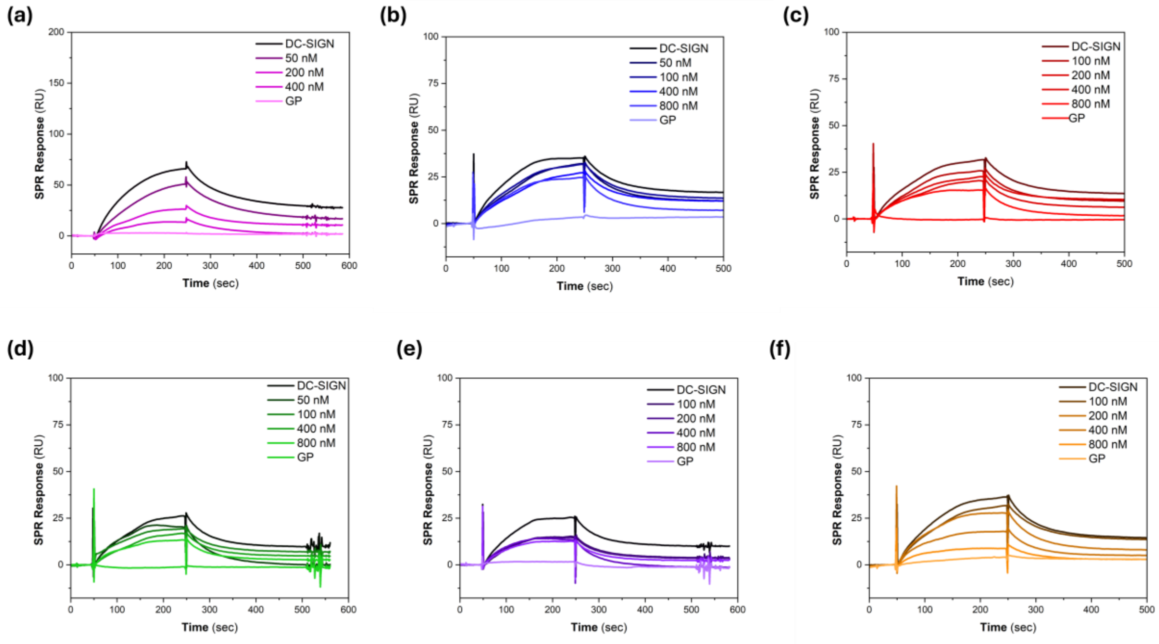

Figure S15. **Sensorgrams depicting the competition between mannoside-containing glycopolymers and the glycoprotein gp120. As the polymer (in solution) concentrations rise and subsequently interact with DC-SIGN, this inhibits the binding of DC-SIGN to gp120 (immobilized on the chip).(a)** *(E)*-**P(ManOx)$_{22}$MM**, **(b)** *(Z)*-**P(ManOx)$_{22}$MM** (State A), **(c)** PSS^{365} (State B), **(d)** PSS^{302} (State C), **(e)** after THI process from PSS^{302} (State D), and **(f)** the control glycopolymer **P(ManOx)$_{22}$**. Where GP (800 nM) signifies the glycopolymer with no additional DC-SIGN (40 nM) injected. Where GP (800 nM) signifies the glycopolymer of each isomer alone with no additional DC-SIGN injected. Blank subtractions were done using a buffer alone sample, which was run at the start of each individual experiment. DC-SIGN (40 nM) signifies the run of DC-SIGN alone for each of the individual experiment which was used to calculate the relative Rmax change.

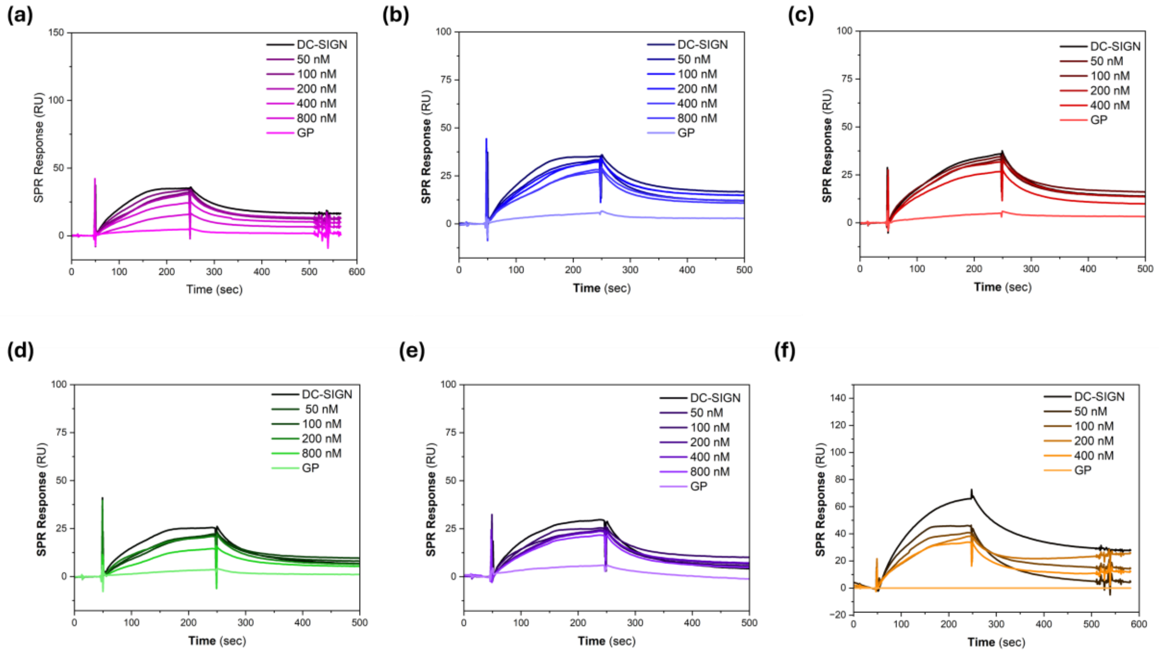

***Figure S16*. Sensorgrams depicting the competition between glucoside-containing glycopolymers and the glycoprotein gp120. As the polymer (in solution) concentrations rise and subsequently interact with DC-SIGN, this inhibits the binding of DC-SIGN to gp120 (immobilized on the chip).((a)** *(E)*-**P(GlcOx)$_{22}$MM**, **(b)** *(Z)*-**P(GlcOx)$_{22}$MM** (State A), **(c)** PSS^{365} (State B), **(d)** PSS^{302} (State C), **(e)** after THI process from PSS^{302} (State D), and **(f)** the control glycopolymer **P(GlcOx)$_{22}$**. Where GP (800 nM) signifies the glycopolymer of each isomer alone with no additional DC-SIGN injected. Blank subtractions were done using a buffer alone sample, which was run at the start of each individual experiment. DC-SIGN (40 nM) signifies the run of DC-SIGN alone for each of the individual experiment which was used to calculate the relative Rmax change.

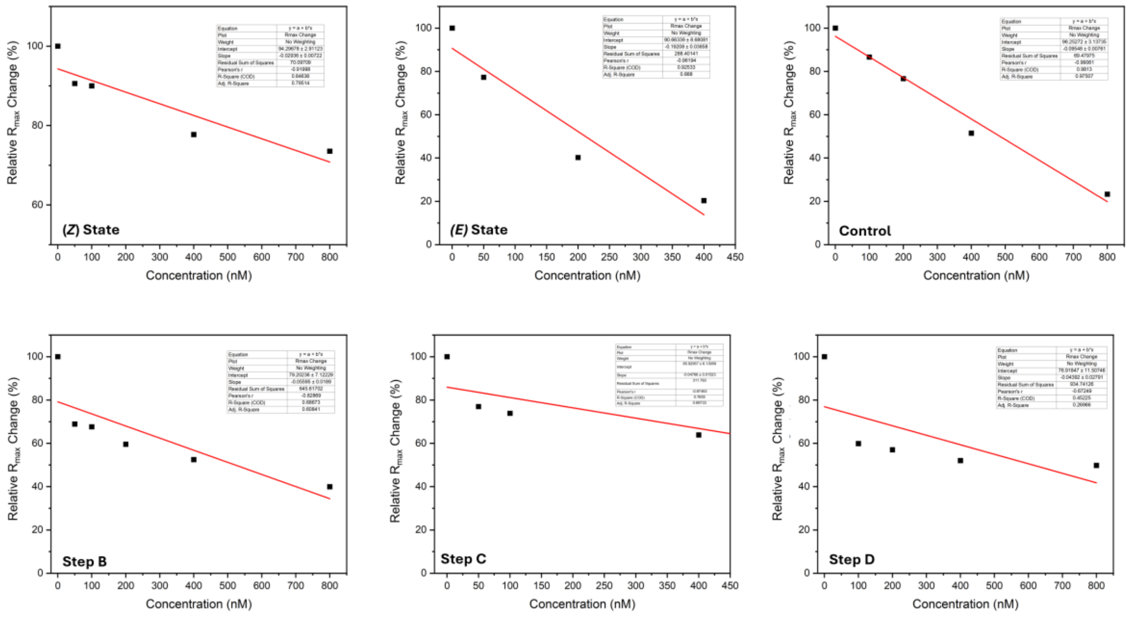

Figure S17. Plotted relative R_{max} change (%) vs concentration (nM) with the linear line of best fit used to calculate the IC_{50} values for the mannose containing glycopolymers.

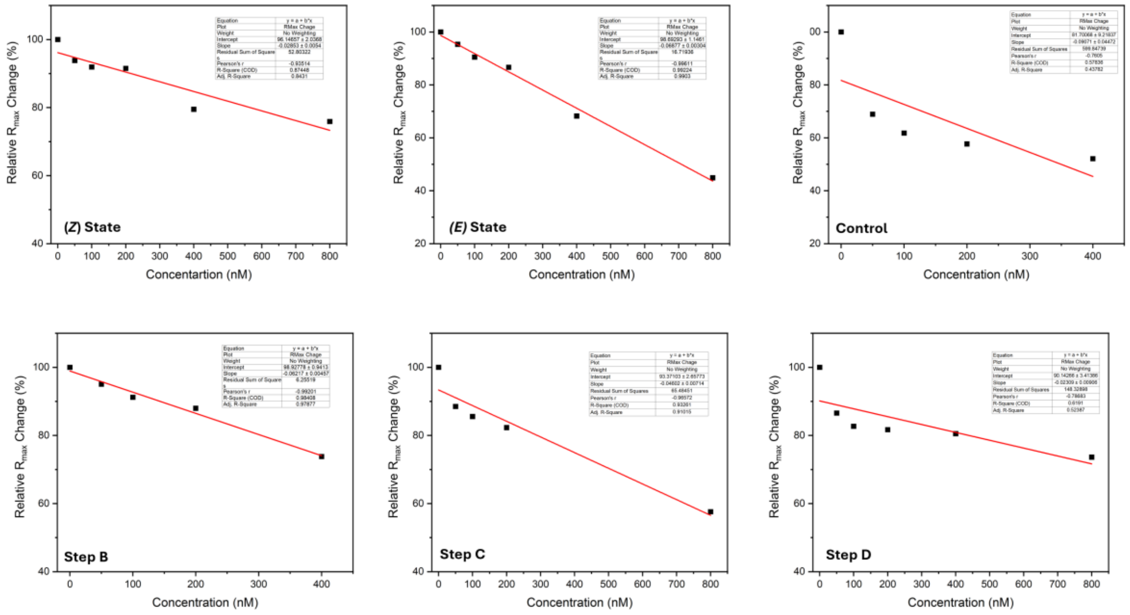

Figure S18. Plotted relative R_{max} change (%) vs concentration (nM) with the linear line of best fit used to calculate the IC_{50} values for the glucose containing glycopolymers.

6. Analytical data

6.1. Proton and carbon nuclear magnetic resonance spectroscopy (^{1}H NMR and ^{13}C NMR)

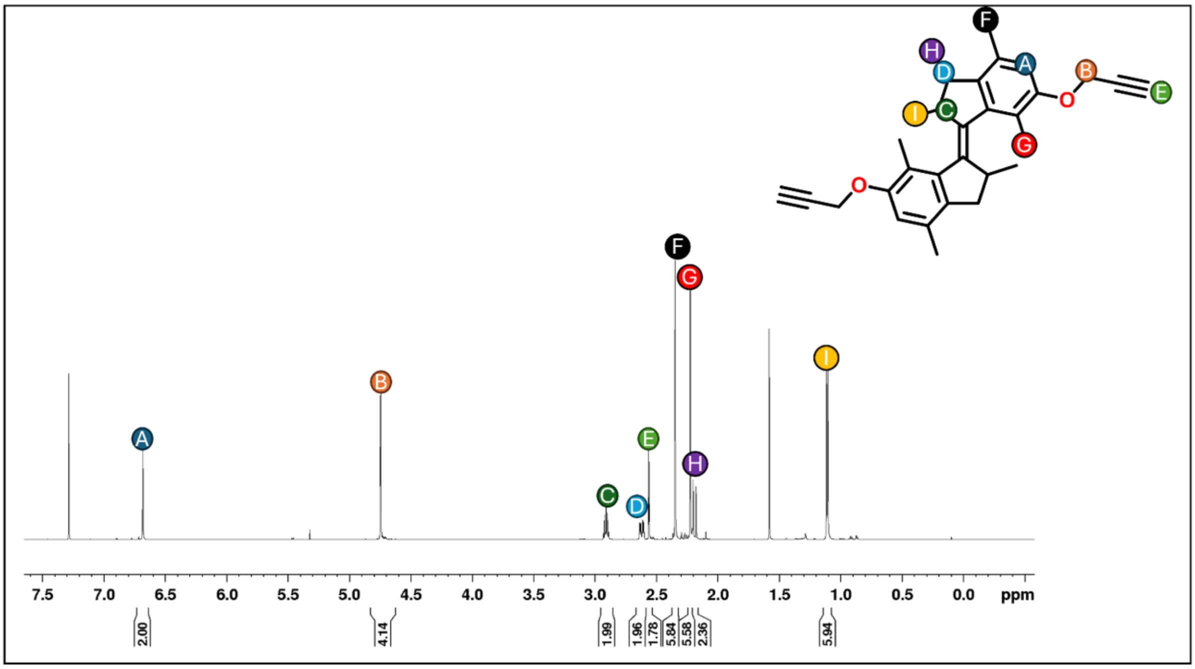

Figure S19. The assigned ^{1}H NMR spectrum of bis-propargyl motor *(E)*-**B4** (600 MHz, $CDCl_3$, 25 °C).

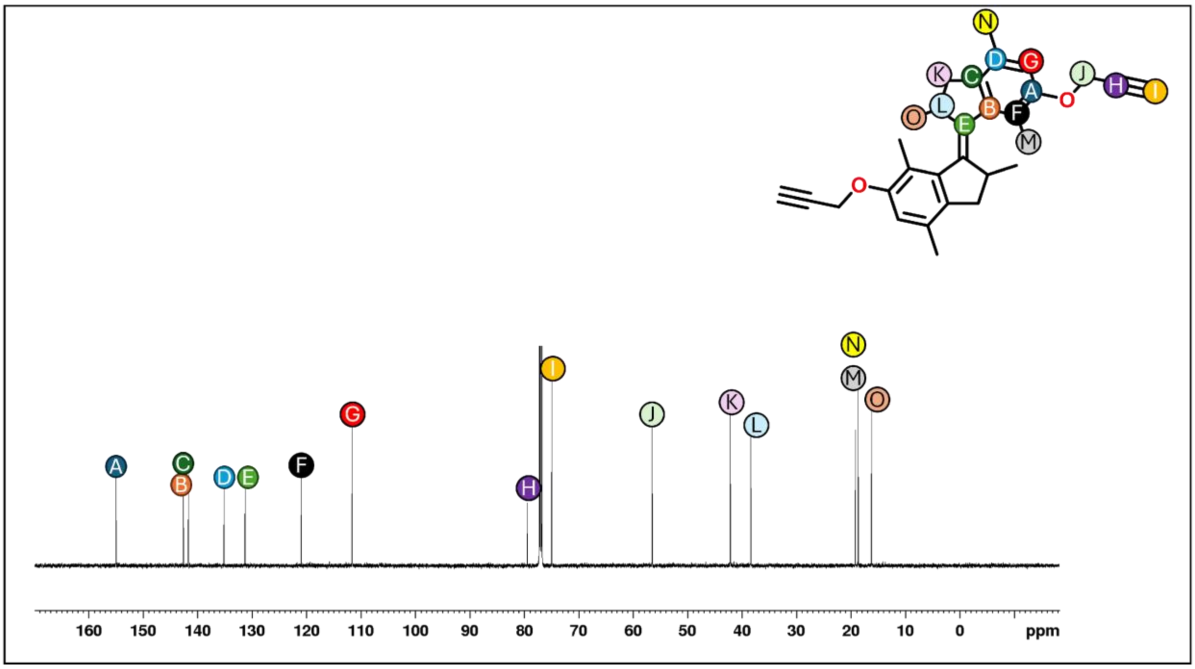

Figure S20. The assigned ^{13}C NMR spectrum of bis-propargyl motor *(E)*-**B4** (151 MHz, $CDCl_3$, 25 °C).

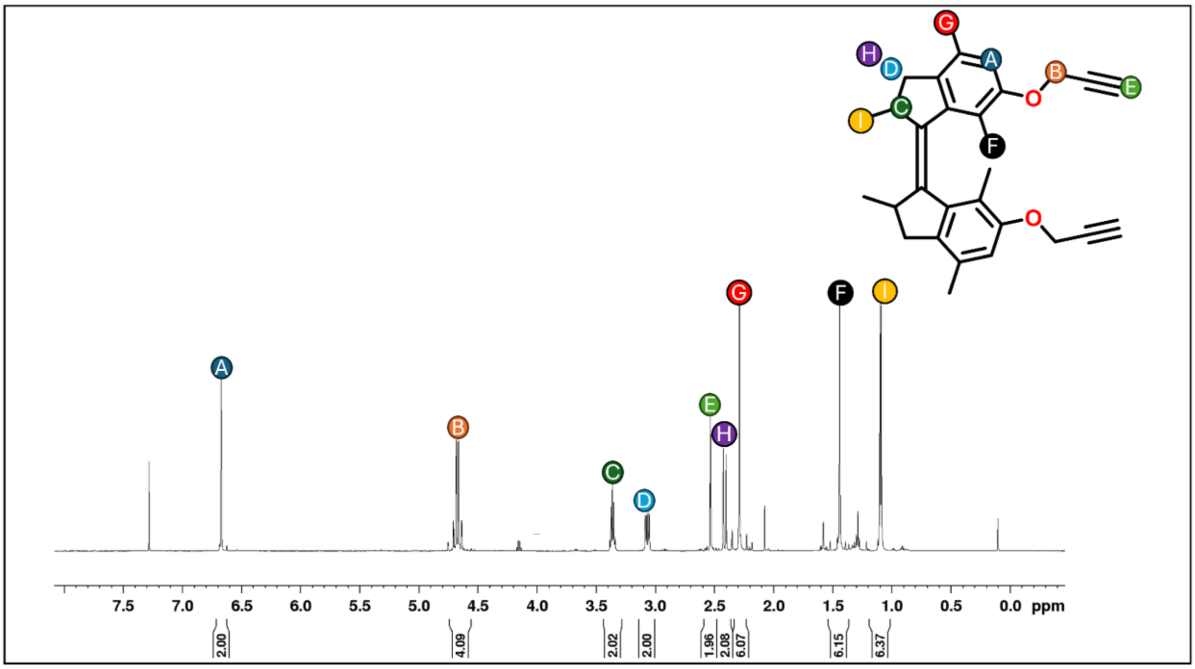

Figure S21. The assigned ^{1}H NMR spectrum of bis-propargyl motor *(Z)*-**B4** (600 MHz, $CDCl_3$, 25 °C).

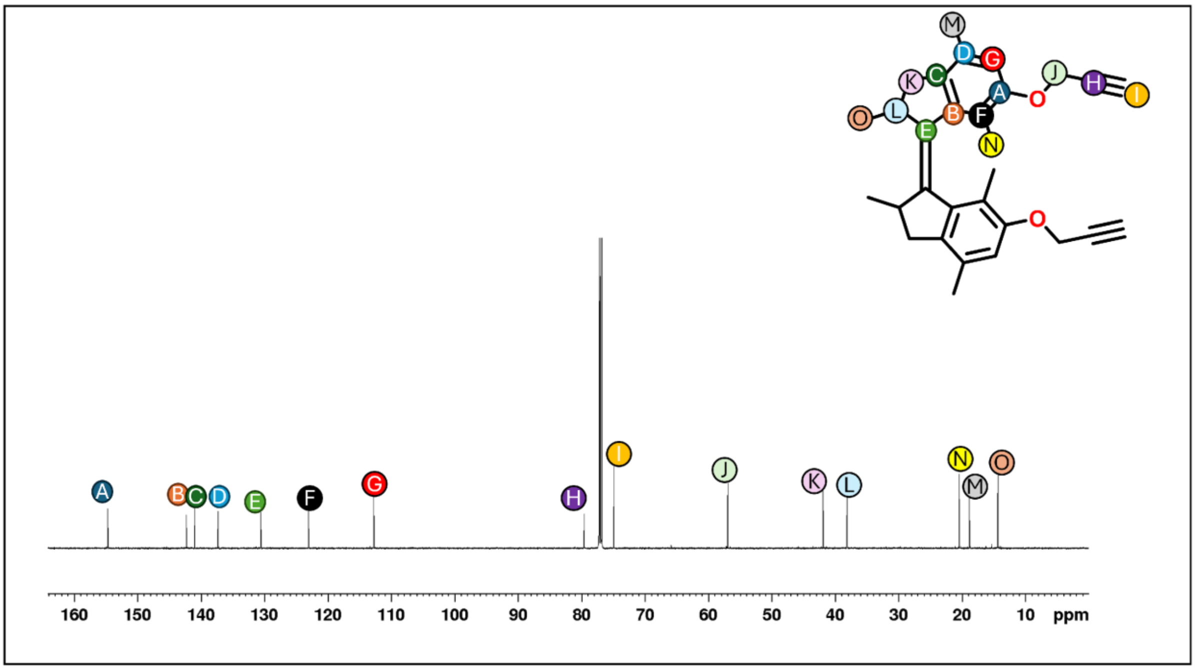

Figure S22. The assigned ^{13}C NMR spectrum of bis-propargyl motor *(Z)*-**B4** (151 MHz, $CDCl_3$, 25 °C).

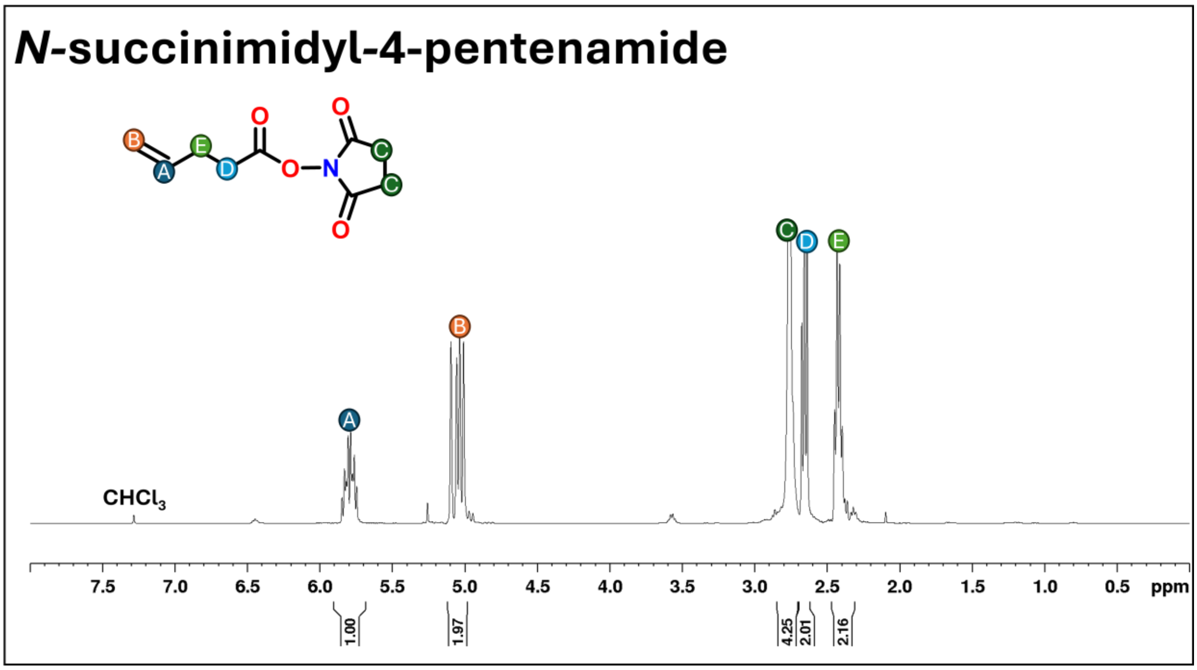

Figure S23. The assigned ^{1}H NMR spectrum of N-succinimidyl-4-pentenamide (400 MHz, $CDCl_3$, 25 °C).

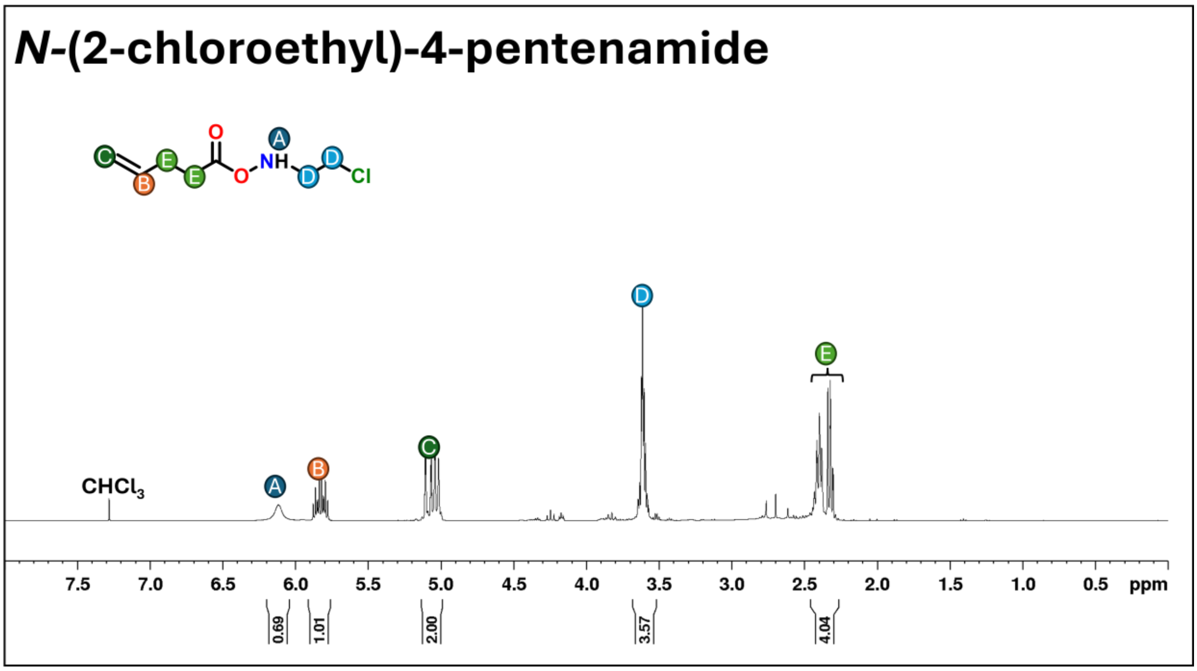

Figure S24. The assigned ^{1}H NMR spectrum of N-(2-chloroethyl)-4-pentenamide (400 MHz, $CDCl_3$, 25 °C).

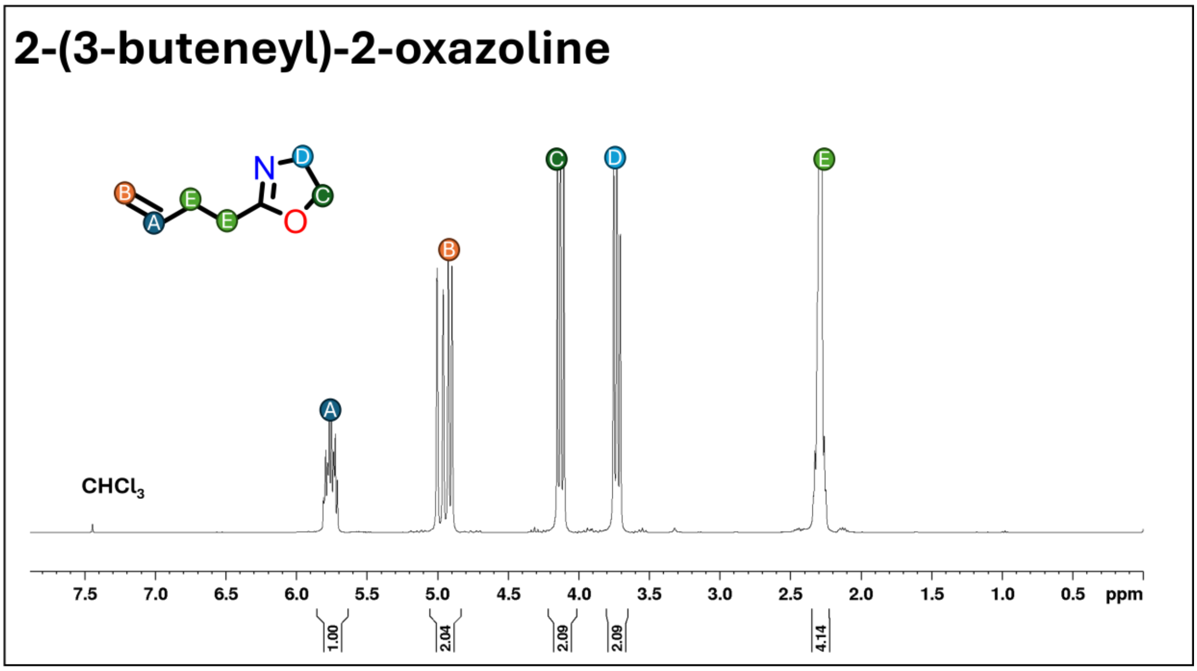

Figure S25. The assigned ^{1}H NMR spectrum of 2-(3-butenyl)-2-oxazoline (400 MHz, $CDCl_3$, 25 °C).

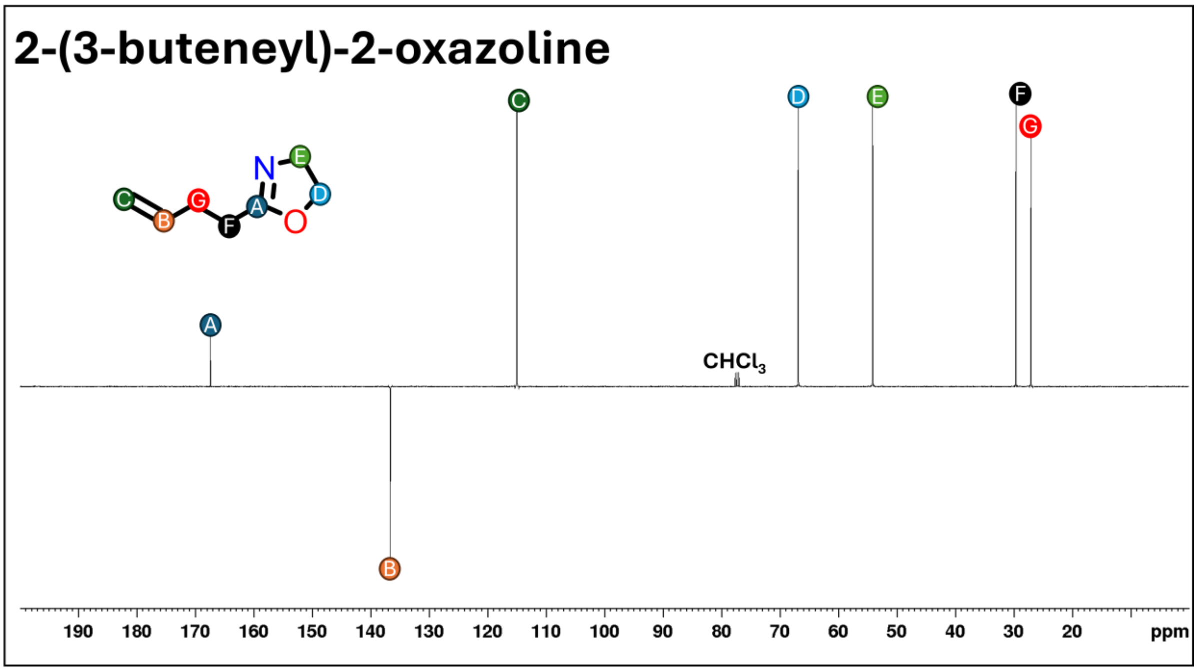

Figure S26. The assigned ^{13}C NMR spectrum of of 2-(3-butenyl)-2-oxazoline (151 MHz, $CDCl_3$, 25 °C).

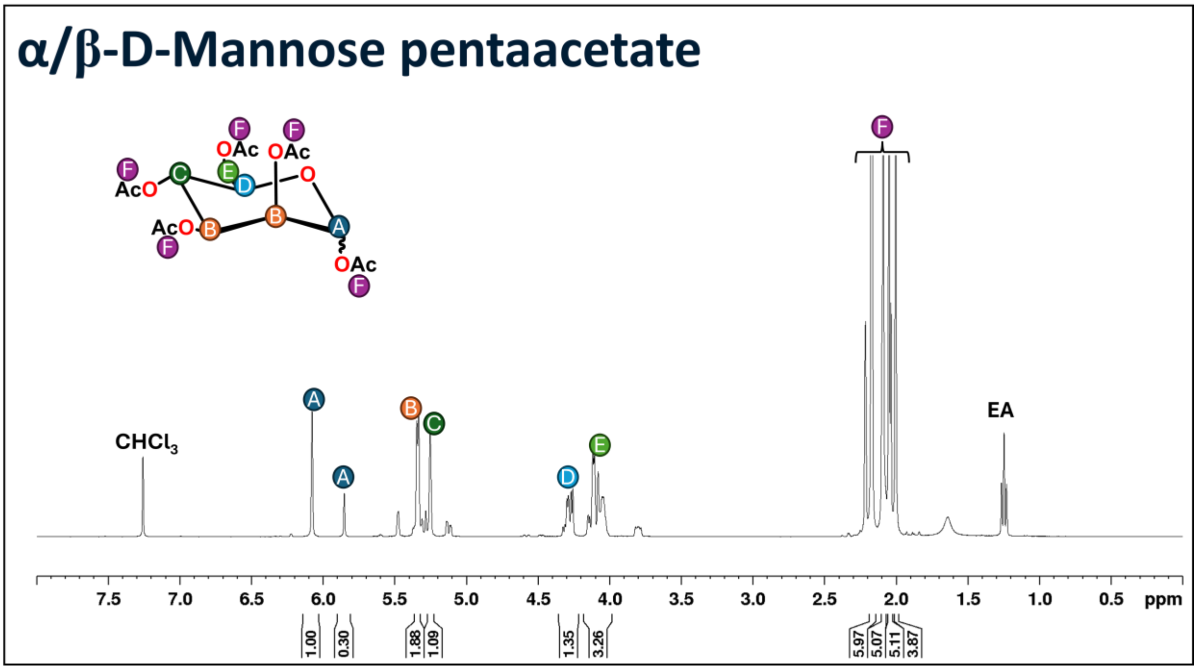

Figure S27: The assigned ^{1}H NMR spectrum of α/β-D-Mannose pentaacetate (400 MHz, $CDCl_3$, 25 °C). Where EA corresponds to ethyl acetate, with the other peaks associated covered by sugar peaks.

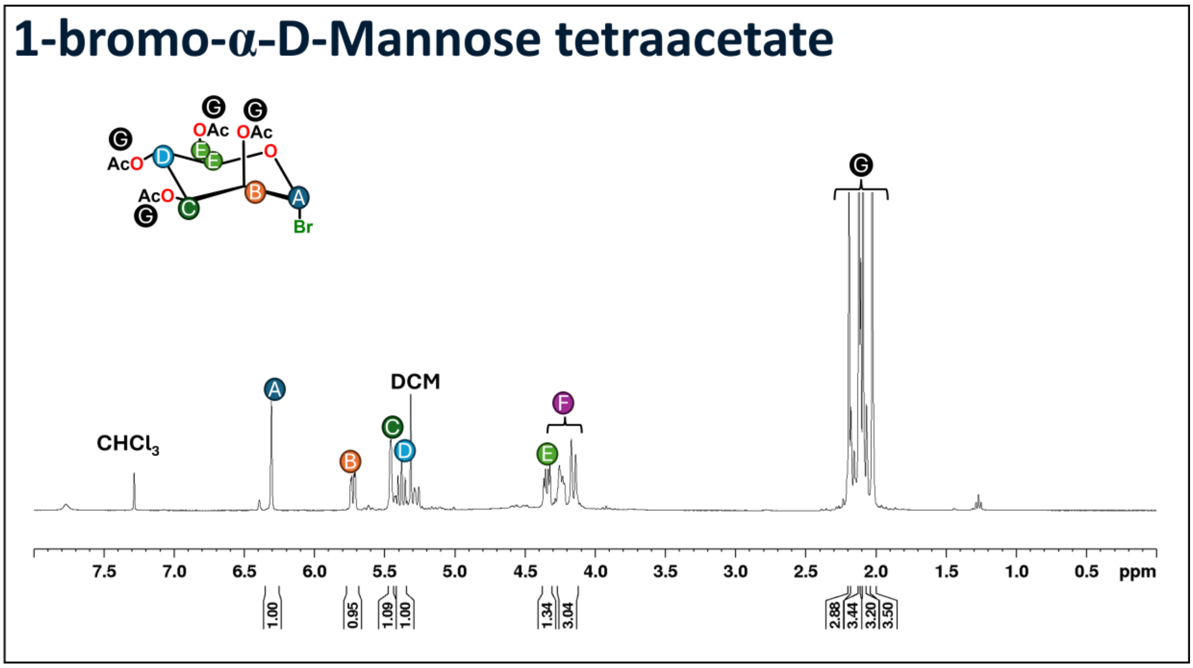

Figure S28: The assigned ^{1}H NMR spectrum of 1-bromo-α-D-mannose tetraacetate (400 MHz, $CDCl_3$, 25 °C). Where DCM correspond to dichloromethane.

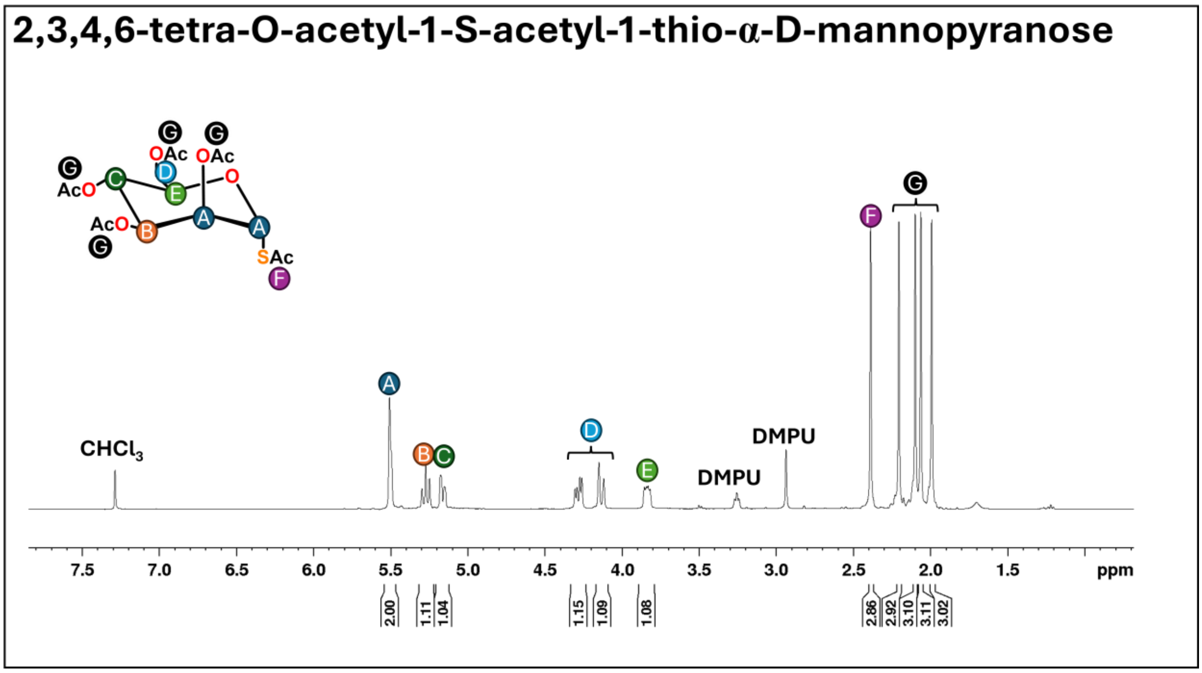

Figure S29: The assigned ^{1}H NMR spectrum of 2,3,4,6-tetra-O-acetyl-1-S-acetyl-1-thio-α-D-Mannopyranose (400 MHz, $CDCl_3$, 25 °C). Where DMPU corresponds to 1,3-dimethyl-3,4,5,6-tetrahydro-2(1H)-pyrimidinone.

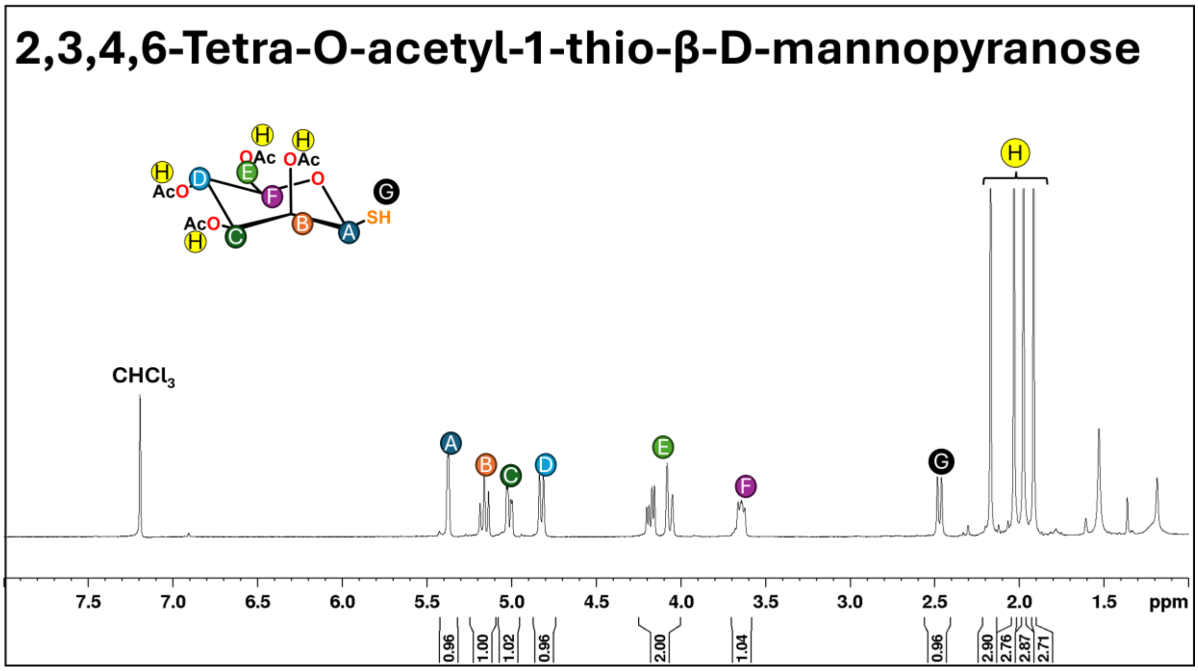

Figure S30: The assigned ^{1}H NMR spectrum of 2,3,4,6-tetra-O-acetyl-1-thio-α-D-Mannopyranose (400 MHz, $CDCl_3$, 25 °C).

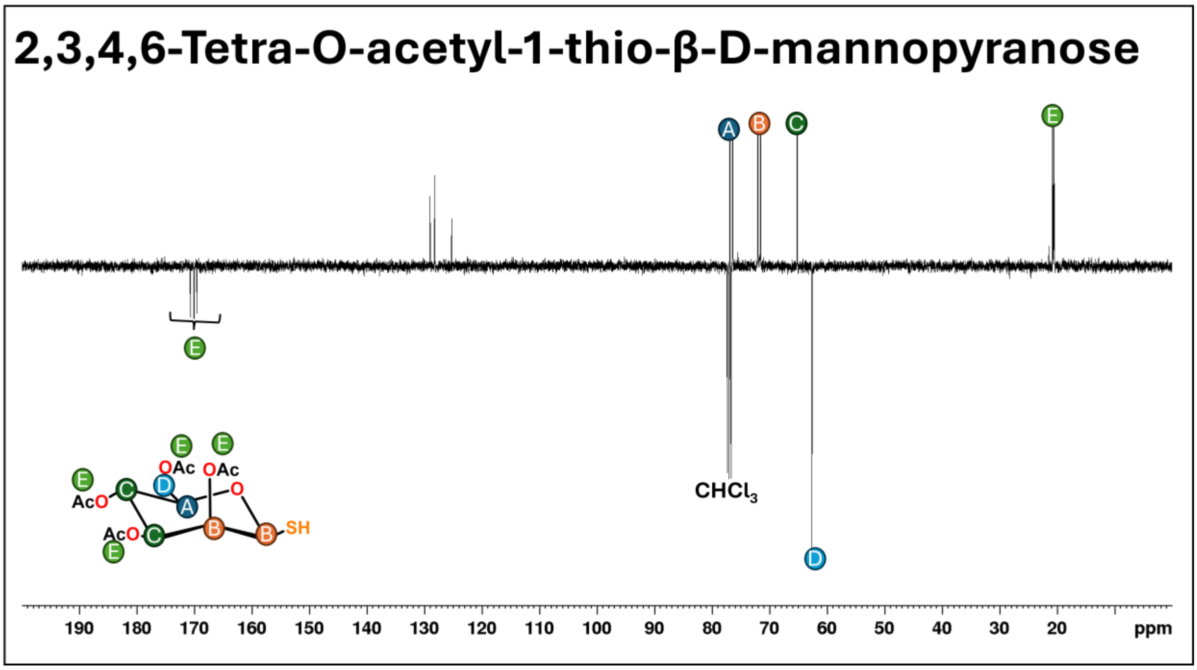

Figure S31. The assigned ^{13}C NMR spectrum of 2,3,4,6-tetra-O-acetyl-1-thio-α-D-Mannopyranose (151 MHz, $CDCl_3$, 25 °C).

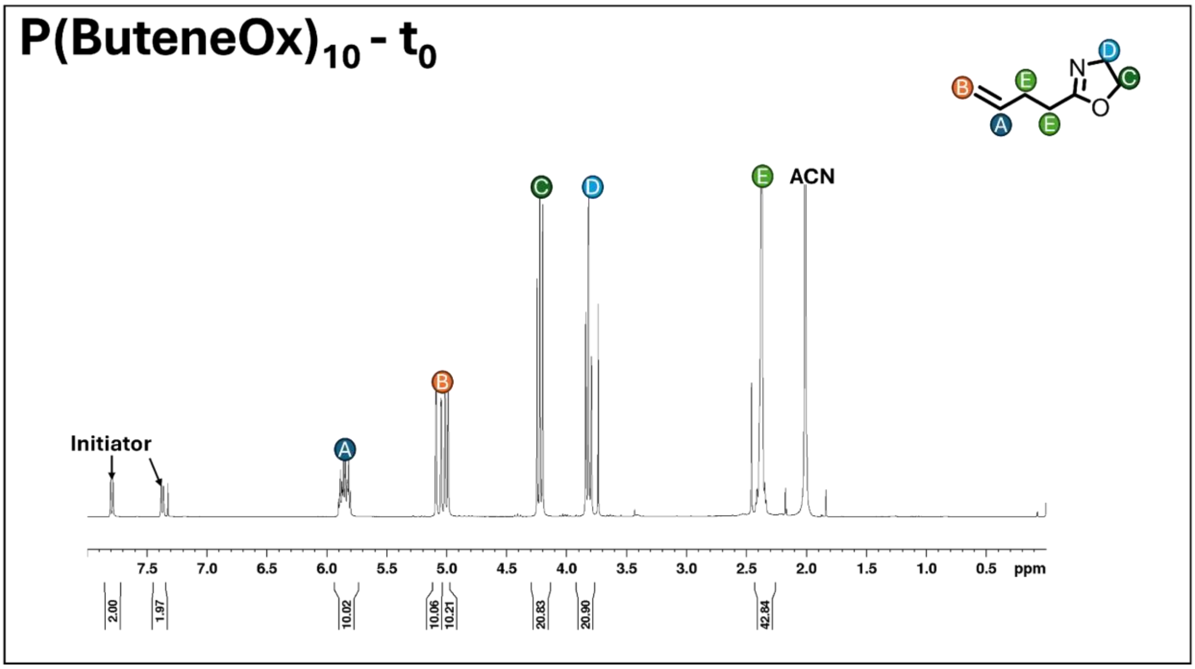

Figure S32. The assigned t_0 ^1H NMR spectrum of **P(^{n}ButeneOx)$_{10}$** before polymerization (400 MHz, $CDCl_3$, 25 °C). Where ACN corresponds to acetonitrile.

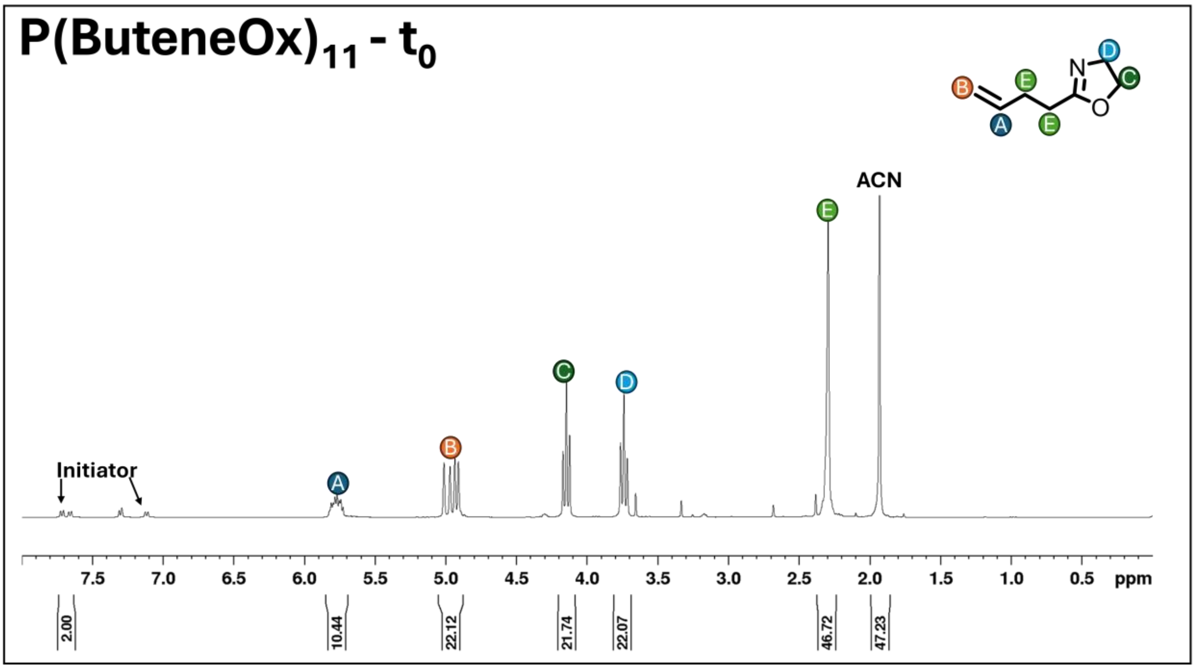

Figure S33. The assigned t_0 ^1H NMR spectrum of **P(^{n}ButeneOx)$_{11}$** before polymerization (400 MHz, $CDCl_3$, 25 °C). Where ACN corresponds to acetonitrile.

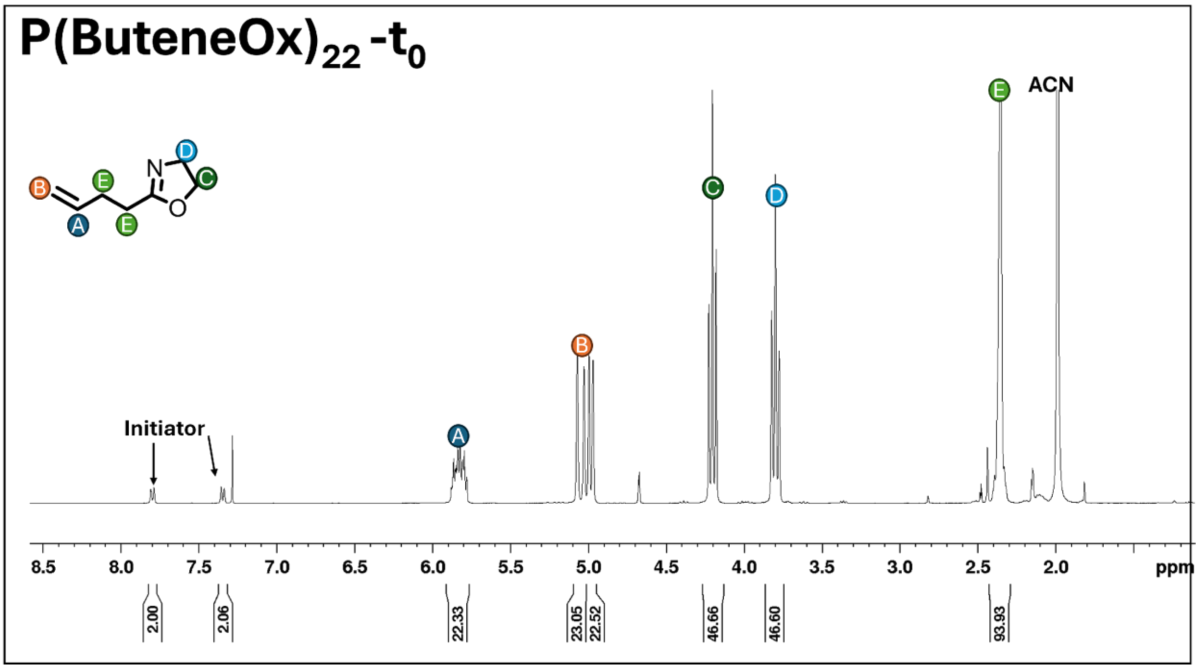

Figure S34. The assigned t₀ ^{1}H NMR spectrum of **P(^{n}ButeneOx)$_{22}$** before polymerization (400 MHz, $CDCl_3$, 25 °C). Where ACN corresponds to acetonitrile.

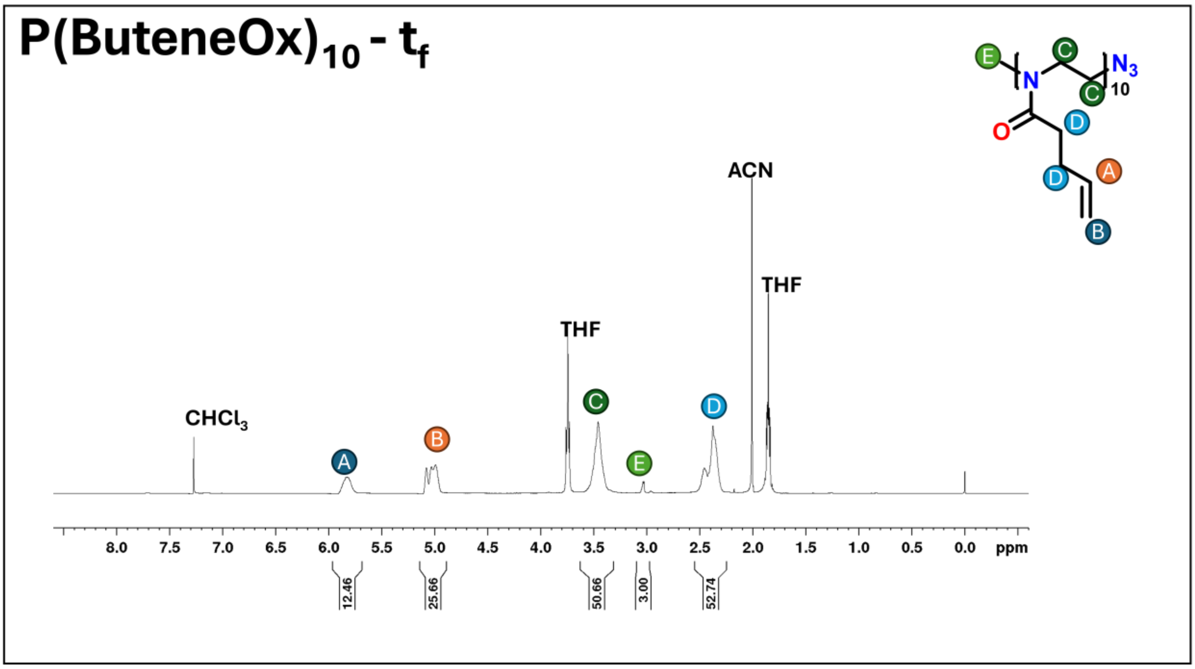

Figure S35. The assigned t_f ^{1}H NMR spectrum of the **P(^{n}ButeneOx)$_{10}$** (400 MHz, $CDCl_3$, 25 °C). Where ACN and THF correspond respectively to acetonitrile and tetrahydrofuran.

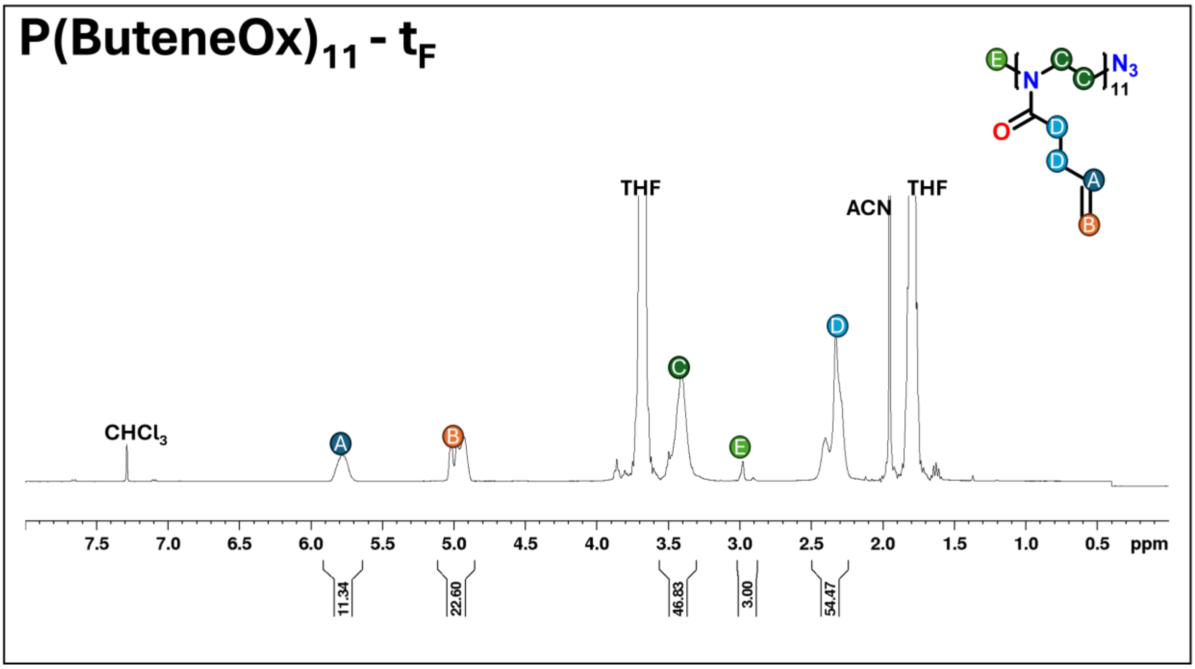

Figure S36. The assigned t$_f$ ^{1}H NMR spectrum of the **P(^{n}ButeneOx)$_{11}$** (400 MHz, $CDCl_3$, 25 °C). Where ACN and THF correspond respectively to acetonitrile and tetrahydrofuran.

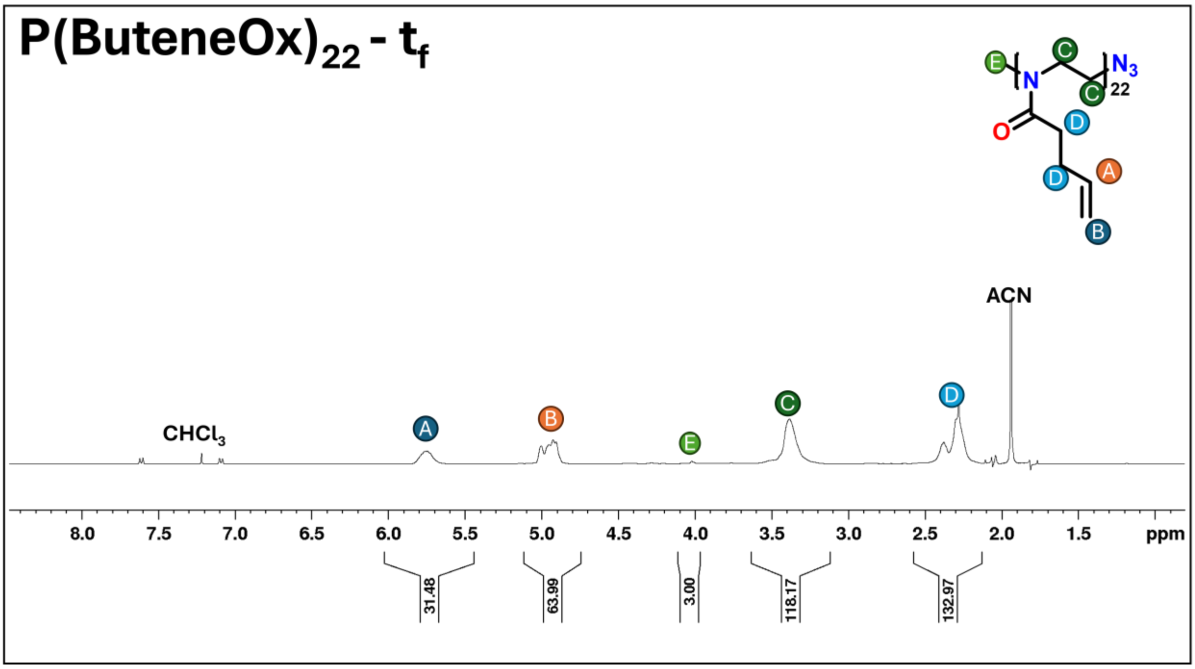

Figure S37. The assigned t$_f$ ^{1}H NMR spectrum of the **P(^{n}ButeneOx)$_{22}$** (400 MHz, $CDCl_3$, 25 °C). Where ACN and corresponds to acetonitrile.

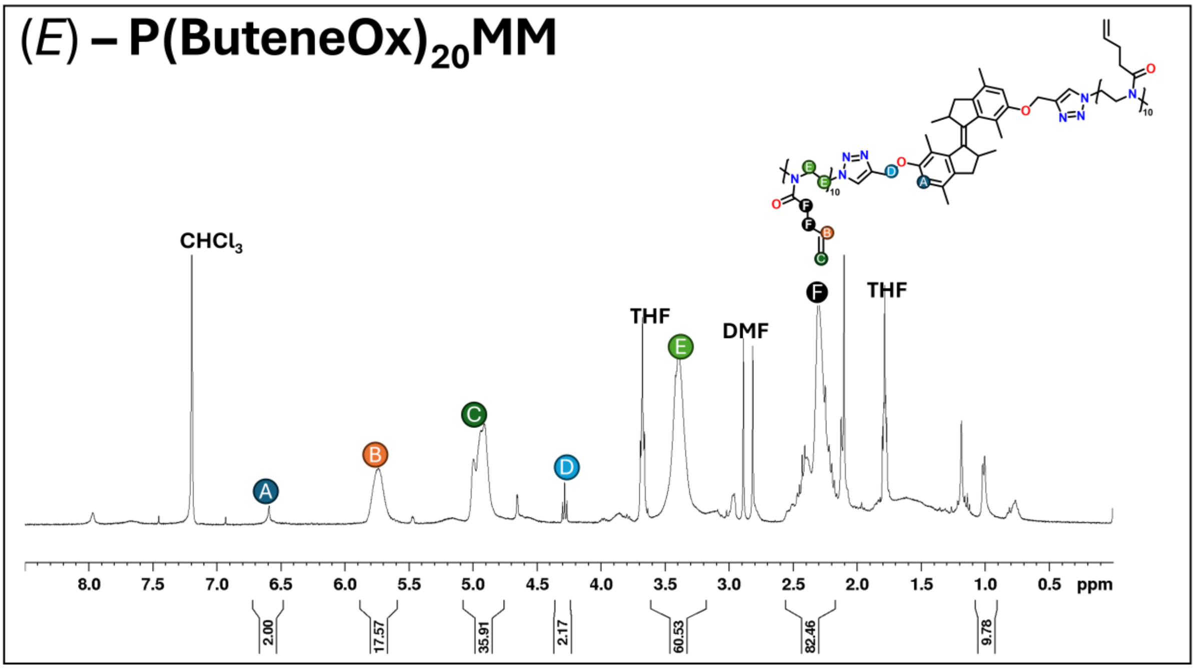

Figure S38. The assigned tf ^{1}H NMR spectrum of *(E)*-**P(^{n}ButeneOx)$_{20}$MM** (400 MHz, $CDCl_3$, 25 °C). Where THF and DMF respectively correspond to tetrahydrofuran and dimethylformamide.

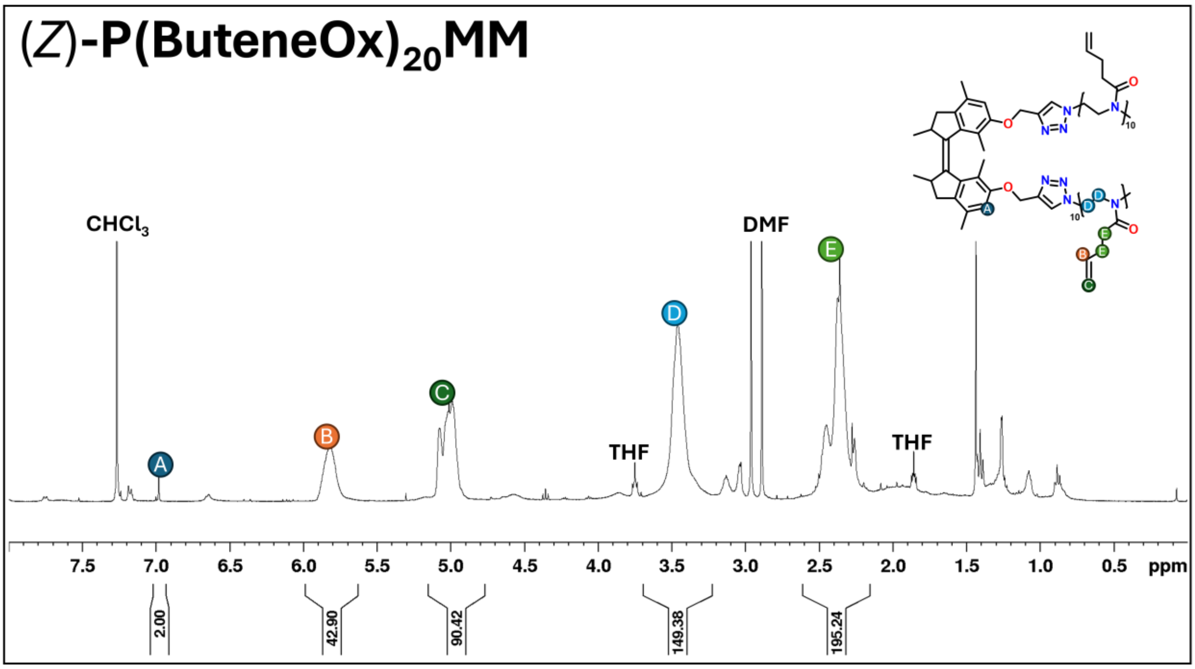

Figure S39. The assigned ^{1}H NMR spectrum of *(Z)*-**P(^{n}ButeneOx)$_{20}$MM** (400 MHz, $CDCl_3$, 25 °C). Where THF and DMF respectively correspond to tetrahydrofuran and dimethylformamide.

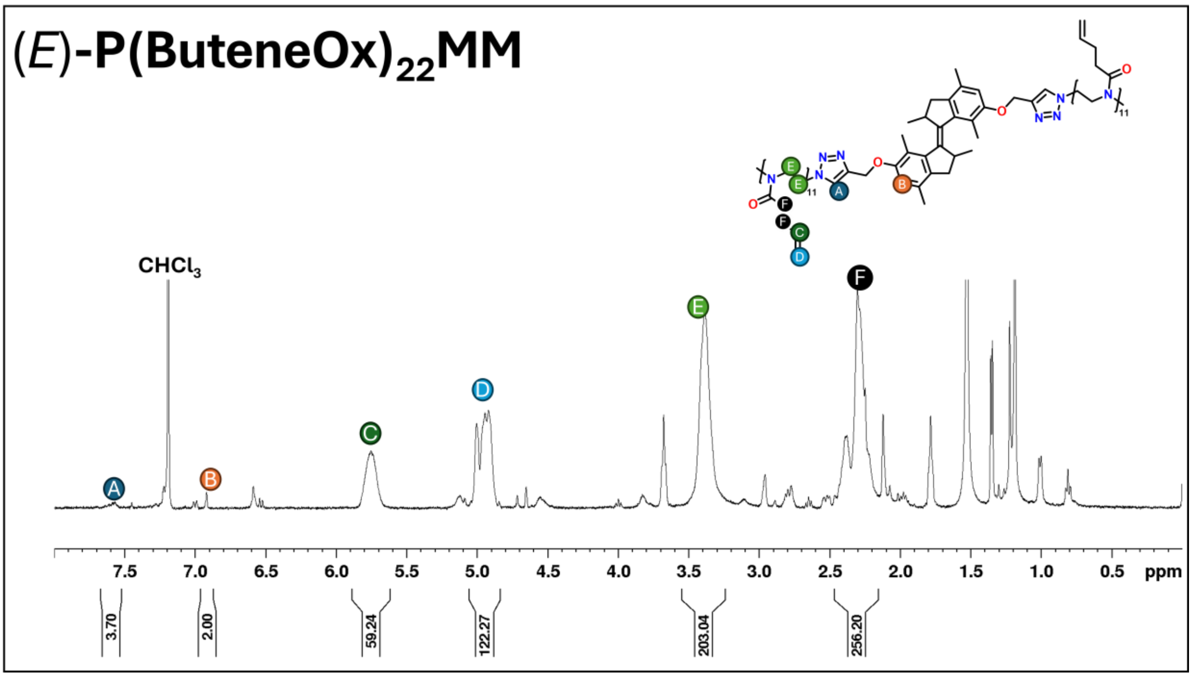

Figure S40. The assigned tf ^{1}H NMR spectrum of *(E)*-**P(*ⁿ*ButeneOx)$_{22}$MM** (400 MHz, $CDCl_3$, 25 °C).

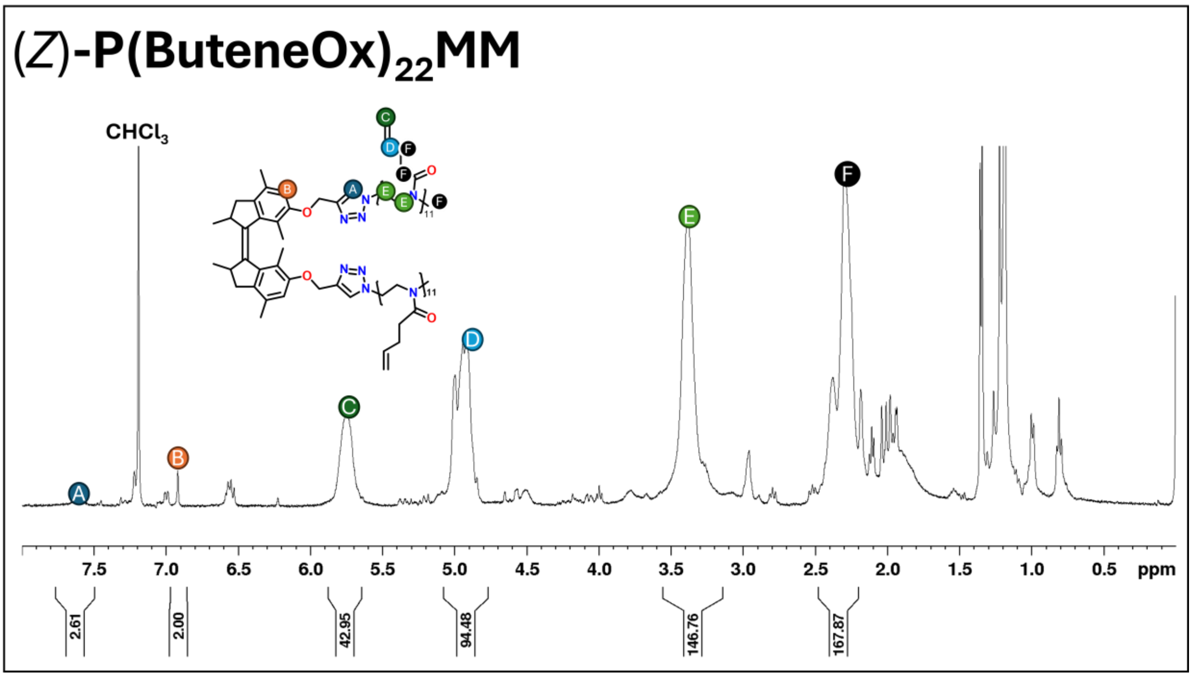

Figure S41. The assigned ^{1}H NMR spectrum of *(Z)*-**P(^{n}ButeneOx)$_{22}$MM** (400 MHz, $CDCl_3$, 25 °C).

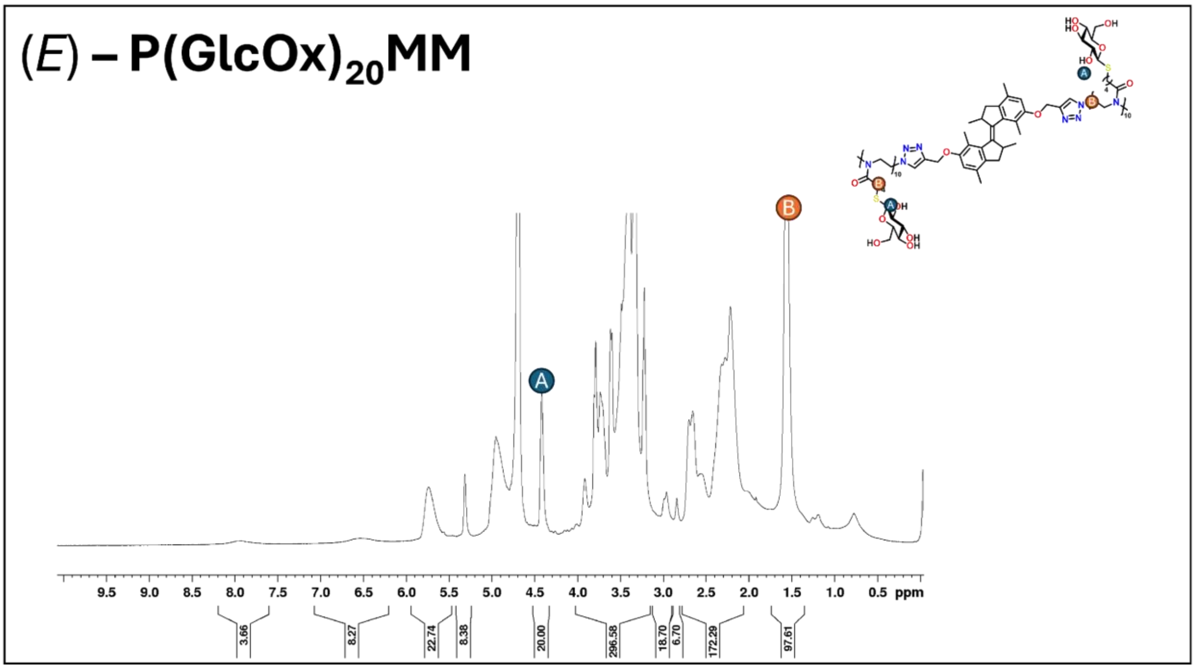

Figure S42. The partially assigned ^{1}H NMR spectrum of *(E)*-**P(GlcOx)$_{20}$MM** (600 MHz, D_2O:CD_3OD 99:1 V:V, 25 °C).

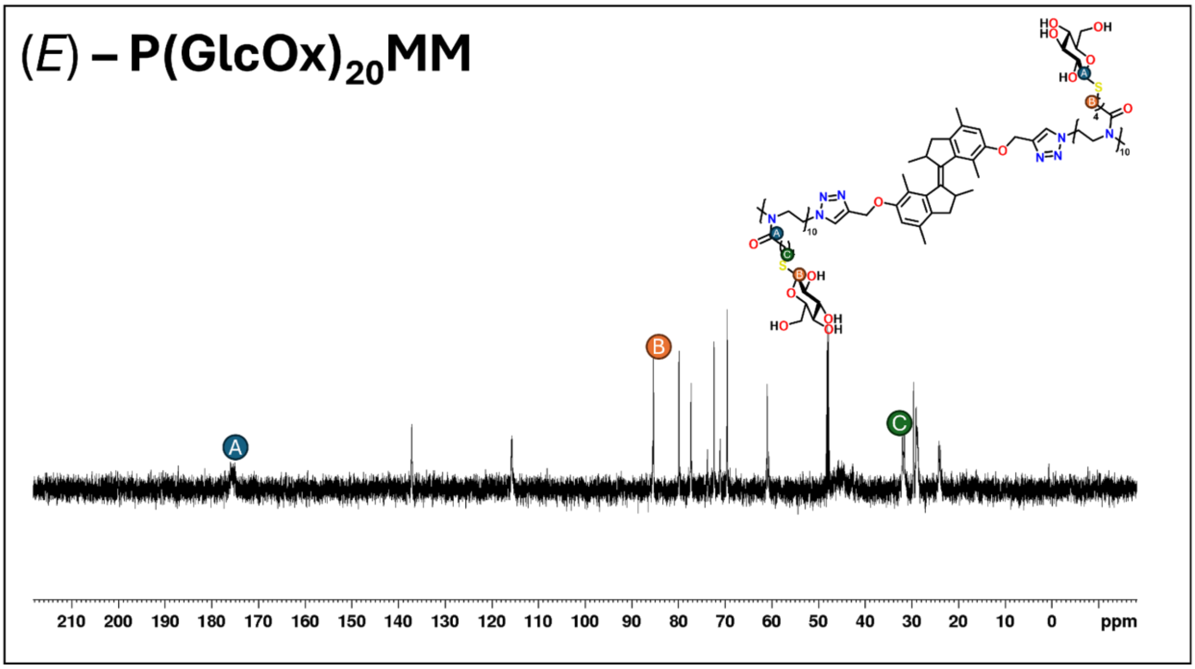

Figure S43. The partially assigned ^{13}C NMR spectrum of *(E)*-**P(GlcOx)$_{20}$MM** (151 MHz, D_2O:CD_3OD 99:1 V:V, 25°C).

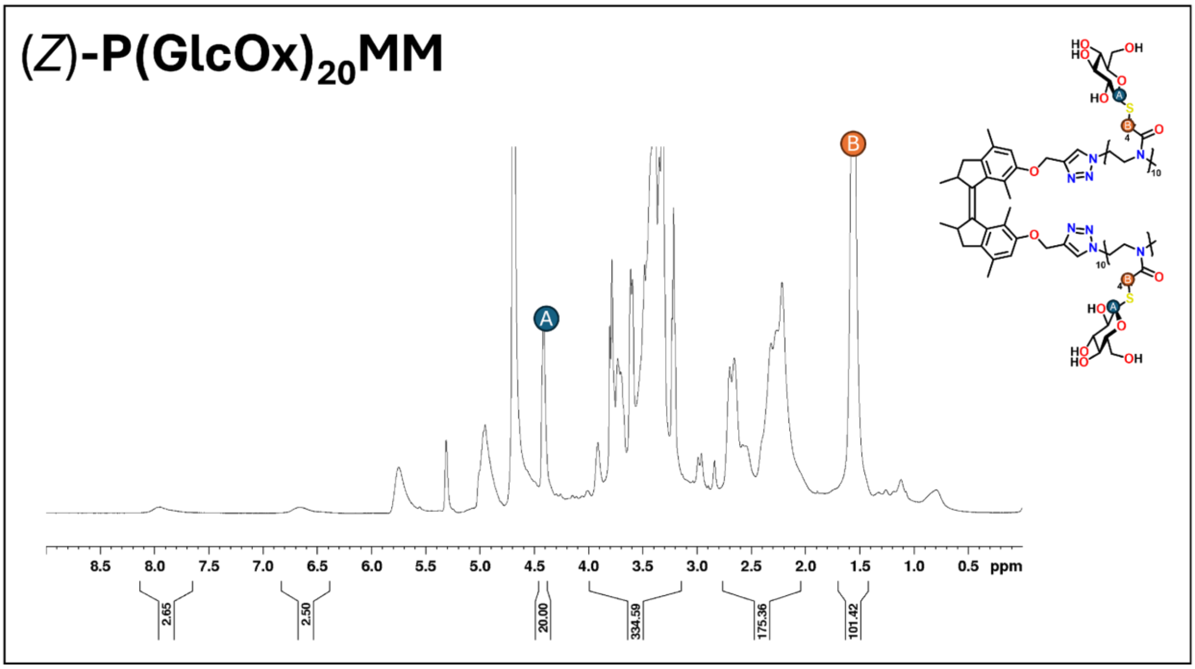

Figure S44. The partially assigned ^{1}H NMR spectrum of *(Z)*-**P(GlcOx)₂₀MM** (600 MHz, $D_2O:CD_3OD$ 99:1 V:V, 25 °C).

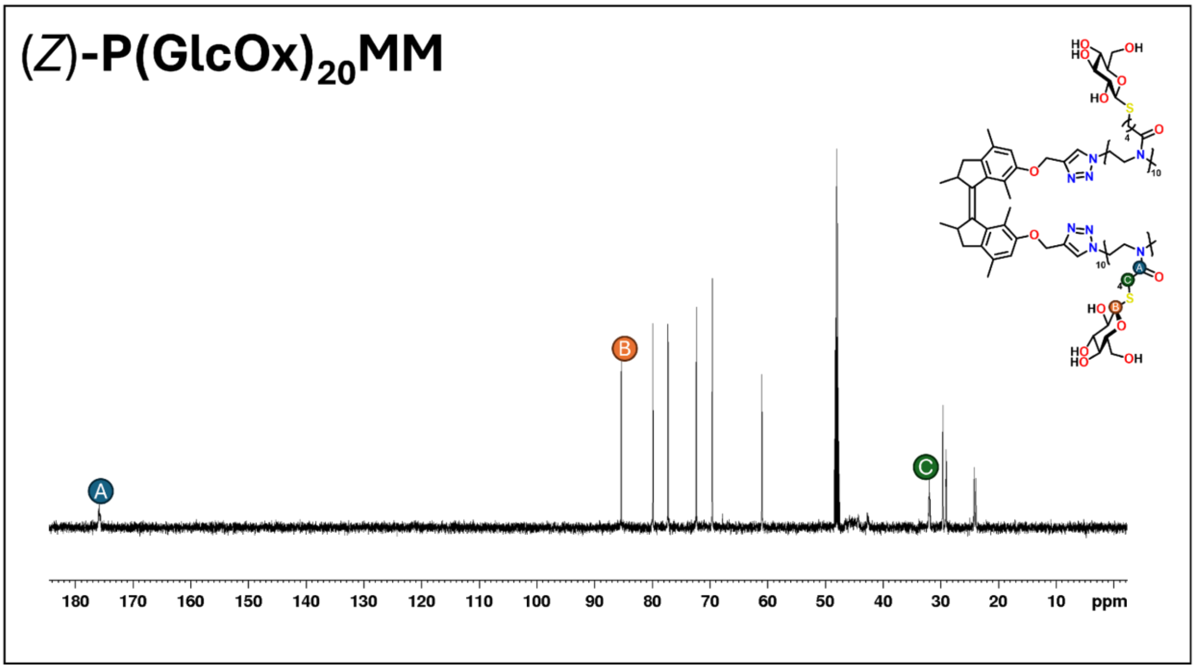

Figure S45. The partially assigned ^{13}C NMR spectrum of *(Z)*-**P(GlcOx)$_{20}$MM** (151 MHz, $D_2O:CD_3OD$ 99:1 V:V, 25°C).

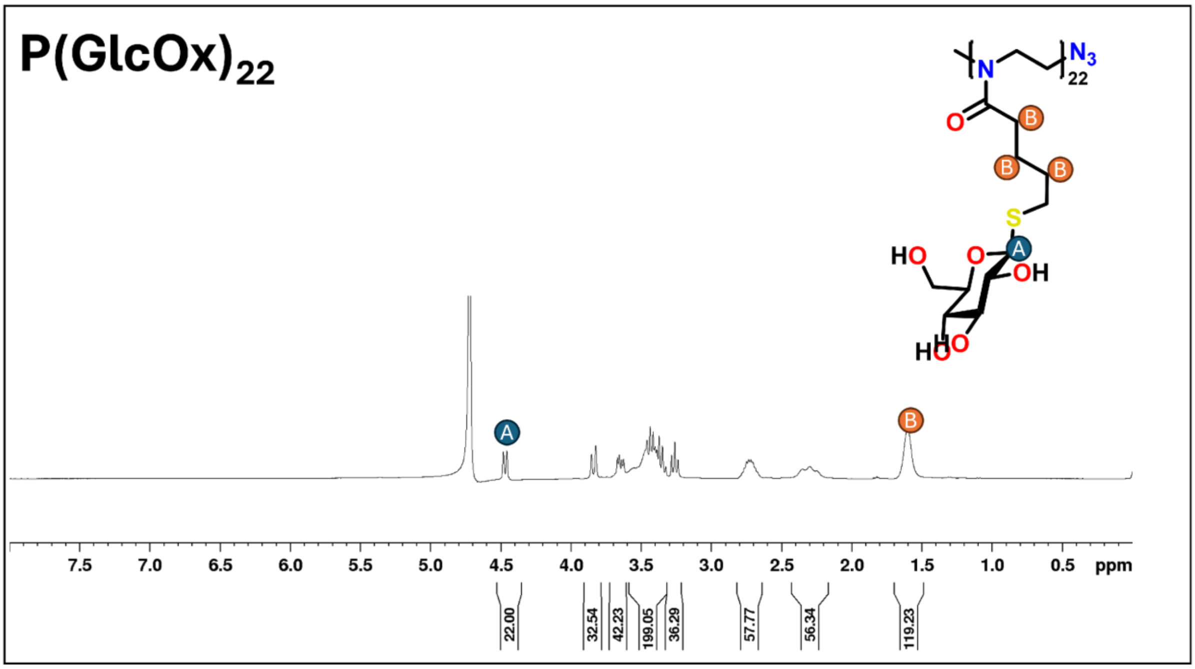

Figure S46. The partially assigned ^{1}H NMR spectrum of **P(GlcOx)$_{22}$** (600 MHz, D_2O:CD_3OD 99:1 V:V, 25 °C).

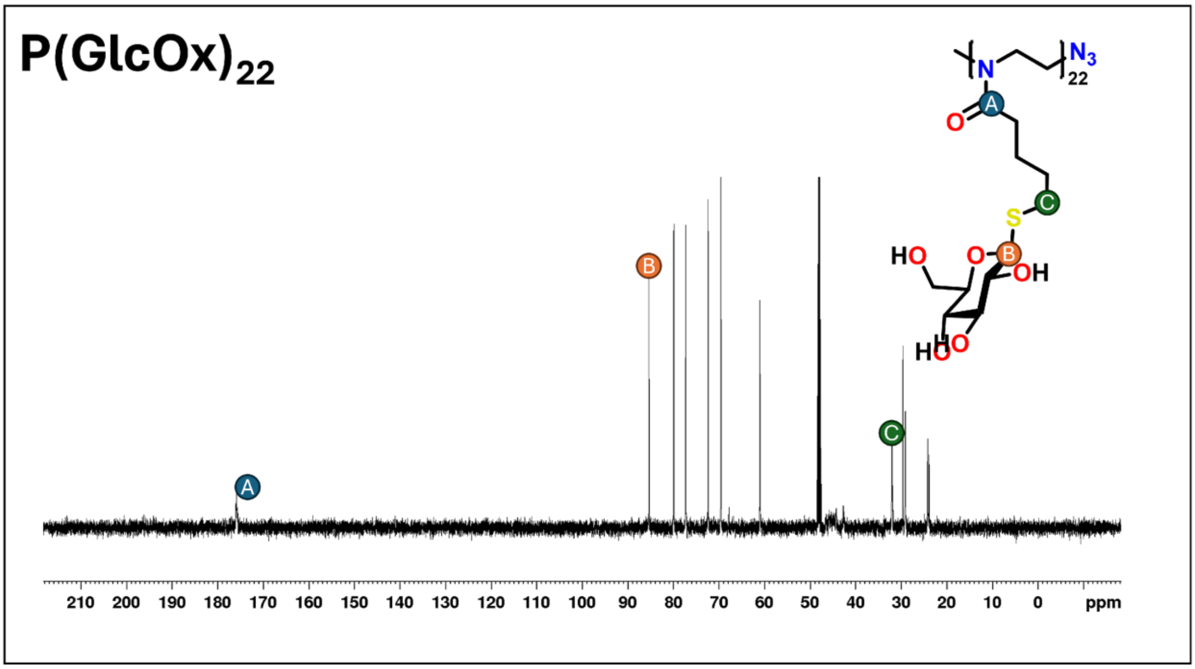

Figure S47. The partially assigned ^{13}C NMR spectrum of **P(GlcOx)$_{22}$** (151 MHz, D_2O:CD_3OD 99:1 V:V, 25°C).

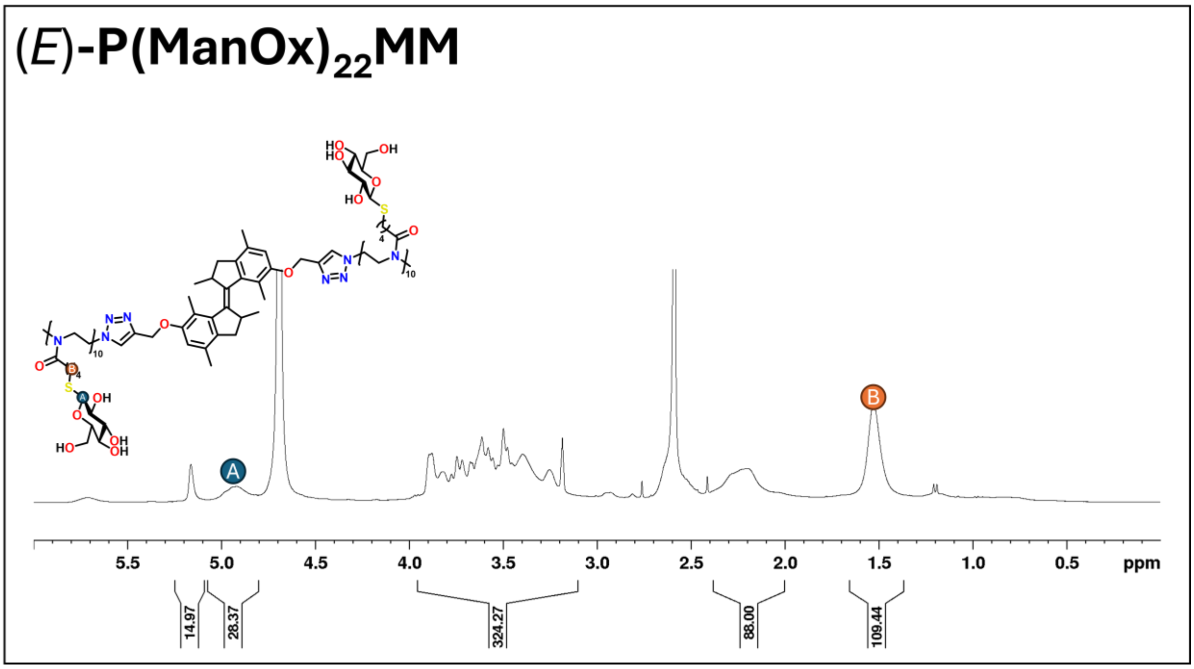

Figure S48. The partially assigned ^{1}H NMR spectrum of *(E)*-**P(ManOx)$_{22}$MM** (400 MHz, D_2O:CD_3OD 9:1 V:V, 25 °C).

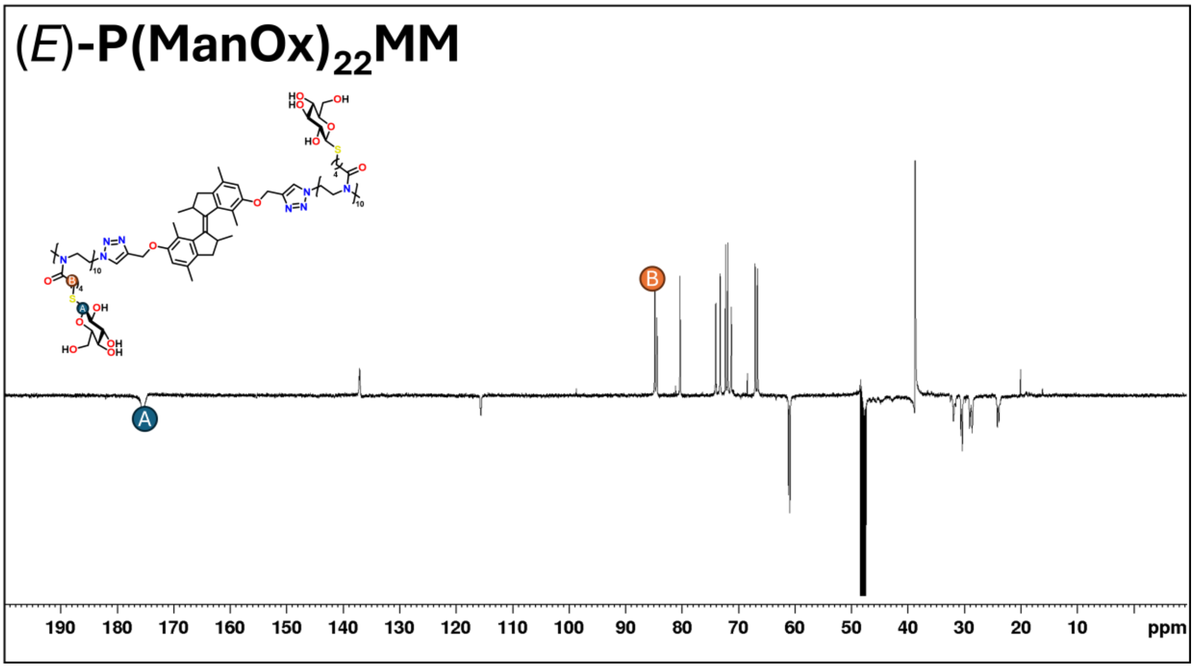

Figure S49. The partially assigned ^{13}C NMR spectrum of *(E)*-**P(ManOx)$_{22}$MM** (151 MHz, D_2O:CD_3OD 9:1 V:V, 25°C).

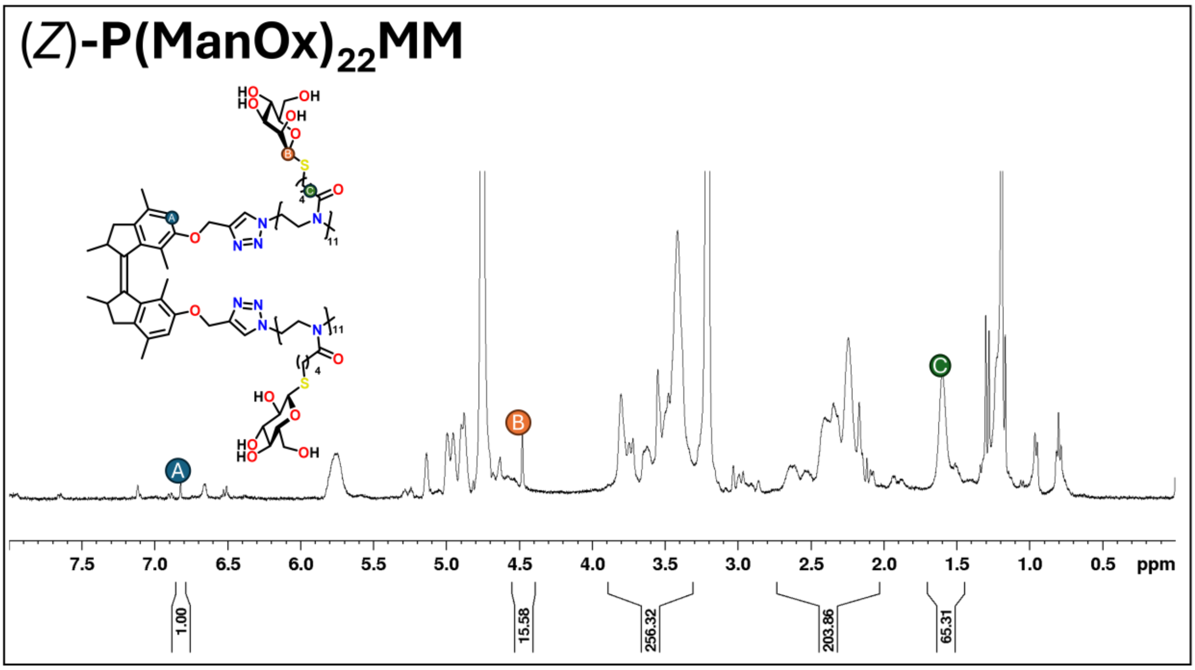

Figure S50. The partially assigned ^{1}H NMR spectrum of *(Z)*-**P(ManOx)$_{22}$MM** (400 MHz, D_2O:CD_3OD 9:1 V:V, 25 °C).

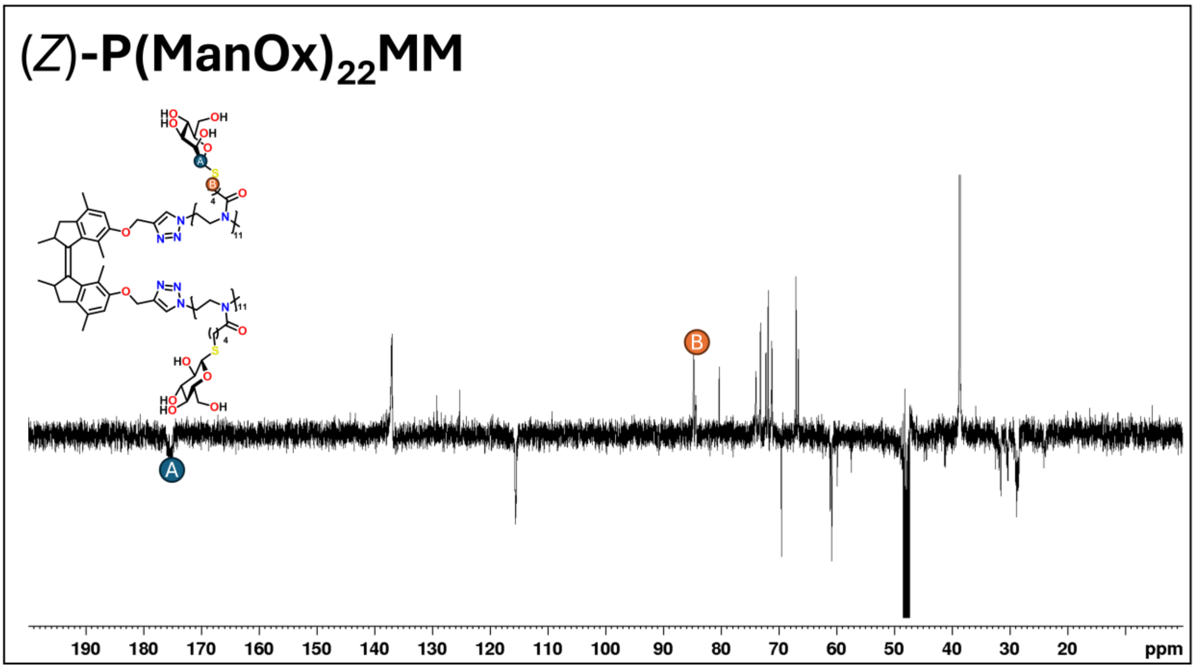

Figure S51. The partially assigned ^{13}C NMR spectrum of *(Z)*-**P(ManOx)$_{22}$MM** (151 MHz, D_2O:CD_3OD 9:1 V:V, 25°C).

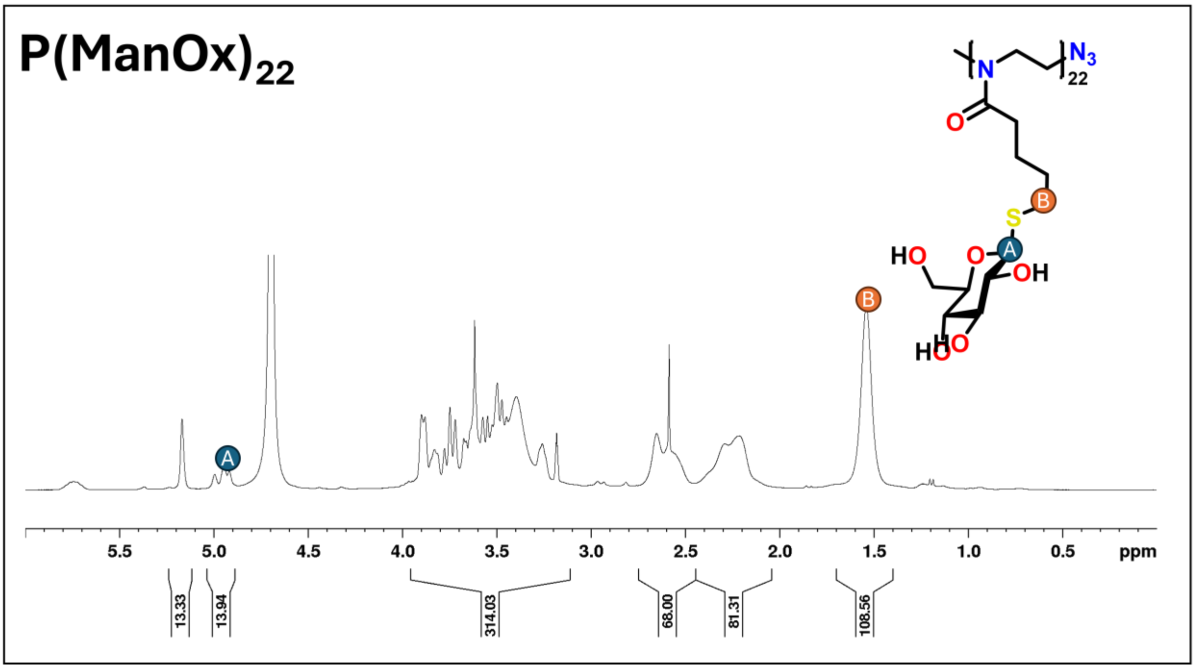

Figure S52. The partially assigned ^{1}H NMR spectrum of **P(ManOx)22** (400 MHz, D_2O:CD_3OD 9:1 V:V, 25 °C).

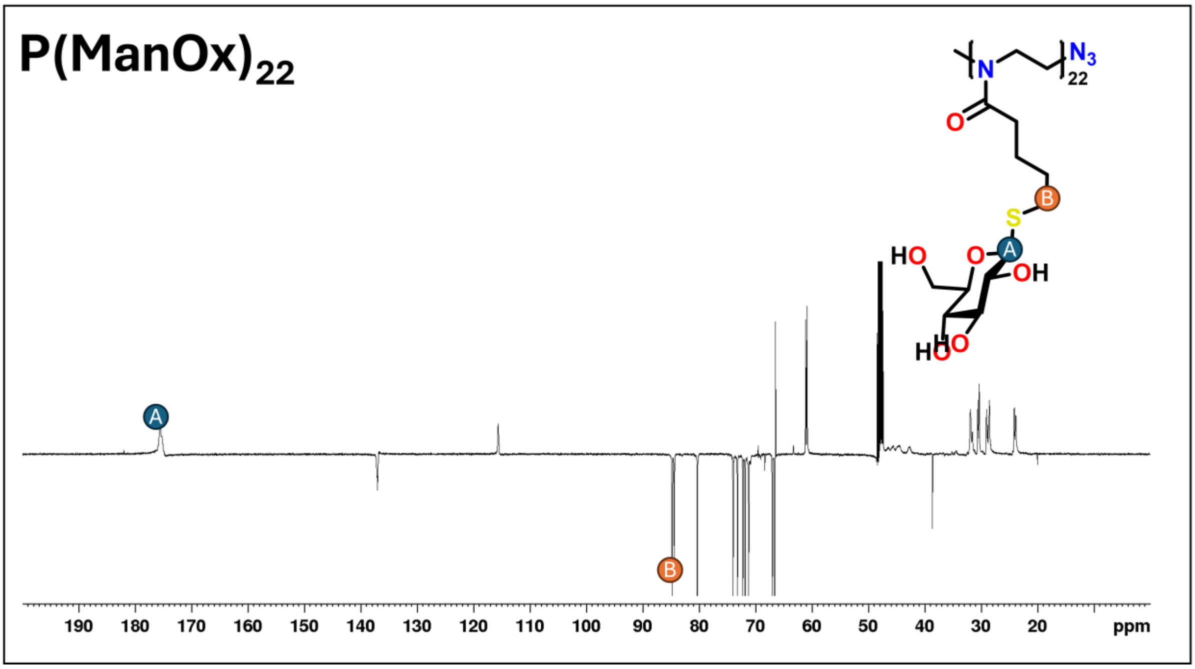

Figure S53. The partially assigned ^{13}C NMR spectrum of **P(ManOx)22** (151 MHz, D_2O:CD_3OD 99:1 V:V, 25°C).

6.2. Gel permeation chromatography (GPC) analysis

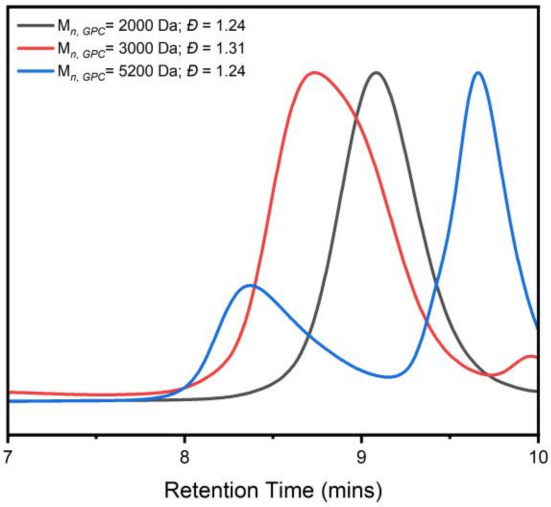

Figure S54. GPC traces (THF) associated with **P(^{n}ButeneOx)$_{10}$** (black), *(E)*-**D1** (red) and *(E)*-**D2** (blue).

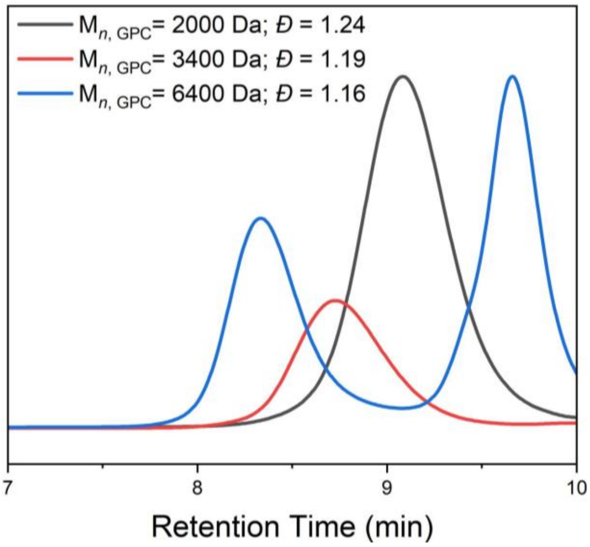

Figure S55. GPC traces (THF) associated with **P(^{n}ButeneOx)$_{10}$** (black), *(Z)*-**D1** (red) and *(Z)*-**D2** (blue).

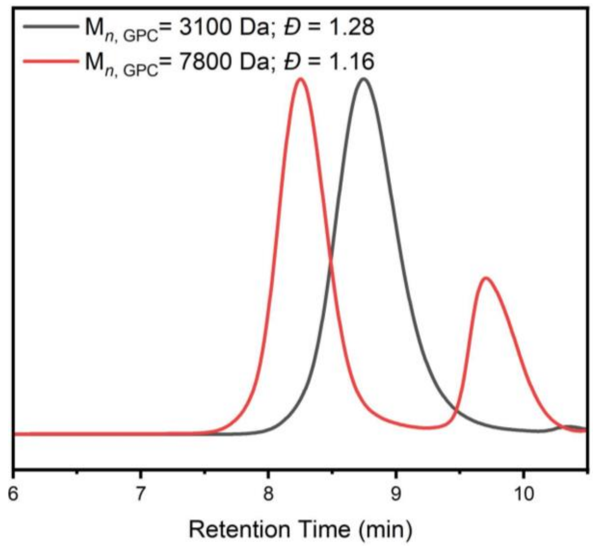

Figure S56. GPC traces (THF) associated with **P(^{n}ButeneOx)$_{22}$** (black) and **P(Ac$_4$GlcOx)$_{22}$** (red).

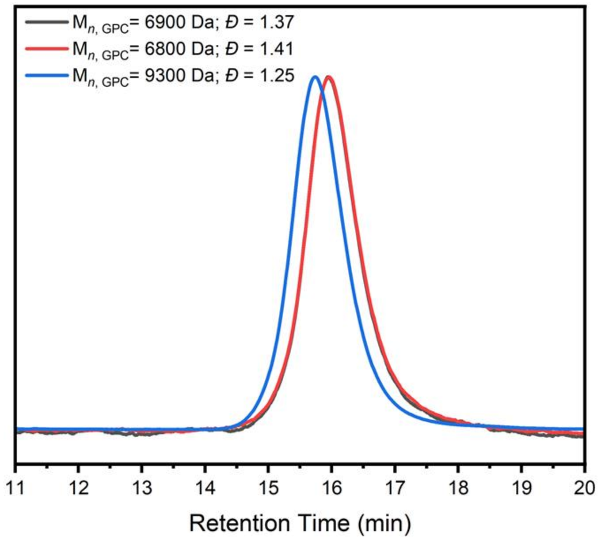

Figure S57. GPC traces (DMF) associated with *(E)*-**P(GlcOx)$_{20}$MM** (black), *(Z)*-**P(GlcOx)$_{20}$MM** (red) and **P(GlcOx)$_{22}$** (blue).

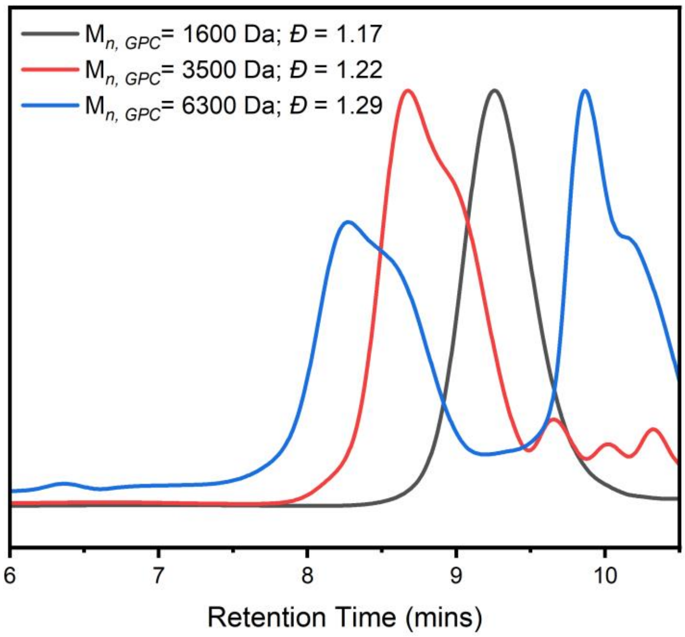

Figure S58. GPC traces (THF) associated with **P(^{n}ButeneOx)$_{11}$** (black), *(E)*-**D1** (red) and *(E)*-**D2** (blue).

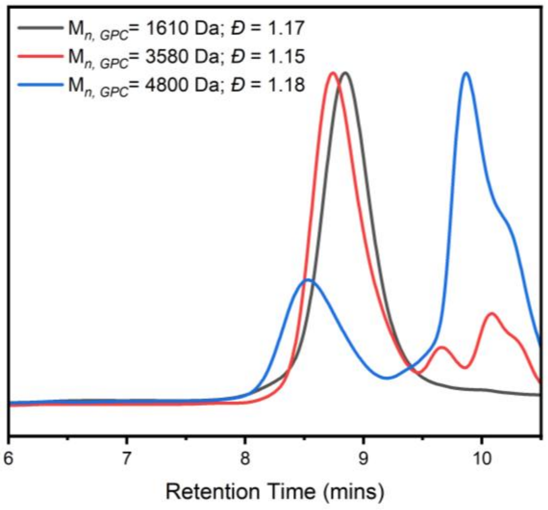

Figure S59. GPC traces (THF) associated with **P(^{n}ButeneOx)$_{11}$** (black), *(Z)*-**D1** (red) and *(Z)*-**D2** (blue).

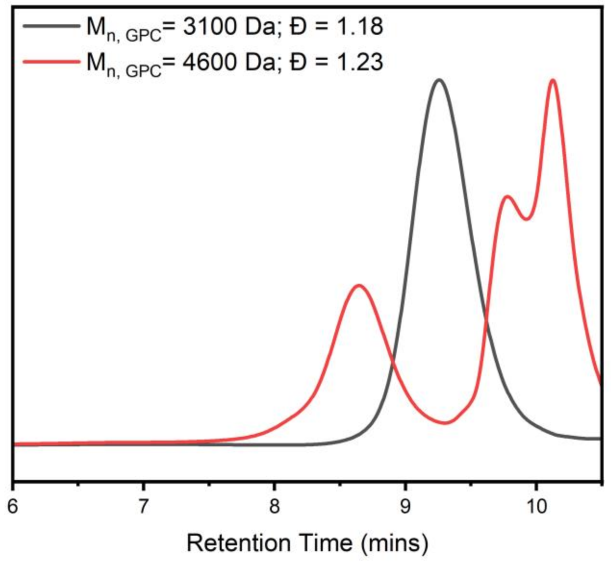

Figure S60. GPC traces (THF) associated with **P(^{n}ButeneOx)$_{22}$** (black) and **P(Ac$_4$ManOx)$_{22}$** (red).

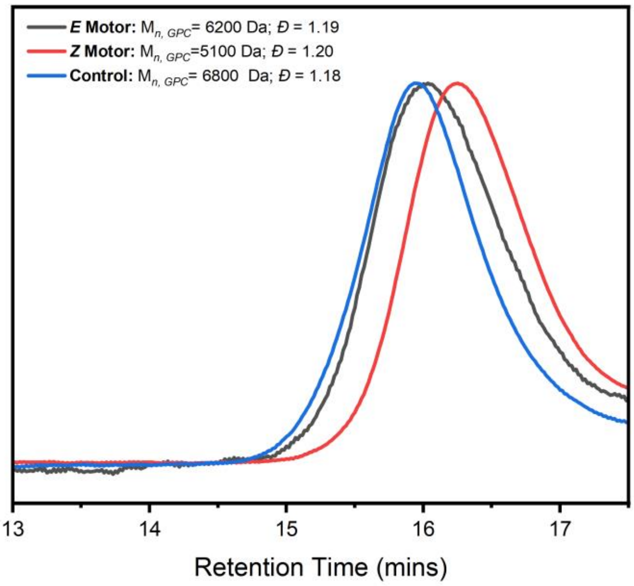

Figure S61. GPC traces (DMF) associated with *(E)*-**P(ManOx)$_{22}$MM** (black), *(Z)*-**P(ManOx)$_{22}$MM** (red) and **P(ManOx)$_{22}$** (blue).

6.3. Fourier-transform infrared (FT-IR) spectra

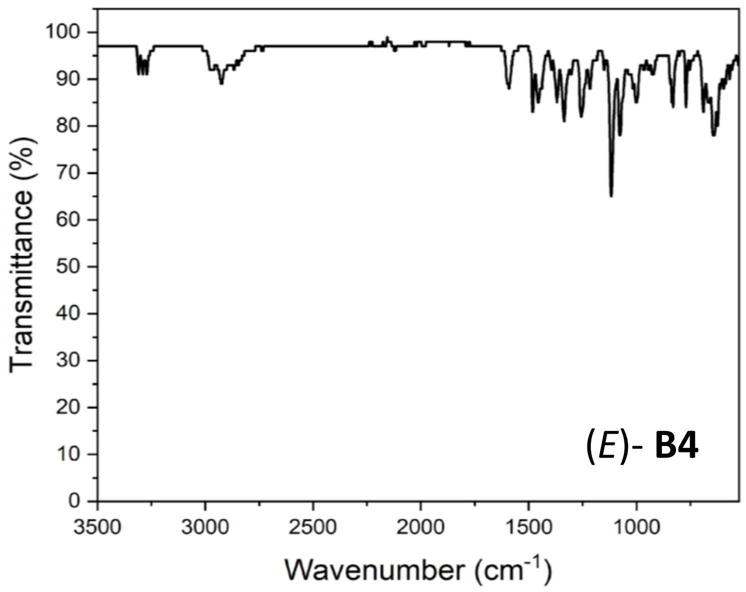

Figure S62. FT-IR spectrum associated with bis-propargyl motor *(E)*-**B4**.

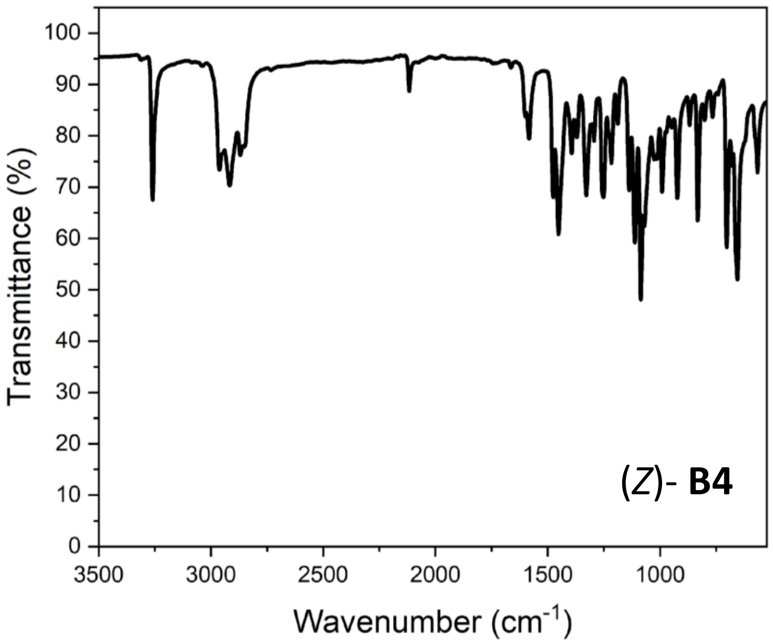

Figure S63. FT-IR spectrum associated with bis-propargyl motor *(Z)*-**B4**.

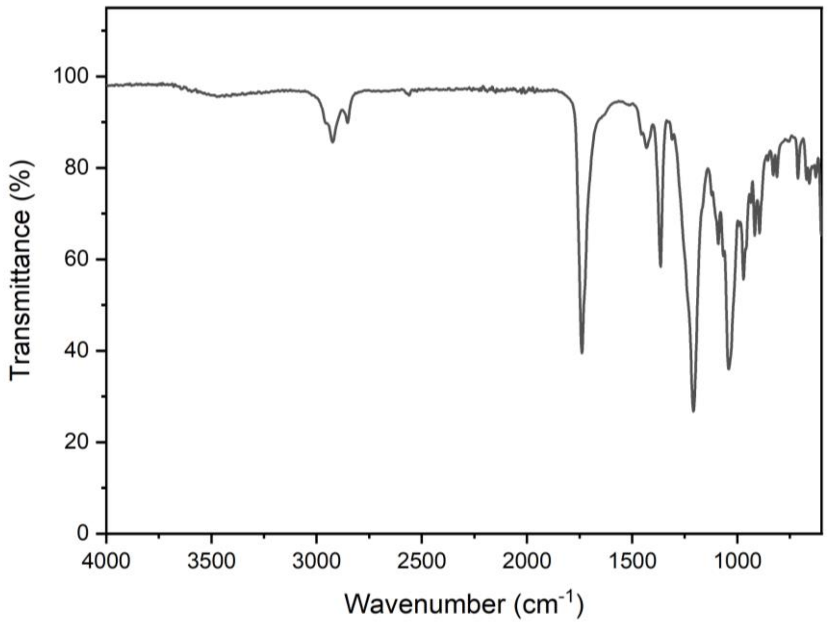

Figure S64. FT-IR spectrum associated with 1-Thio-*β*-*D*-Mannopyranose tetra-*O*-acetate.

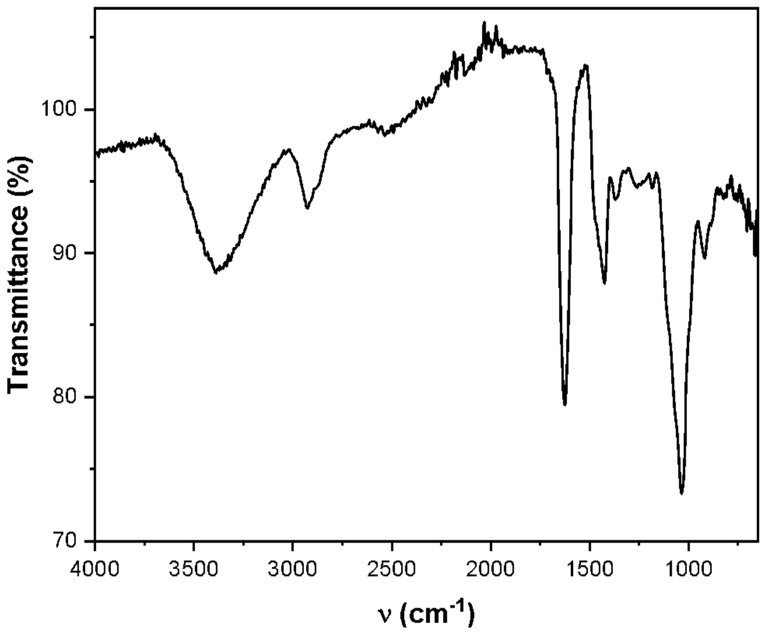

Figure S65. FT-IR spectrum associated with motor *(E)*-**P(GlcOx)$_{20}$MM**.

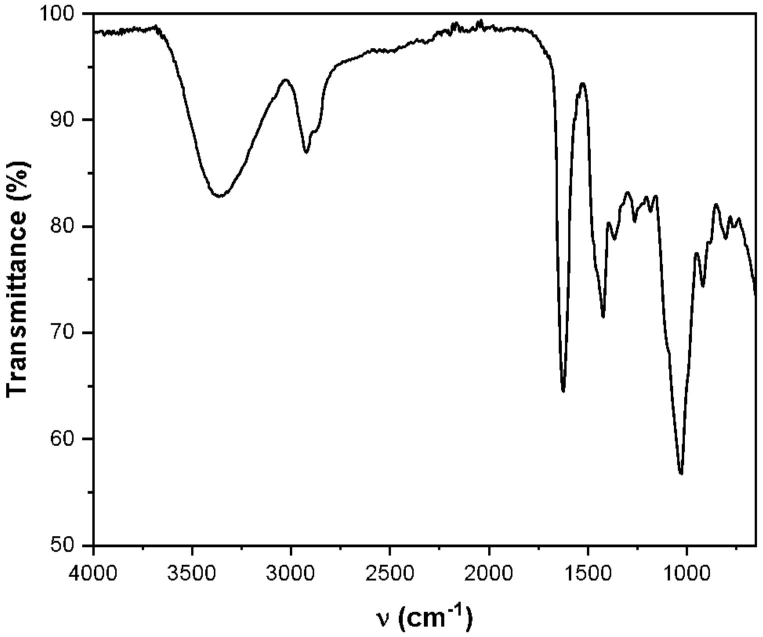

Figure S66. FT-IR spectrum associated with motor *(Z)*-**P(GlcOx)$_{20}$MM**.

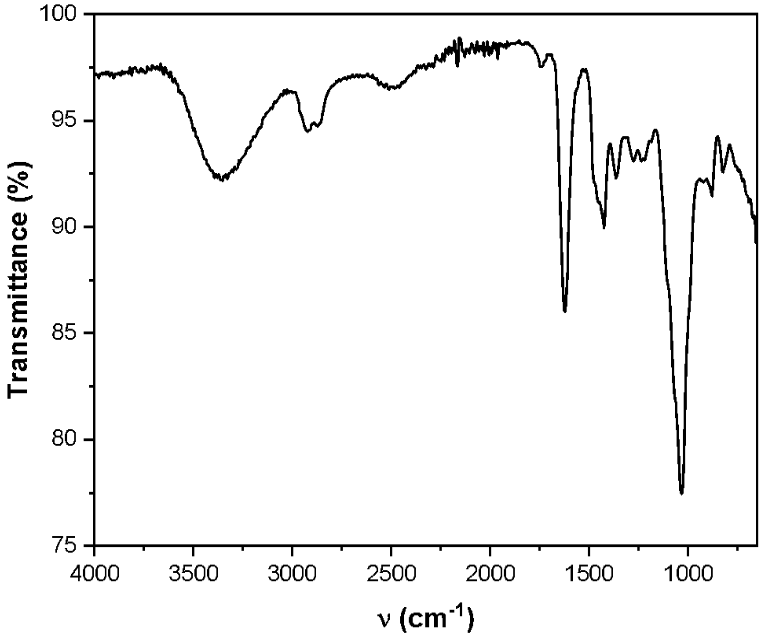

Figure S67. FT-IR spectrum associated with glycopolymer **P(GlcOx)$_{22}$**.

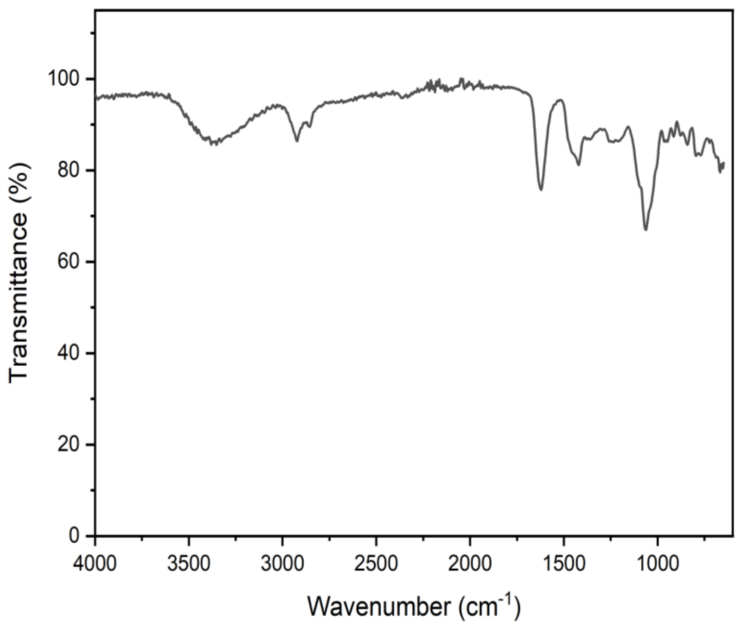

Figure S68. FT-IR spectrum associated with motor *(E)*-**P(ManOx)$_{22}$MM**.

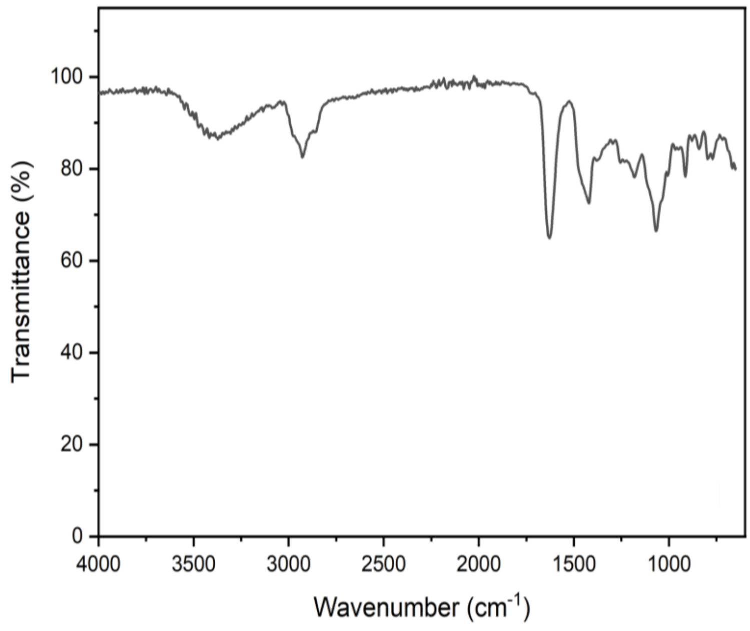

Figure S69. FT-IR spectrum associated with motor *(Z)*-**P(ManOx)$_{22}$MM**.

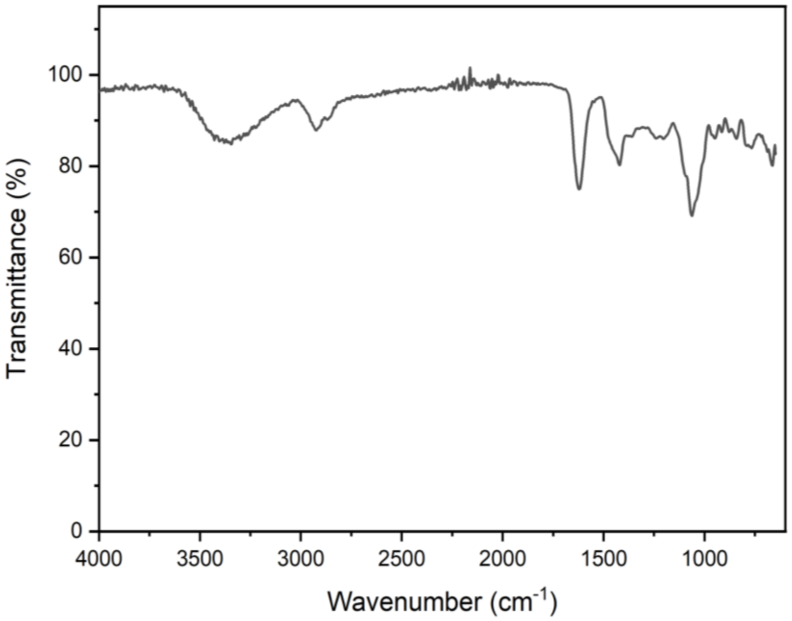

Figure S70. FT-IR spectrum associated with glycopolymer **P(ManOx)$_{22}$**.

6.4. Matrix assisted laser deabsorption ionization - time of flight (MALDI-ToF)

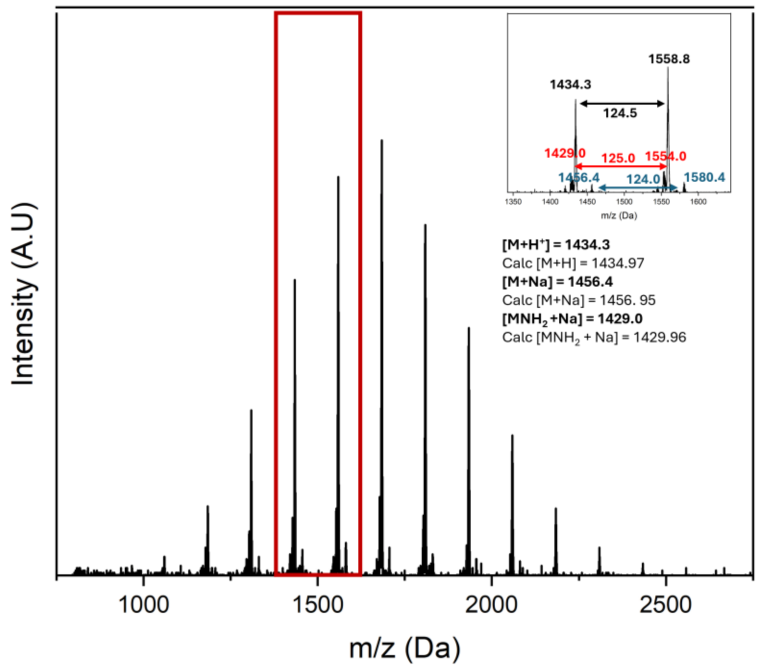

Figure S71. MALDI-ToF spectrum of **P(^{n}ButeneOx)$_{11}$**.

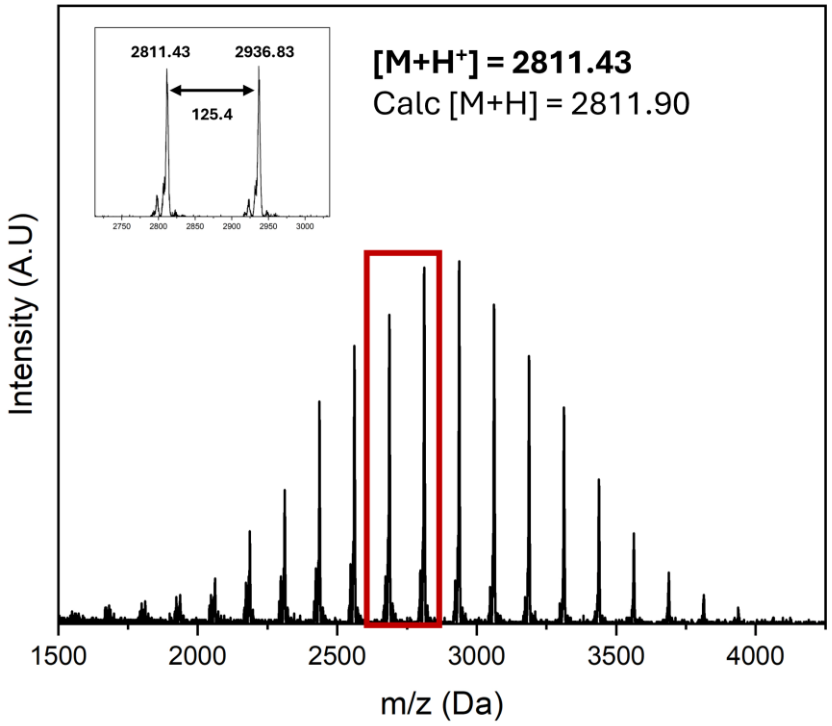

Figure S72. MALDI-ToF spectrum of P(**ButeneOx)$_{22}$.**

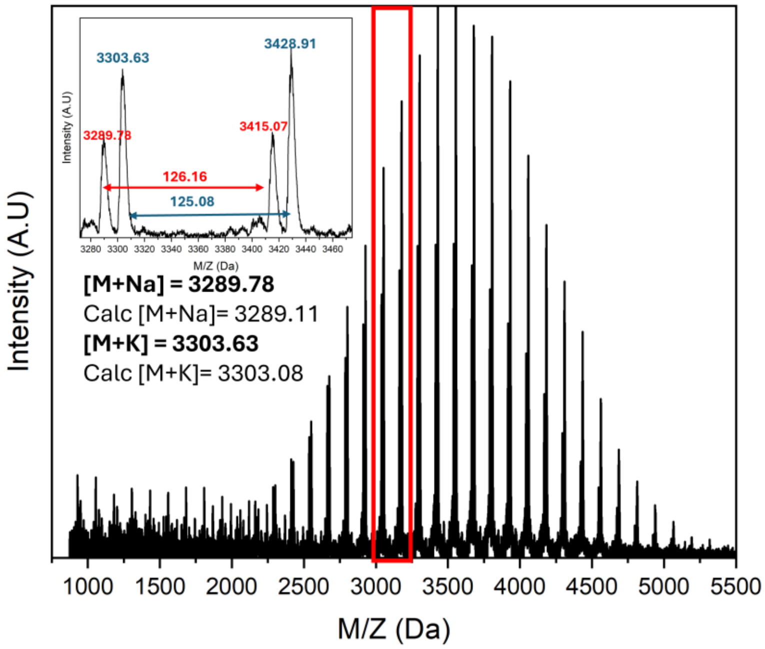

Figure S73. MALDI-ToF spectrum of **(Z)-(ButeneOx)$_{22}$MM**.

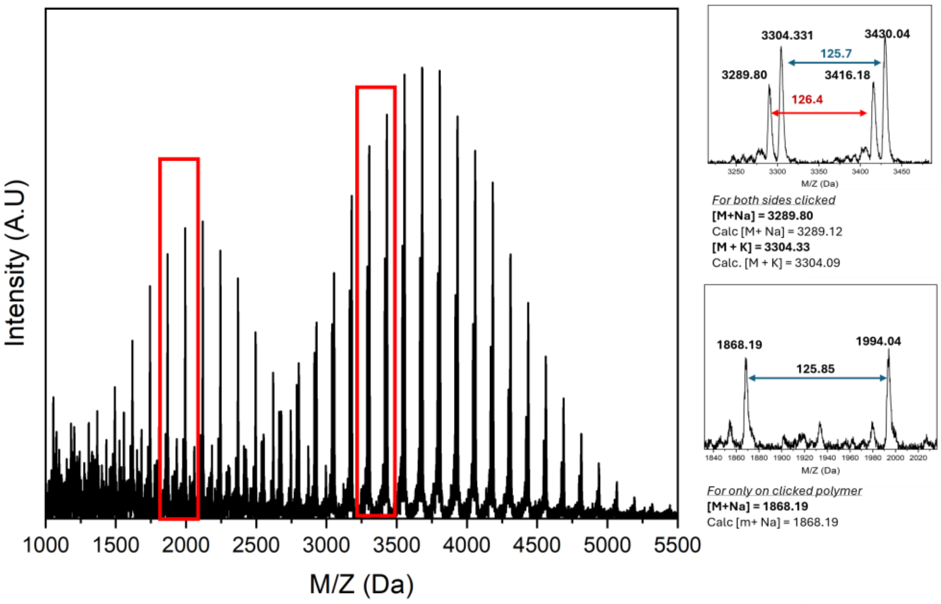

Figure S74. MALDI-ToF of **(*E*)-(ButeneOx)$_{22}$MM**.

7. References

(1) Gress, A.; Völkel, A.; Schlaad, H. Thio-Click Modification of Poly[2-(3-butenyl)-2-oxazoline]. *Macromolecules* **2007**, *40*, 7928–7933.

(2) van Leeuwen, T.; Neubauer, T. M.; Feringa B. L. Regioselective Synthesis of Indanones. *Synlett* **2014**, *25*, 1717–1720.

(3) Neubauer, T. M.; van Leeuwen, T.; Zhao, D.; Lubbe, A. S.; Kistemaker, J. C.; Feringa, B. L. Asymmetric synthesis of first generation molecular motors. *Org. Lett.* **2014**, *16*, 4220–4223.

(4) van Leeuwen, T.; Gan, J.; Kistemaker, J. C.; Pizzolato, S. F.; Chang, M. C.; Feringa, B. L. Enantiopure Functional Molecular Motors Obtained by a Switchable Chiral-Resolution Process. *Chem. Eur. J.* **2016**, *22*, 7054–7058.

(5) Hartweg, M.; Jiang, Y.; Yilmaz, G.; Jarvis, C. M.; Nguyen, H. V.; Primo, G. A.; Monaco, A.; Beyer, V. P.; Chen, K. K.; Mohapatra, S.; Axelrod, S.; Gómez-Bombarelli, R.; Kiessling, L. L.; Becer, C. R.; Johnson, J. A. Synthetic Glycomacromolecules of Defined Valency, Absolute Configuration, and Topology Distinguish between Human Lectins. *JACS Au* **2021**, *1*, 1621–1630.

(6) Ishido, Y.; Kanbayashi, N.; Fujii, N.; Okamura, T. A.; Haino, T.; Onitsuka, K. Folding control of a non-natural glycopeptide using saccharide-coded structural information for polypeptides. *Chem. Commun.* **2020**, *56*, 2767–2770.

(7) Shu, P.; Zeng, J.; Tao, J.; Zhao, Y.; Yao, G.; Wan, Q. Selective *S*-deacetylation inspired by native chemical ligation: practical syntheses of glycosyl thiols and drug mercapto-analogues. *Green Chem.* **2015**, *17*, 2545–2551.

(8) Stuart, M. C. A.; van de Pas, J. C.; Engberts, J. B. F. N. The use of Nile Red to monitor the aggregation behavior in ternary surfactant–water–organic solvent systems. *J. Phys. Org. Chem.* **2005**, *18*, 929–934.

(9) Wang, J.; Feringa, B. L. Dynamic Control of Chiral Space in a Catalytic Asymmetric Reaction Using a Molecular Motor. *Science* **2011**, *331*, 1429–1432.

(10) Lubbe, A. S.; Böhmer, C.; Tosi, F.; Szymański, W.; Feringa, B. L. Molecular Motors in Aqueous Environment. *J. Org. Chem.* **2018**, *83*, 11008–11018.
